# The carboxy terminus causes interfacial assembly of oleate hydratase on a membrane bilayer

**DOI:** 10.1016/j.jbc.2024.105627

**Published:** 2024-01-10

**Authors:** Christopher D. Radka, Christy R. Grace, Hale S. Hasdemir, Yupeng Li, Carlos C. Rodriguez, Patrick Rodrigues, Michael L. Oldham, M. Zuhaib Qayyum, Aaron Pitre, William J. MacCain, Ravi C. Kalathur, Emad Tajkhorshid, Charles O. Rock

**Affiliations:** 1 Department of Microbiology, Immunology, and Molecular Genetics, University of Kentucky, Lexington, Kentucky, USA; 2 Department of Host Microbe Interactions, St Jude Children’s Research Hospital, Memphis, Tennessee, USA; 3 Department of Structural Biology, St Jude Children’s Research Hospital, Memphis, Tennessee, USA; 4 Theoretical and Computational Biophysics Group, Department of Biochemistry, and Center for Biophysics and Quantitative Biology, NIH Resource for Macromolecular Modeling and Visualization, Beckman Institute for Advanced Science and Technology, University of Illinois at Urbana-Champaign, Urbana, Illinois, USA; 5 Hartwell Center of Biotechnology, St Jude Children’s Research Hospital, Memphis, Tennessee, USA; 6 Cell and Tissue Imaging Center, St Jude Children’s Research Hospital, Memphis, Tennessee, USA

**Keywords:** oleate hydratase (OhyA), phospholipid, membrane, lipid binding protein, lipid-protein interaction, protein structure, structure-function, amphipathic helices, interfacial enzyme, peripheral membrane protein

## Abstract

The soluble flavoprotein oleate hydratase (OhyA) hydrates the 9-*cis* double bond of unsaturated fatty acids. OhyA substrates are embedded in membrane bilayers; OhyA must remove the fatty acid from the bilayer and enclose it in the active site. Here, we show that the positively charged helix-turn-helix motif in the carboxy terminus (CTD) is responsible for interacting with the negatively charged phosphatidylglycerol (PG) bilayer. Super-resolution microscopy of *Staphylococcus aureus* cells expressing green fluorescent protein fused to OhyA or the CTD sequence shows subcellular localization along the cellular boundary, indicating OhyA is membrane-associated and the CTD sequence is sufficient for membrane recruitment. Using cryo-electron microscopy, we solved the OhyA dimer structure and conducted 3D variability analysis of the reconstructions to assess CTD flexibility. Our surface plasmon resonance experiments corroborated that OhyA binds the PG bilayer with nanomolar affinity and we found the CTD sequence has intrinsic PG binding properties. We determined that the nuclear magnetic resonance structure of a peptide containing the CTD sequence resembles the OhyA crystal structure. We observed intermolecular NOE from PG liposome protons next to the phosphate group to the CTD peptide. The addition of paramagnetic MnCl_2_ indicated the CTD peptide binds the PG surface but does not insert into the bilayer. Molecular dynamics simulations, supported by site-directed mutagenesis experiments, identify key residues in the helix-turn-helix that drive membrane association. The data show that the OhyA CTD binds the phosphate layer of the PG surface to obtain bilayer-embedded unsaturated fatty acids.

Oleate hydratase (OhyA) is a bacterial flavoenzyme that catalyzes water addition to C-C double bonds of unsaturated fatty acids to produce hydroxylated fatty acids (*h*FA) (1). OhyA activity is an important step in the metabolic biotransformation of dietary linoleic acid by symbiotic intestinal bacteria (2) and for industrial applications as a biocatalyst to produce *h*FA intermediates (3, 4). The role of OhyA as a virulence determinant has recently become apparent from studies of the major human pathogen *Staphylococcus aureus*, which is a leading cause of skin infection (5). *S. aureus* utilizes OhyA to detoxify antimicrobial fatty acids on the skin that are a component of innate immunity and release extracellular nontoxic *h*FA (6). OhyA activity antagonizes the immune response to *S. aureus* in a murine skin infection model (7), and its disruption compromises *S. aureus* virulence in murine skin (7) and rabbit endocarditis infection models (8). These studies establish the role of OhyA in promoting pathogenesis by dampening the immune system (Fig. 1*A*).

**Figure 1:**
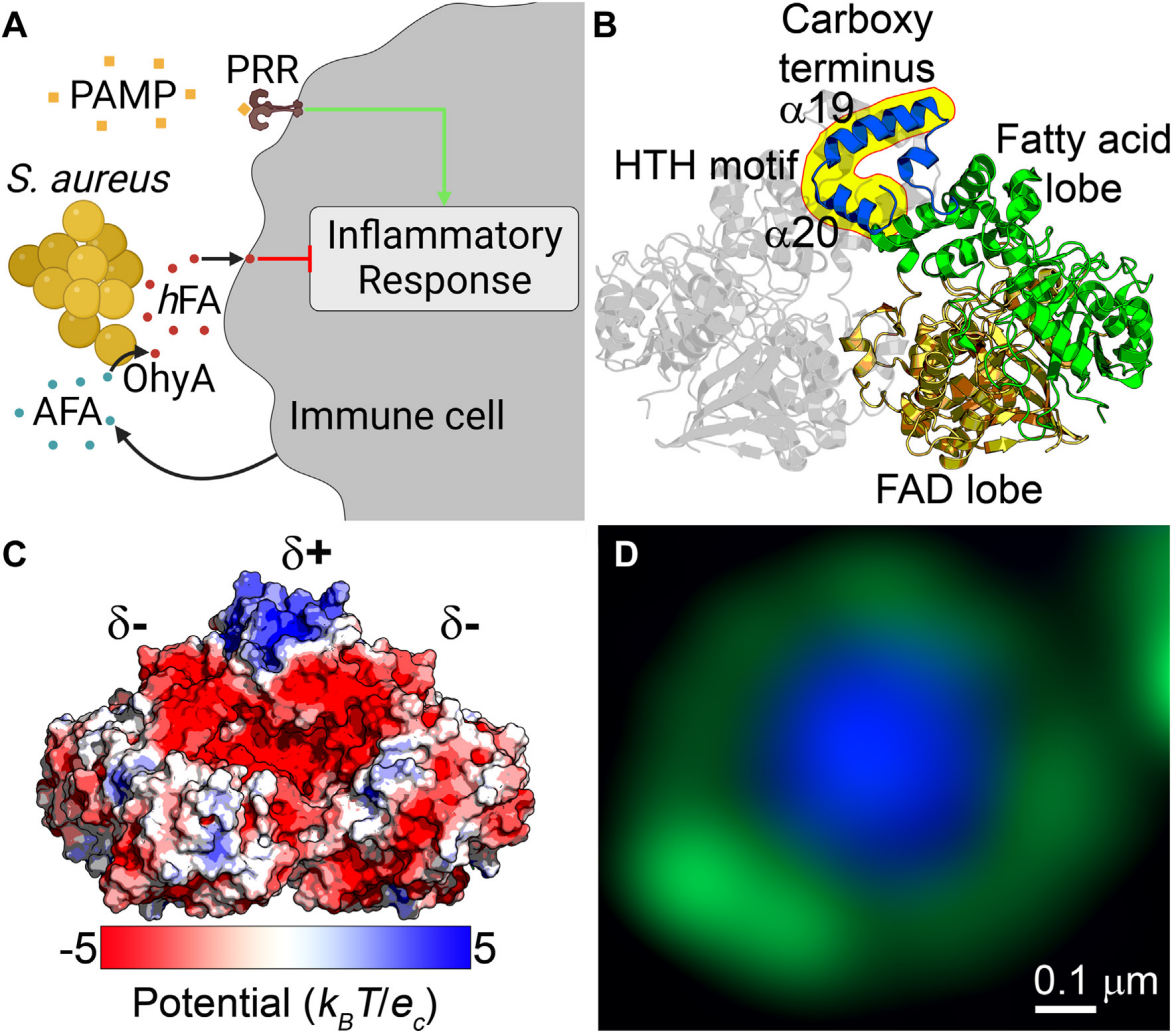
**OhyA function, structure, and subcellular localization.***A*, model for anti-inflammatory OhyA signaling. *Staphylococcus aureus* utilizes OhyA to convert antimicrobial fatty acids (AFA) to hydroxy fatty acids (*h*FA) that blunt the inflammatory response to pathogen-associated molecular pattern (PAMP)/pathogen recognition receptor (PRR) signaling cascades in a skin infection. *B*, crystal structure of OhyA (PDB ID: 7KAV) colored by functional domain. The carboxy terminus contains two amphipathic α-helices (α-19 and α-20) that make up the helix-turn-helix (HTH) motif (*yellow shading*). *C*, electrostatic surface rendering of OhyA. The carboxy terminus contains a positive electrostatic patch. *D*, super resolution AiryScan image of *S. aureus* RN4220 strain CDR001 expressing GFP-OhyA, counter stained with DAPI (two merged channels). GFP-OhyA is localized along the cellular boundary. The scale bar corresponds to 0.1 μm. The Fig. S2 contains the individual channels of this cell (Fig. S2*A*) and other representative CDR001 cells (Fig. S2, *B* and *C*).

OhyA purifies as a soluble homodimer where each bilobed protomer contains three functional domains (1) (Fig. 1*B*): a fatty acid lobe for binding substrate unsaturated fatty acid, a flavin adenine dinucleotide (FAD) lobe for binding cofactor FAD, and a carboxy terminus of unknown function. Complex crystal structures of *S. aureus* OhyA bound with unsaturated fatty acid (PDB ID: 7KAY) show OhyA binds monomeric fatty acid (1), which is insoluble and spontaneously self-assembles into multimeric structures in water (*e.g.*, dimers (9) and micelles (10)). Phospholipids solubilize fatty acids (11) and unsaturated fatty acids partition in the membrane bilayer in cells. Therefore, aqueous OhyA must solve the topological problem of accessing unsaturated fatty acids stored in membrane bilayers, but the structural feature(s) used to operate at the water-lipid interface and obtain the unsaturated fatty acids are undefined.

Amphipathic helices are well-established structural features used by peripheral proteins to interact with membranes (12). Amphipathic helices can associate with membrane lipids directly by inserting the hydrophobic helical face into the lipid bilayer and associating the polar/charged helical face with polar lipid head groups and water. For instance, the lipid biosynthetic enzyme CTP:phosphocholine cytidylyltransferase uses amphipathic helices to bind membranes in a lipid composition-dependent manner (13, 14, 15). Another example is the Bin/Amphiphysin/Rvsp domains of proteins which are amphipathic helices that bind membranes in a membrane curvature-dependent manner (16, 17, 18). Lipases use mobile amphipathic helix lids to control enzymatic activity by exposing the active site upon encountering lipid substrates in a process called interfacial activation (19). Wootan and Storch (1994) proposed cytosolic fatty acid binding proteins transfer fatty acid by transient collision with lipid bilayers where the protein-membrane association is stabilized by ionic and hydrophobic interactions (20). Positive charges from surface lysine side chains, *e.g.*, in amphipathic helices are important for the direct interaction between fatty acid binding proteins and anionic phospholipids (21, 22, 23).

Each OhyA carboxy terminus is a helical region containing two amphipathic helices (helices α19 and α20) that arrange in a helix-turn-helix (HTH) motif that sits above a hydrophobic cavity from which a tunnel leads to the active site (1). The distribution of amino acids creates a concentrated positive electrostatic potential in the HTH (Fig. 1*C*). The OhyA homodimer has two such amphipathic HTHs that concentrate their positively charged side chains in a hydrophilic side that faces the solvent, and their aliphatic side chains to a hydrophobic side that faces the hydrophobic cavity. The carboxy-terminal HTHs in the crystal structure of *Lactobacillus acidophilus* OhyA (PDB ID: 4IA5) (24) and *S*. *aureus* OhyA (PDB ID: 7KAV) (1) are nearly identical and shield the hydrophobic cavity, whereas the HTHs of *Elizabethkingia meningoseptica* OhyA (PDB ID: 4UIR) are longer and shield the hydrophobic cavity (25). The *Stenotrophomonas* OhyA (PDB ID: 5Z70) has partially resolved HTHs and a flexible region that may shield the hydrophobic cavity as well (26).

In this study, we provide the structural basis for how OhyA interacts with the membrane bilayer and establish OhyA as a peripheral membrane protein. We show amphipathic helices in the carboxy terminus target OhyA to the membrane and calculate membrane-binding affinities for OhyA and a synthetic peptide representing the carboxy terminus by surface plasmon resonance. We identify direct peptide-lipid interactions by nuclear magnetic resonance (NMR), and show that the peptide interacts with the phosphate layer of the membrane by NOE quenching from paramagnetic manganese and circular dichroism (CD). Cryo-electron microscopy (cryo-EM) snapshots of OhyA and phosphatidylglycerol liposomes show the interaction of OhyA with membrane lipids. Molecular dynamics (MD) simulations of spontaneous binding of the HTH motif to the membrane identify key residues in the carboxy terminus that drive membrane association, as corroborated by site-directed mutagenesis experiments. Deletion of the carboxy-terminal end has a negligible effect on the OhyA fold or thermal stability but compromises membrane binding and biochemical catalysis. The heterologous expression of OhyA but not OhyA with a truncated carboxy terminus (OhyA(ΔHTH)), complements a Δ*ohyA* knockout strain of *S. aureus*. Finally, super-resolution microscopy shows the localization of a GFP-OhyA chimera expressed in *S. aureus* cells to the membrane surface. The carboxy terminus amino acid sequence is sufficient to recruit GFP to the membrane surface of *S. aureus* cells. The results show that OhyA is an interfacial enzyme that uses the carboxy terminus to bind the lipid-water interface and access insoluble unsaturated fatty acids.

## Results

### OhyA associates with the membrane compartment of cells

We engineered an *S. aureus* strain that expresses chimeric green fluorescent protein fused to the amino terminus of OhyA (GFP-OhyA) by allelic replacement of *gfp-ohyA* for *ohyA* in the genome for cellular localization studies (Fig. S1). We used super-resolution microscopy as an area-based method to directly measure the location of GFP-OhyA, and observed green rings (Figs. 1*D* and S2). We observed blue globes when cells were treated with the nuclear dye DAPI as a cytosolic control, and we observed red rings when cells were treated with the membrane dye CellBrite Fix 640 as a membrane control (Fig. S2). These data indicate GFP-OhyA is associated with the membrane compartment of cells consistent with a study reporting peripheral localization of OhyA (formerly Sok) using immunofluorescence and membrane localization with cell fractionation and immunoblotting (8).

### Preparation of OhyA and OhyA(ΔHTH)

**Figure 2:**
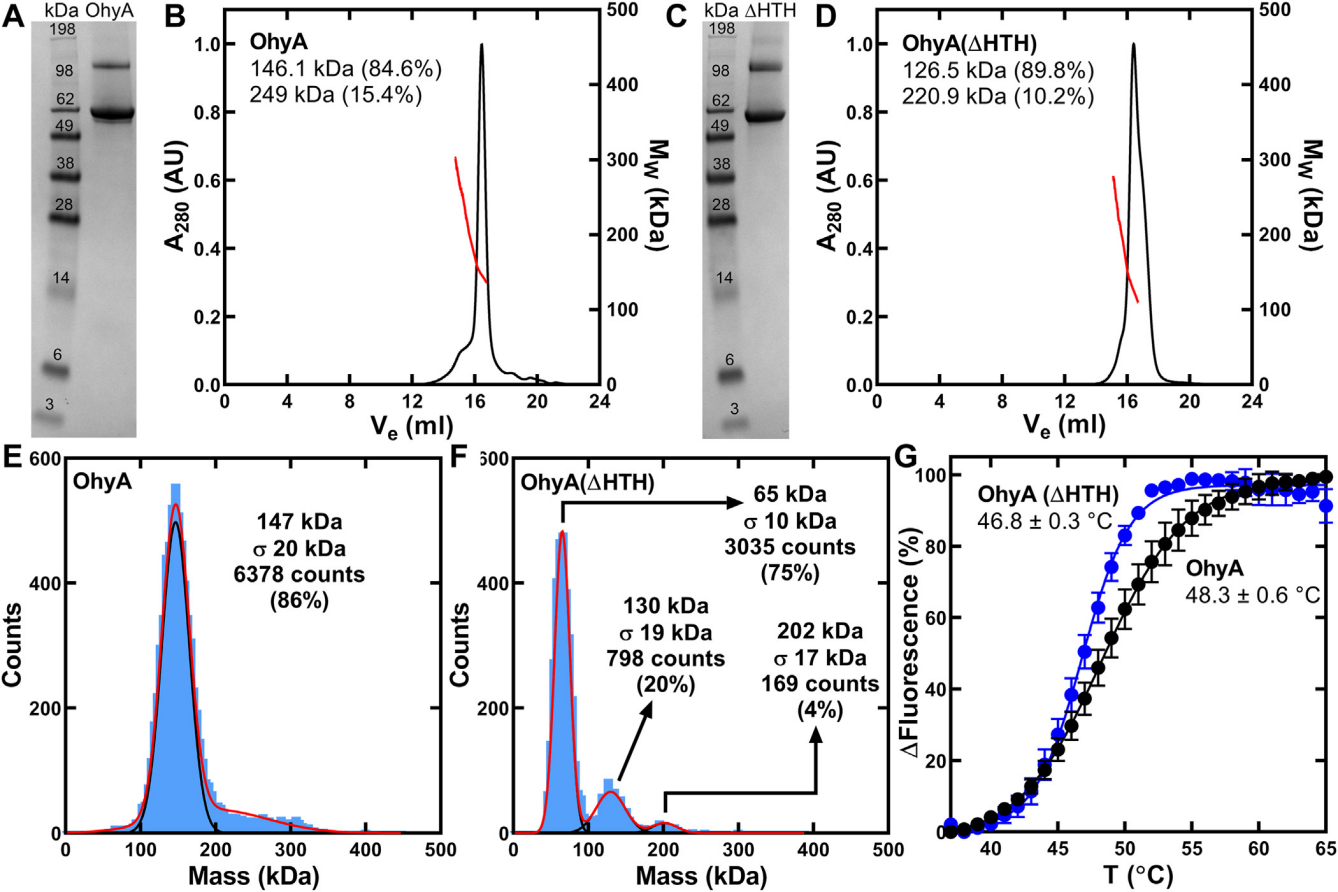
**Purification and thermal stability of OhyA and OhyA(ΔHTH).***A*, OhyA was a 65-kDa monomer and was >95% pure based on denaturing gel electrophoresis. *B*, His-tagged OhyA was purified by Ni^2+^-affinity chromatography followed by gel filtration chromatography on Superose 6 Increase 10/300 Gl and multi-angle light scattering (SEC-MALS). The molar mass distribution (*red line*) is superimposed on a chromatogram of A_280_*versus* elution volume (*black line*). SEC-MALS measured OhyA predominantly migrates as a 146-kDa dimer. *C*, OhyA(ΔHTH) was a 60-kDa monomer and was >95% pure based on denaturing gel electrophoresis. *D*, His-tagged OhyA(ΔHTH) was purified by Ni^2+^-affinity chromatography followed by SEC-MALS using a Superose 6 Increase 10/300 Gl column. The molar mass distribution (*red line*) is superimposed on a chromatogram of A_280_*versus* elution volume (*black line*). SEC-MALS measured OhyA(ΔHTH) predominantly migrates as a 127-kDa dimer. *E*, mass photometry measured OhyA remains a 147-kDa dimer in dilute concentrations. *F*, mass photometry measured OhyA(ΔHTH) is predominantly a 65-kDa monomer in dilute concentrations. *G*, protein thermal denaturation analysis was used to determine the structural integrity of OhyA (*black*) and OhyA(ΔHTH) (*blue*). Assays (n = 5 technical replicates) contained 1 mg/ml OhyA (*black*) of OhyA(ΔHTH) (*blue*). The data are fit to the Boltzmann equation. Mean ± S.D.

Recombinant full-length OhyA and OhyA lacking helices α19 and α20 (OhyA(ΔHTH)) were overexpressed as amino-terminal His-tagged proteins in *Escherichia coli* and purified by Ni^+^ affinity (Fig. 2, *A* and *C*) and gel filtration (Fig. 2, *B* and *D*). The apparent molecular weights estimated by size exclusion chromatography multi-angle light scattering indicate 84.6% of OhyA and 89.8% of OhyA(ΔHTH) are dimers in solution. Mass photometry is a label-free technique that accurately measures the molecular mass of individual molecules in dilute solutions (27). We used mass photometry to evaluate the oligomeric equilibrium (28) of OhyA and OhyA(ΔHTH) and found 86% of OhyA is a dimer (Fig. 2*E*) whereas OhyA(ΔHTH) is 20% dimer and 75% monomer (Fig. 2*F*) under equilibrium conditions. Deletion of helices α19 and α20 has a minor impact on protein thermal stability as the midpoints for the resistance to thermal denaturation are within 1.5 °C of each other for OhyA (48.3 °C) and OhyA(ΔHTH) (46.8 °C) (Fig. 2*G*). These data indicate OhyA and OhyA(ΔHTH) are stable proteins and the carboxy terminus has a concentration-dependent impact on OhyA dimerization.

### Cryo-EM structure of OhyA

OhyA was spotted on a grid for cryo-EM analysis to study the dynamics of the solution structure. Initial micrographs showed particles with severe orientation bias. The addition of 50 μM lauryl maltose neopentyl glycol (LMNG) detergent dispersed the particles, cured the orientation bias, and enabled structure determination of dimeric OhyA to 2.61 Å (Table 1 and Figs. S3 and S4). The OhyA dimer reconstruction was solved using C2 point group symmetry, which matches the C2 symmetry in the OhyA crystal structure (PDB ID: 7KAV) (1). The crystallographic OhyA dimer structure is superimposable with the cryo-EM reconstruction of dimeric OhyA with an RMSD of 0.416 Å, indicating the crystallographic and in-solution structures of the protein are virtually the same.

**Table 1:** Cryo-EM data collection, model composition, refinement, and validation parameters

Structure	OhyA dimer (ordered CTD)	OhyA dimer (disordered CTD)
EMDB ID	EMD-42480	EMD-42484
PDB ID	8UR3	8UR6
Data collection		
Microscope	Talos Arctica (FEI)	Talos Arctica (FEI)
Voltage (kV)	200	200
Detector	K3 BioQuantum (Gatan)	K3 BioQuantum (Gatan)
Pixel size (Å)	1.044	1.044
Defocus range (μM)	−0.5 to −2.0	−0.5 to −2.0
Movies	3246	3246
Frames/movie	50	50
Total dose (electrons/Å^2^)	50	50
Model composition		
Chains	2	2
Non-hydrogen atoms	9376	7540
Protein residues	1158	930
Refinement		
Number of particles	572,989	
Resolution (Å)	2.61	3.03
RMS deviations		
Bond length (Å) (# > 4σ)	0.003 (0)	0.007 (0)
Bond angles (°) (# > 4σ)	0.511 (0)	0.760 (0)
Validation		
MolProbity score	1.32	1.22
Clash score	4.05	4.39
Ramachandran plot (%)		
Outliers	0.00	0.00
Allowed	2.7	1.88
Favored	97.3	98.12
Rotamers (%)		
Outliers	0.0	0.0
Favored	99.7	99.6
Cβ outliers (%)	0	0
Peptide plane (%)		
*Cis* proline/general	0.0/0.2	0.0/0.0
Twisted proline/general	0/0	0/0

### The OhyA carboxy terminus is a mobile domain

**Figure 3:**
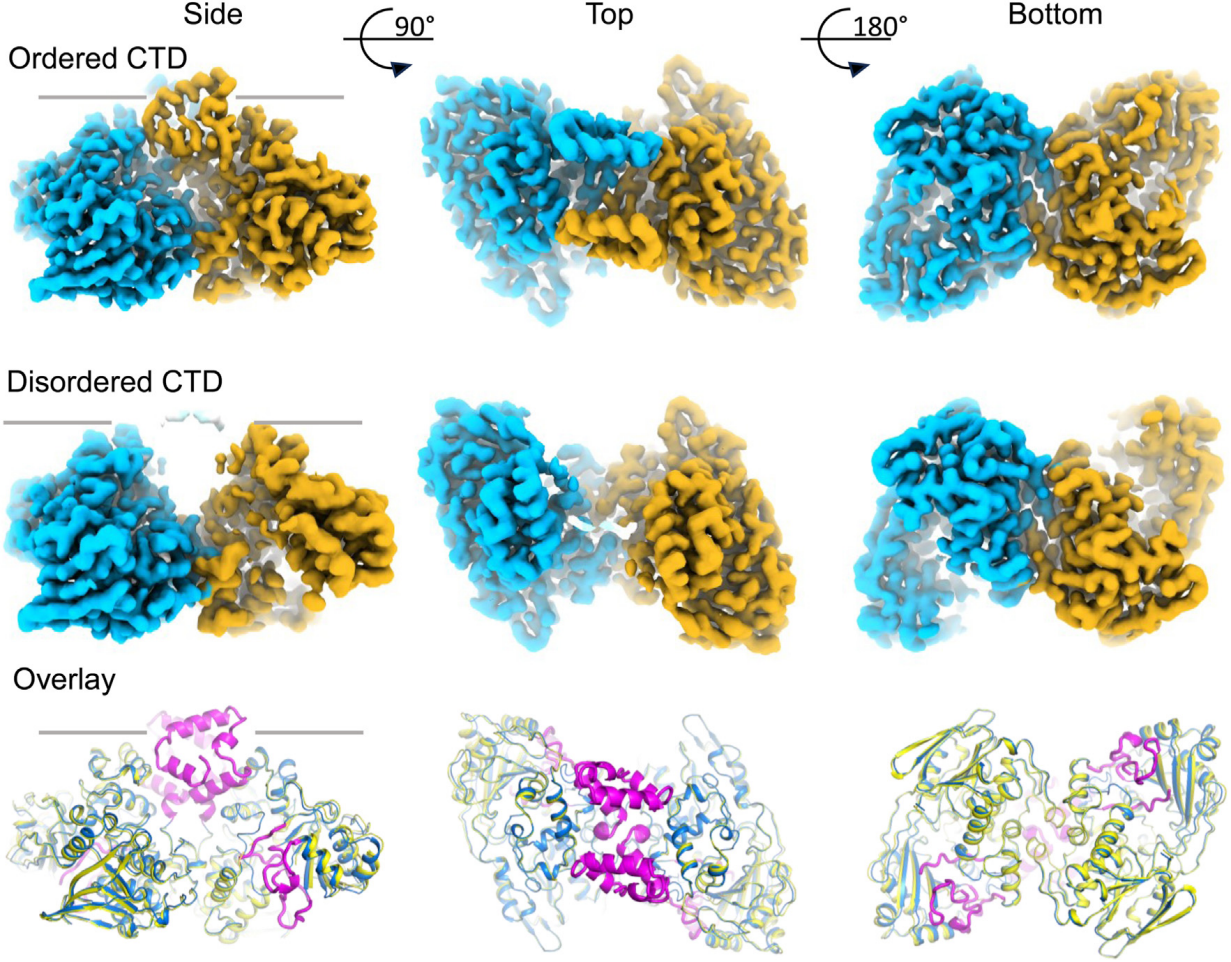
**3D variability analysis of the cryo-EM OhyA dimer shows variability of the CTD.** Comparisons of the dimer reconstructions for the ordered (*top row*) and disordered (*middle row*) CTD particle clusters. Ordered and disordered CTD particle clusters are from the same 3D variability job. For each, the side view (from the edge of the membrane inner leaflet denoted by a *black line*), the top view (from the plane of the membrane bilayer), and the bottom view (from the cytoplasm) are shown. The reconstructions are colored for each OhyA protomer. Structural overlays of the refined dimer structures for the ordered (*blue*) and disordered (*yellow*) CTD clusters. The regions of the ordered CTD dimer absent in the disordered CTD dimer (120–124, 208–222, 366–378, 394–406) are colored in *magenta*.

We observed multiple states of the OhyA carboxy-terminus in the 2D classes from the dimer dataset. 3D variability analyses can be used to detect conformational variability in single particle micrographs (29) so we performed 3D variability analysis to measure the dynamic behavior of the carboxy terminus in solution (Fig. 3). 572,989 particles were refined for variability analysis to ascertain the distribution of their dynamic molecular motion. 278,661 particles clustered within a cluster with the best density for the carboxy terminus, consistent with the crystallographic conformation. The reconstruction from the variability analysis of this state yielded a 2.79 Å map. A 3.03 Å map was determined from 40,922 particles that clustered with the worst density for the carboxy terminus. The remaining particles were in intermediate states presumably occupying the entire conformational space accessible to the carboxy terminus. The states primarily differed by the overall density of the carboxy terminus, but no discrete sub-class of molecular structure was observed. This analysis confirms the carboxy terminus is a flexible domain that can adopt multiple conformations, but there is no preferred conformation in the trajectory of the carboxy terminus in motion under these conditions.

### OhyA uses the carboxy terminus to bind lipids

**Figure 4:**
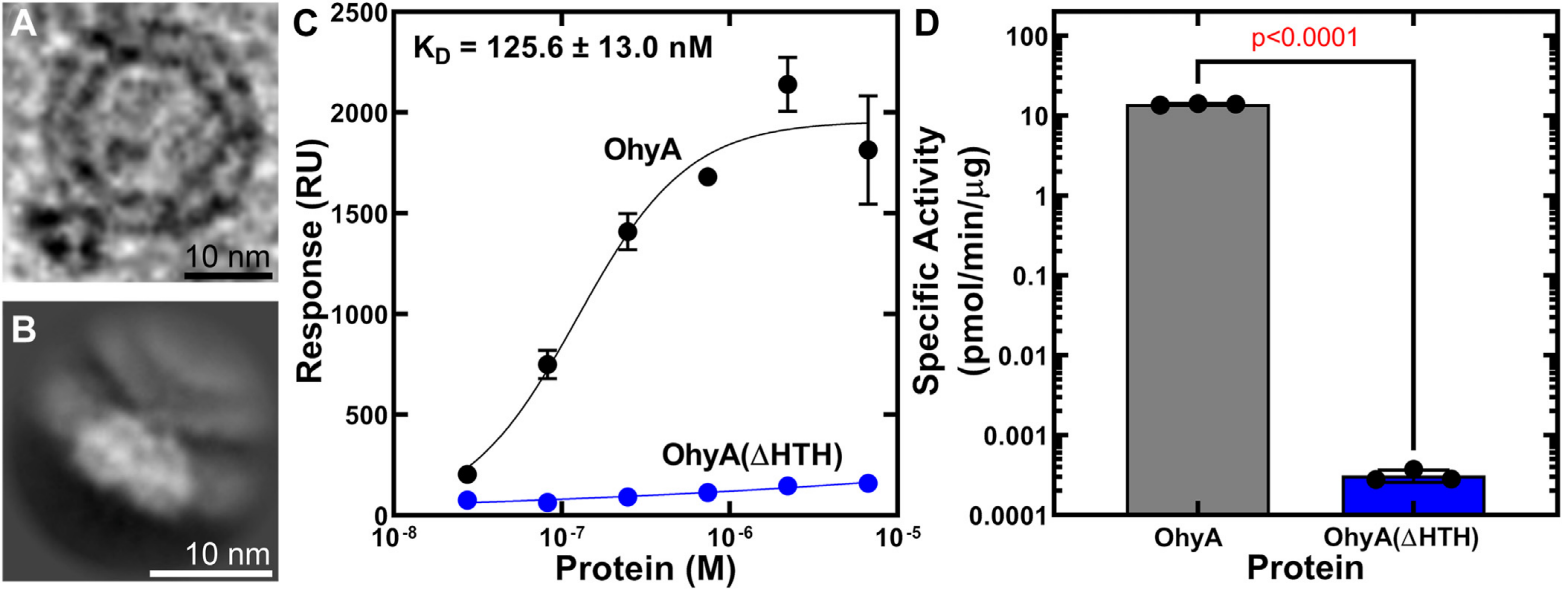
**Functional characterization of the OhyA carboxy terminus.***A*, cryo-electron microscopy (cryo-EM) particle patch from a micrograph of OhyA bound to a POPG:POPC liposome. The scale bar corresponds to 10 nm. *B*, cryo-EM 2D class average (representing 5374 particles) of OhyA association with POPG:POPC liposomes. The scale bar corresponds to 10 nm. *C*, surface plasmon resonance determination (n = 3 technical replicates) of DOPG/DOPC liposome binding affinity of OhyA (*black*) and OhyA(ΔHTH) (*blue*). The data from three independent titrations were fit to the Hill equation to calculate the K_D_. Mean ± S.D. *D*, specific activities (n = 3 technical replicates) of OhyA (13.887 ± 0.343 pmol/min/μg) and OhyA(ΔHTH) (0.0003 ± 0.00005 pmol/min/μg). Unpaired *t* test determined if differences between OhyA and OhyA(ΔHTH) specific activities have statistical significance and determined the two-tailed *p* value. Mean ± S.D.

Phosphatidylglycerol (PG) is the dominant phospholipid in *S. aureus* membranes, and we prepared PG liposomes for OhyA-lipid binding studies. We incubated OhyA with prepared POPG: POPC liposomes for cryo-EM analysis. OhyA-bound liposomes were evident in particle patches and resultant 2D class averages from the cryo-EM dataset (Fig. 4, *A* and *B*) showing OhyA associates with the liposome surface. OhyA has an asymmetrical fold (Fig. 1, *B* and *C*) that suggests OhyA is oriented with the carboxy terminus toward the liposome surface. This interpretation is consistent with the electrostatic complementarity of the positively charged OhyA carboxy terminus (Fig. 1*C*) with anionic PG phospholipid.

We validated this observation using surface plasmon resonance to measure direct binding. We prepared small unilamellar phospholipid vesicles from dioleoyl-PG (DOPG) and dioleoyl-phosphatidylcholine (DOPC), and 50:50 DOPG:DOPC small unilamellar phospholipid vesicles were immobilized on an L1 chip. Small unilamellar phospholipid vesicles are captured on the L1 chip through hydrophobic interactions with the hydrogel layer on the chip and DOPC was used to stabilize immobilization on the L1 chip because higher levels of DOPG yielded unstable surfaces. This may be caused by the carboxymethyl dextran-based hydrogel with a net negative charge that may cause electrostatic repulsion of lipids with negatively charged headgroups like DOPG. Increasing the concentration of OhyA from 27.4 nM to 6.7 μM, we determined an equilibrium dissociation constant (K_D_) of 125.6 ± 13.0 nM (Fig. 4*C*). This binding interaction confirms OhyA directly interacts with phospholipids. When we repeated the surface plasmon resonance experiment using OhyA(ΔHTH), negligible membrane lipid binding was observed (Fig. 4*C*) indicating that the carboxy terminus is necessary for membrane binding.

We assayed OhyA and OhyA(ΔHTH) for biochemical-specific activity using 50:50 DOPC:DOPG small unilamellar phospholipid vesicles to deliver unsaturated fatty acid OhyA substrate and measured conversion to *h*FA. The specific activity of OhyA(ΔHTH) was 4.6 log-fold lower than OhyA (Fig. 4*D*). These data indicate the carboxy terminus is required to bind membrane lipids and obtain fatty acid substrate.

### OhyA carboxy terminus has intrinsic membrane binding properties

**Figure 5:**
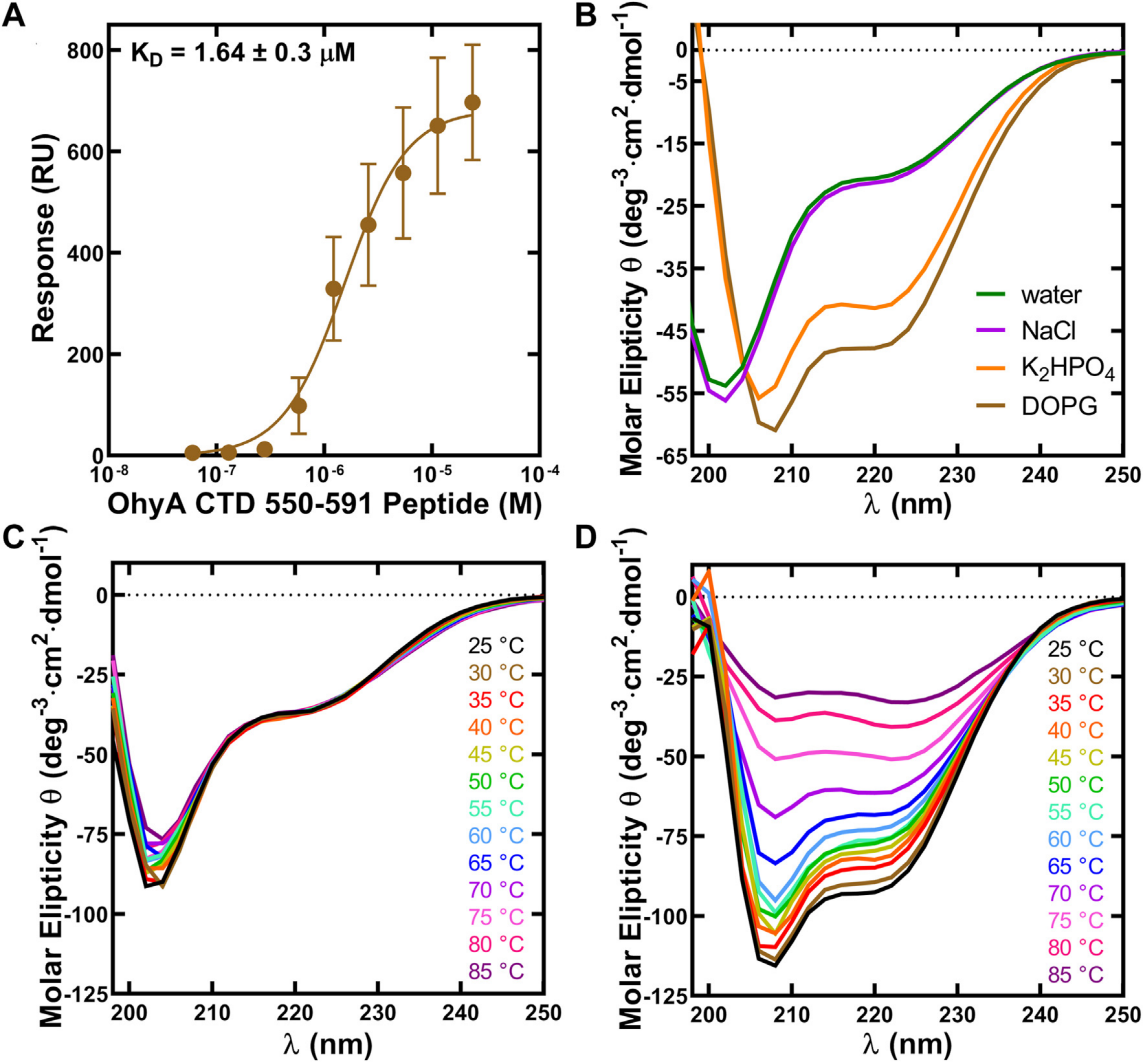
**Lipid binding and circular dichroism (CD) spectroscopy of OhyA CTD peptide(550–591).***A*, surface plasmon resonance determination (n = 3) of DOPG/DOPC liposome binding affinity of OhyA CTD peptide(550–591). The data from three independent titrations were fit to the Hill equation to calculate the K_D_. Mean ± S.D. *B*, CD spectra of OhyA CTD peptide(550–591) at 25 °C mixed with water (*green*), 150 mM NaCl (*purple*), 50 mM K_2_HPO_4_ (*orange*), or 2 mM dioleoyl-PG (DOPG) liposomes (*brown*). *C*, CD spectra of OhyA CTD peptide(550–591) in water collected along a thermal gradient. *D*, CD spectra of OhyA CTD peptide(550–591) in 2 mM DOPG liposomes along a thermal gradient.

We synthesized a peptide of residues 550 to 591 from the OhyA carboxy terminus (OhyA CTD peptide(550–591)) (Fig. S5) to study lipid binding by this fragment of the protein (see Experimental procedures). We used surface plasmon resonance to measure OhyA CTD peptide(550–591) association with 50:50 DOPG:DOPC small unilamellar phospholipid vesicles and determined a K_D_ of 1.64 ± 0.3 μM to (Fig. 5*A*). This binding interaction shows the carboxy terminus polypeptide sequence has an intrinsic membrane association. The ∼log-fold reduction in lipid-binding K_D_ of OhyA CTD peptide(550–591) compared to OhyA indicates that the carboxy terminus is a multivalent lipid binding site where the lipid-binding contribution from each carboxy terminus is additive, and not fully captured by using a monomeric peptide. Furthermore, additional membrane interactions could be occurring on other sites of the protein, or the conformation for membrane interaction is partially satisfied by the monomeric peptide.

We used CD spectroscopy to study the effect of phospholipids on the secondary structure of OhyA CTD peptide(550–591) as a function of coupling structure formation to partitioning (30). The CD spectral profile of OhyA CTD peptide(550–591) in water shows low or moderate helicity in solution with two wavelength minima at ∼205 nm and ∼222 nm (Fig. 5*B*) that correspond to a mixture of α-helix and random coil, and show the peptide is not in a completely unfolded state in water. The CD spectrum did not change as the temperature is increased indicating that this structure is resistant to thermal denaturation (Fig. 5*C*). Next, we tested the effects of surface plasmon resonance binding buffer components (see Experimental procedures) on OhyA CTD peptide(550–591) structure (Fig. 5*B*). There was negligible change to the OhyA CTD peptide(550–591) profile from water to 150 mM sodium chloride (NaCl). Significant peak shifts were observed when OhyA CTD peptide(550–591) was exposed to 50 mM potassium phosphate (K_2_HPO_4_) compared to water (Fig. 5*B*). While both wavelength minima increase (become more negative) in the K_2_HPO_4_ condition, the minimum increase at ∼222 nm exhibits a striking change that is consistent with an increase in helical content. These shifts are consistent with a backbone conformation change because CD signals in the far ultraviolet wavelength region (<240 nm) are influenced by the amide chromophores of peptide bonds (31). Both wavelength minima occurring at ∼205 nm and ∼222 nm increase when OhyA CTD peptide(550–591) is exposed to DOPG in surface plasmon resonance running buffer compared to K_2_HPO_4_ only (Fig. 5*B*). The CD spectral profile of OhyA CTD peptide(550–591) in water as a function of temperature shows the wavelength minima at ∼205 nm and ∼222 nm and the profile does not change by increasing temperature (Fig. 5*C*). When the peptide is mixed with DOPG liposomes, the wavelength minima at ∼205 nm and ∼222 nm decrease as temperature increases (Fig. 5*D*), but the profile does not convert to the peptide in water profile. Instead, OhyA CTD peptide(550–591) seems to be denaturing as it is likely to be in a more complete helical conformation. These data suggest the OhyA carboxy terminus interacts with the phosphate layer of membrane lipids.

### MD simulation predicts residues in helix α19 participate in membrane association

**Figure 6:**
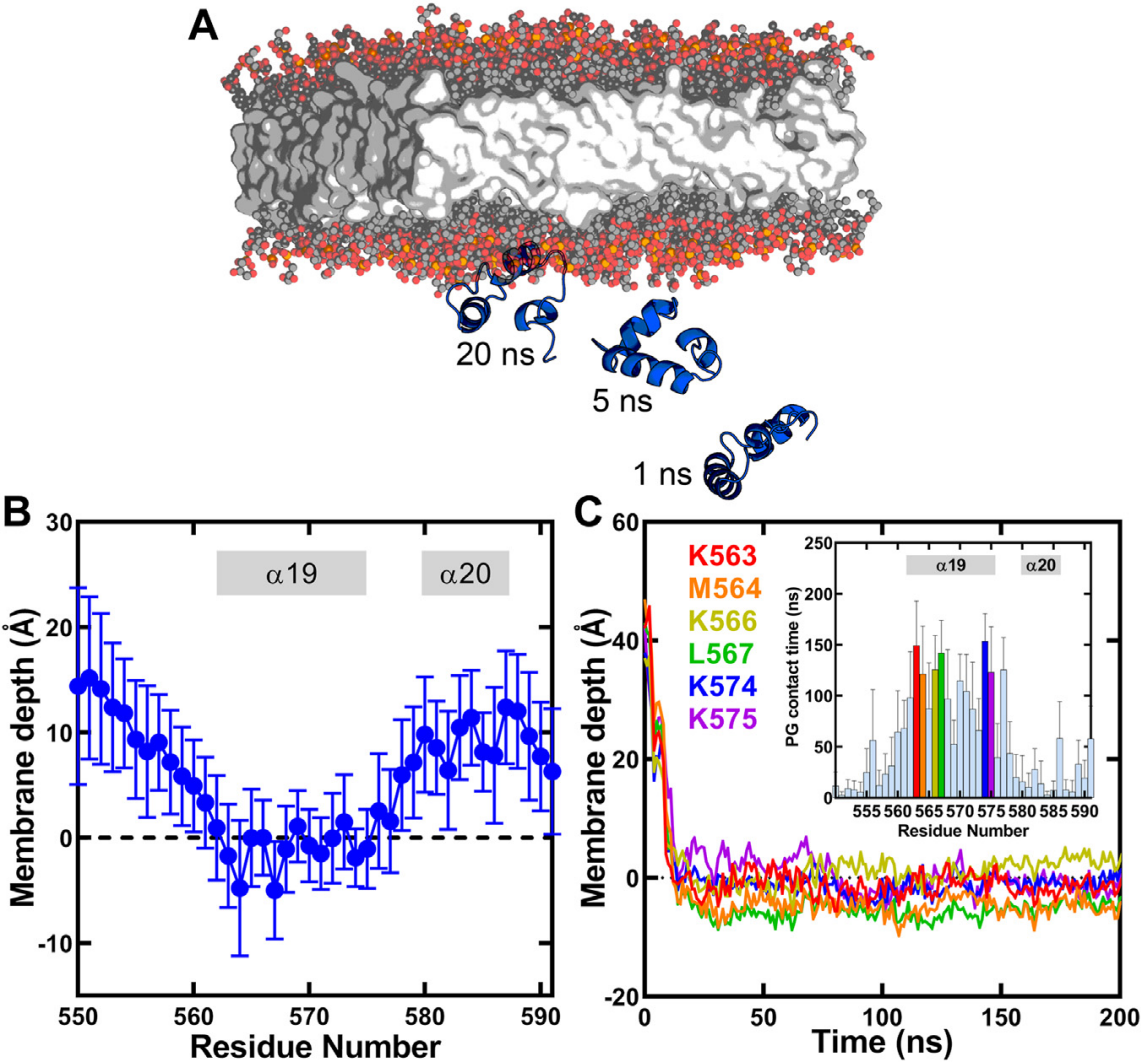
**Molecular dynamics simulations of OhyA CTD peptide(550–591) binding a phosphatidylglycerol (PG) bilayer.***A*, snapshots from the simulation of OhyA CTD peptide(550–591) binding a PG highly mobile membrane mimetic (HMMM) model. *B*, ensemble-averaged closest distance for each amino acid residue (heavy atoms only) to the membrane *cis* phosphorus plane along the membrane normal (z) axis calculated over multiple HMMM simulations using the membrane-bound frames (see Experimental procedures) (n = 22). Annotations indicate residue positions that correspond to helices α19 and α20. The black dotted line at *y* = 0 represents the average position of the phosphorous atoms in the *cis* leaflet. Residues in helix α19 bound the phosphate plane of the membrane. Mean ± S.D. *C*, representative closest distance between the top 6 residues (heavy atoms only) predicted to interact with the membrane to the phosphate plane along the membrane normal (z) axis, plotted during the entire 200 ns HMMM binding simulation from replica x105. *Inset*, ensemble-averaged contact analysis between each residue and PG lipids over multiple HMMM simulations using the membrane-bound frames (see Experimental procedures) (n = 22). Contact between a residue and PG is defined when a residue heavy atom is within 3.5 Å of a PG heavy atom.

We deployed an MD simulation pipeline to describe the binding of OhyA CTD peptide(550–591) to a negatively charged PG surface and to examine whether the amphipathic peptide can be used as a model to study OhyA-membrane binding. For the initial setup, we started with the structured OhyA carboxy terminus extracted from the published crystallographic conformation (1). A monomeric peptide was placed above a PG highly mobile membrane mimetic (HMMM) model (32), which includes an organic solvent (Simple Carbon Solvent Ethane, SCSE for the simulations reported here) designed to mimic the membrane interior (33) (Fig. 6*A*). Portions of the peptide that could possibly associate with the membrane were identified by multiple 200 ns simulations, starting from the peptide rotated in randomized orientations (each rotated in increments of 15 degrees around a principal axis to vary the starting point) (Figs. 6*B* and S6). Peptide-membrane association occurred rapidly and we observed spontaneous membrane binding regardless of the peptide’s starting point (Fig. S7). In 22 out of 24 simulations (∼92%), the final, membrane-bound orientations of the peptide were identical, indicative of a preferred membrane-binding orientation (Fig. S6). The contact analysis between the peptide and lipids (Fig. S8), and the time-averaged distance of each residue to the membrane phosphate plane (Fig. 6*B* and S6) indicated that helix α19, which is also the longer helix in the HTH, is the most responsible element for membrane binding (Fig. 6*B*). Amino acids along the helix α19 (between S562 and K575) maintain continuous association with PG during the simulation (Figs. 6*C* and S9) and anchor the peptide to the membrane through both electrostatic and hydrophobic interactions (Fig. S10). The amino terminus of the peptide interacts with the membrane only transiently in some simulations, and the helix α20 displayed a minor contribution to membrane association (Fig. S9). The MD simulations posit a model where key lysine residues along the amphipathic helix α19 are poised to interact with the phosphate layer through electrostatic interactions, and M564 and L567 facilitate hydrophobic interactions with lipid tails when the carboxy terminus is not conformationally constrained by the rest of the OhyA protein (Fig. S10).

### NMR spectroscopy identifies a flexible hinge region in the carboxy terminus

**Figure 7:**
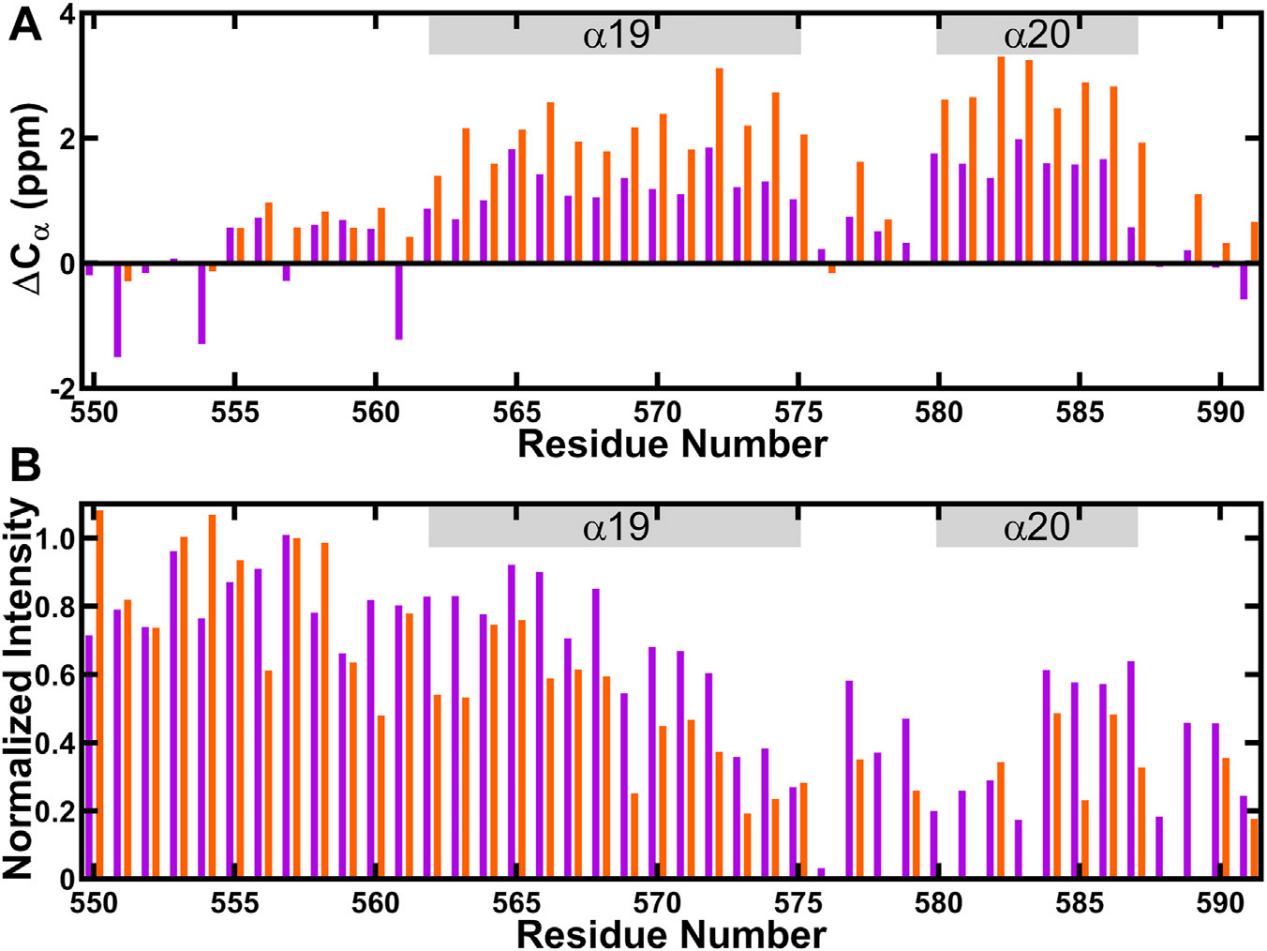
**Nuclear magnetic resonance (NMR) spectroscopy of OhyA CTD peptide(550–591).***A*, deviation in Cα chemical shift plotted as a function of residue number for OhyA CTD peptide(550–591) mixed with 150 mM NaCl (*purple*) or 50 mM K_2_HPO_4_ (*orange*). Annotations indicate residue positions that correspond to helices α19 and α20. *B*, plot of normalized intensity of peaks in 2D[^15^N,^1^H] spectra in 150 mM NaCl (*purple*) or 50 mM K_2_HPO_4_ (*orange*).

**Figure 8:**
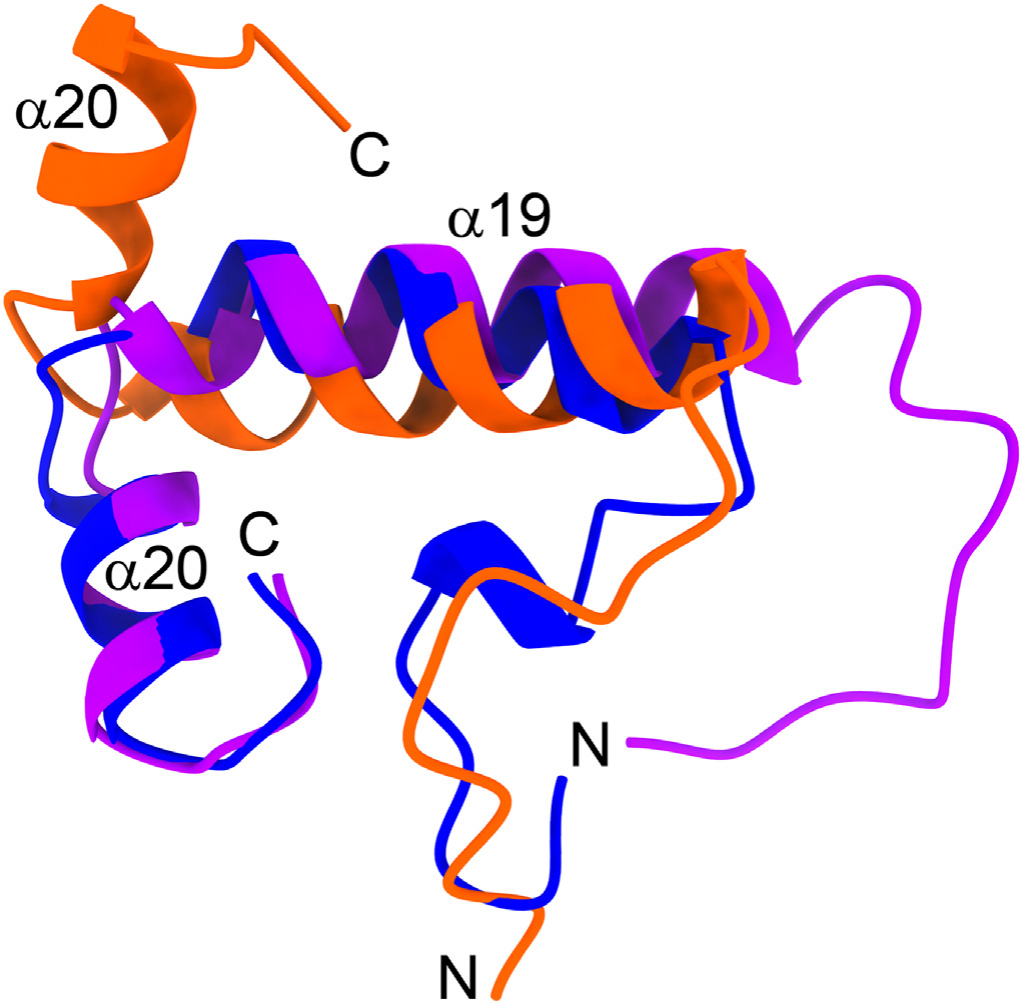
**NMR structures of OhyA CTD peptide(550–591).** Lowest energy states of the NMR structures of OhyA CTD peptide(550–591) in 150 mM NaCl (*purple*) or 50 mM K_2_HPO_4_ (*orange*) superimposed with the CTD region (550–591) from the OhyA crystal structure (PDB ID: 7KAV) (*blue*). The locations of helices α19 and α20, and the amino (N) and carboxy (C) termini are indicated for each structure.

We decided to probe the conformation of OhyA CTD peptide(550–591) in solution using NMR spectroscopy. Two-dimensional (2D) [^1^H, ^1^H]-based COSY, TOCSY, and NOESY experiments and natural abundance 2D [^15^N, ^1^H] and 2D [^13^C, ^1^H] HSQC spectra were used for this purpose. In 150 mM NaCl, though the peptide showed poor chemical shifts dispersion (Fig. S11*A*), analyses of the Cα resonances indicate that the peptide adopts a helical conformation from 560 to 591 with two helical regions from 562 to 575 and 580 to 587, while 550 to 559 is disordered (Fig. 7*A*). All the backbone amide resonances were observed in 2D [^15^N, ^1^H] HSQC spectra, except I576 (Fig. S12*A*). The intensities for the residues in the carboxy terminus of the peptide are lower than the residues in the amino terminus suggesting the amino terminus is highly flexible in solution (Fig. 7*B*). We determined the structure of the peptide in NaCl (Table 2 and Fig. 8) and, as predicted by the Cα-chemical shifts, the peptide adopts a helical conformation. For clarity, we number the amino acids and secondary structure components of OhyA CTD peptide(550–591) according to their corresponding elements in the full-length OhyA structure. In the NMR structure, residues 562 to 575 compose helix α19, and residues 580 to 587 make up helix α20. Independent superimposition of the two α-helices onto the crystallographic carboxy terminus (RMSD is <1 Å) shows the peptide helices are in identical conformations as α19 and α20 in the crystal structure and the hinge region connecting the two helices is flexible (Fig. 8). The line broadening or lower intensities observed for residues 576 to 579 in the 2D [^15^N-^1^H] HSQC spectrum is consistent with a flexibility hinge in this region (Fig. S12*A*). These structural features indicate that the peptide is a reasonable model to study the carboxy terminus of OhyA.

**Table 2:** NMR Structural statistics of OhyA CTD peptide(550–591)

OhyA CTD peptide(550–591) sample	NaCl	K_2_HPO_4_
BMRB ID	31,112	31,113
PDB ID	8UM1	8UM2
Restraints		
NOE (Total)	478	547
Intra-residue	213	222
Short(|i-j| < 1)	130	146
Medium range (2 < |i-j| < 5)	115	151
Long range (|i-j| > 5)	20	28
NOE restraints/residue	15	18
Hydrogen bond	40	36
Dihedral angle	64	66
Residual distance restraint violations		
Average distance violation (Å)	0.006 ± 0.002	0.004 ± 0.001
Maximum distance violation (Å)	0.14 ± 0.02	0.15 ± 0.03
Average angle violation (^o^)	0.14 ± 0.04	0.49 ± 0.02
Maximum angle violation (^o^)	0.17 ± 0.04	0.52 ± 0.08
Model Quality		
RMSD from average coordinates (Å)		
All Backbone atoms (560–591)	0.24 ± 0.08	0.28 ± 0.09
All Heavy atoms (560–591)	0.72 ± 0.10	0.65 ± 0.13
RMSD Bond lengths (Å)	0.02	0.02
RMSD Bond angles (^o^)	1.1	1.1
Molprobity Ramachandran plot		
Most favored regions (%)	96.9	98.5
Additionally allowed regions (%)	2.4	1.5
Disallowed regions (%)	0.6	0.0
MolProbity clashscore (raw/Z)	8.48/0.07	7.34/0.27

In 50 mM K_2_HPO_4_ the peptide showed large dispersion in the chemical shifts (Fig. S11*B*), and we determined the structure of the peptide in K_2_HPO_4_ (Table 2 and Fig. 8). The deviation in the Cα-chemical shift for the peptide in K_2_HPO_4_ showed higher percentage of helicity than in NaCl (Fig. 7*A*). Several peaks in the carboxy terminus of the peptide either could not be observed or showed weak intensity in the 2D [^15^N, ^1^H] HSQC spectrum (Figs. 7*B* and S12*B*), suggesting the carboxy terminus is undergoing intermediate exchange under this condition. Residues 550 to 559 are disordered and the helical region of the peptide has the same conformation as the carboxy terminus of crystal structure (RMSD is <1 Å) (Fig. 8). G578 in the hinge region was missing in the 2D [^15^N, ^1^H] HSQC spectrum (Fig. S12*B*) and the amide proton showed a broad resonance at 9.18 ppm (NOESY spectrum, Fig. S12*C*), indicating its involvement in a hydrogen bond with the side chain of E582 which is observed in the crystal structure. The aromatic side chain of F572 is positioned to act as a hub to hold the two ordered helices together showing the maximum number of inter-residue NOEs. The data suggest that the peptide interacts with the negatively charged PO_4_ ions in K_2_HPO_4_. There is negligible change in the chemical shifts in 1% glycerol (Fig. S11*C*), indicating that the peptide does not interact with glycerol.

### NMR spectroscopy shows sequential helix engagement drives peptide-membrane interaction

**Figure 9:**
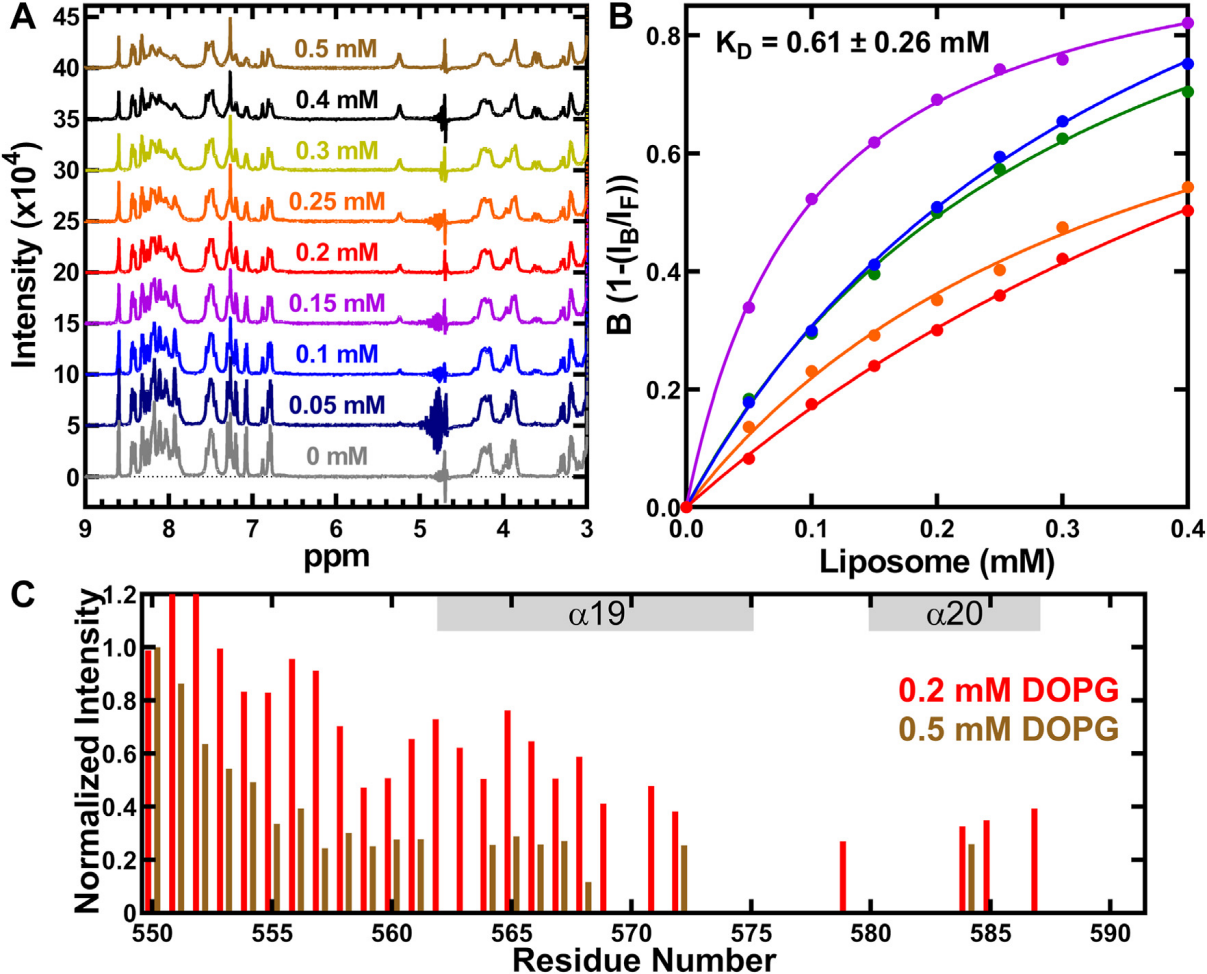
**OhyA CTD peptide(550–591) interacts with DOPG liposomes.***A*, 1D ^1^H-spectra of 1 mM OhyA CTD peptide(550–591) in 150 mM NaCl, with increasing concentration of DOPG liposomes as indicated. The line broadening of resonances indicate OhyA CTD peptide(550–591) binds DOPG liposome. *B*, line broadening of resonances quantified as B (34), plotted as a function of concentration. B is an intensity ratio derived from one-(I_B_/I_F_) where I_B_ and I_F_ are the intensities of the peaks from the peptide in liposome-bound and liposome-free forms respectively. Dots represent experimental values and lines the fitted equation to derive the K_D_ values. *C*, quantitative analysis of the normalized intensity of the peaks from (*A*) and Fig. S13 plotted as a function of the residue number. The same coloring scheme in the spectra is used.

We titrated 0.05 to 0.5 mM DOPG liposome into the NMR sample containing 1 mM peptide and 150 mM NaCl and recorded the 1D ^1^H-NMR spectra to identify the region of the peptide that interacts with the membrane bilayer. We observed line broadening and decrease in intensity of the OhyA CTD peptide(550–591) peaks in the 1D ^1^H NMR spectra indicating peptide-liposome binding (Fig. 9*A*). We quantified the decrease in peak intensity as a function of liposome concentration (34) and obtained an equilibrium dissociation constant (K_D_) of 0.61 ± 0.26 mM (Fig. 9*B*). We recorded natural abundance 2D [^15^N, ^1^H] HSQC spectra for peptide mixed with 0.2 mM and 0.5 mM DOPG liposome (Fig. S13, *A* and *B*) and at 0.5 mM DOPG liposome, most of the residues beyond A570 were not observed in the spectrum because of line broadening indicative of the binding of the peptide with the liposome (Fig. 9*C*). At 0.5 mM DOPG liposome, we observed NOEs from the DOPG liposome resonances (clearly at 5.27 ppm, next to the phosphate group) to the peptide in the 2D [^1^H,^1^H] NOESY spectra (Fig. S13, *C* and *D*). These data show that the peptide directly interacts with the DOPG liposome.

**Figure 10:**
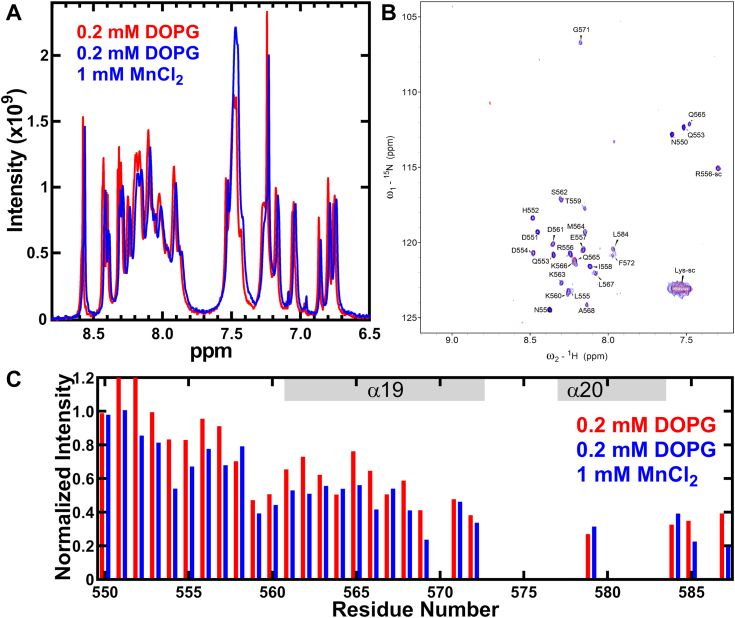
**OhyA CTD peptide(550–591) interacts with the membrane surface.***A*, 1D NMR spectra showing the interaction with 0.5 mM MnCl_2_ observed as line broadening of OhyA CTD peptide(550–591) in 0.2 mM DOPG liposome resonances. *B*, 2D [^15^N, ^1^H] HSQC spectra of OhyA CTD peptide(550–591) with 0.2 mM DOPG liposome (*red*) ± 0.5 mM MnCl_2_ (*blue*). In the presence of MnCl_2_, decrease in intensity was observed for several peaks suggesting that the peptide interacts with the surface of the liposome. *C*, quantitative analysis of the normalized intensity of the peaks from (*B*) is plotted as a function of the residue number. The same coloring scheme in the spectra is used.

We added MnCl_2_ to the NMR sample of the peptide-containing peptide and 0.2 mM liposome to probe whether the peptide associates with the surface or penetrates the liposome (35), and measured the 2D [^15^N, ^1^H] HSQC spectrum. Adding MnCl_2_ decreased the intensities of several peaks in the peptide (Fig. 10), which indicates that the peptide associates with the surface of the DOPG liposome rather than inserted into the membrane bilayer. Mn ions bind surface phosphate groups of the liposome and cause line broadening of liposome resonances but does not associate with the acyl chain (36). Therefore, paramagnetic Mn can distinguish between modalities of membrane association and show the carboxy terminus peptide binds to the DOPG liposome surface.

### MD simulation of OhyA dimer binding full membrane (FM) lipids

**Figure 11:**
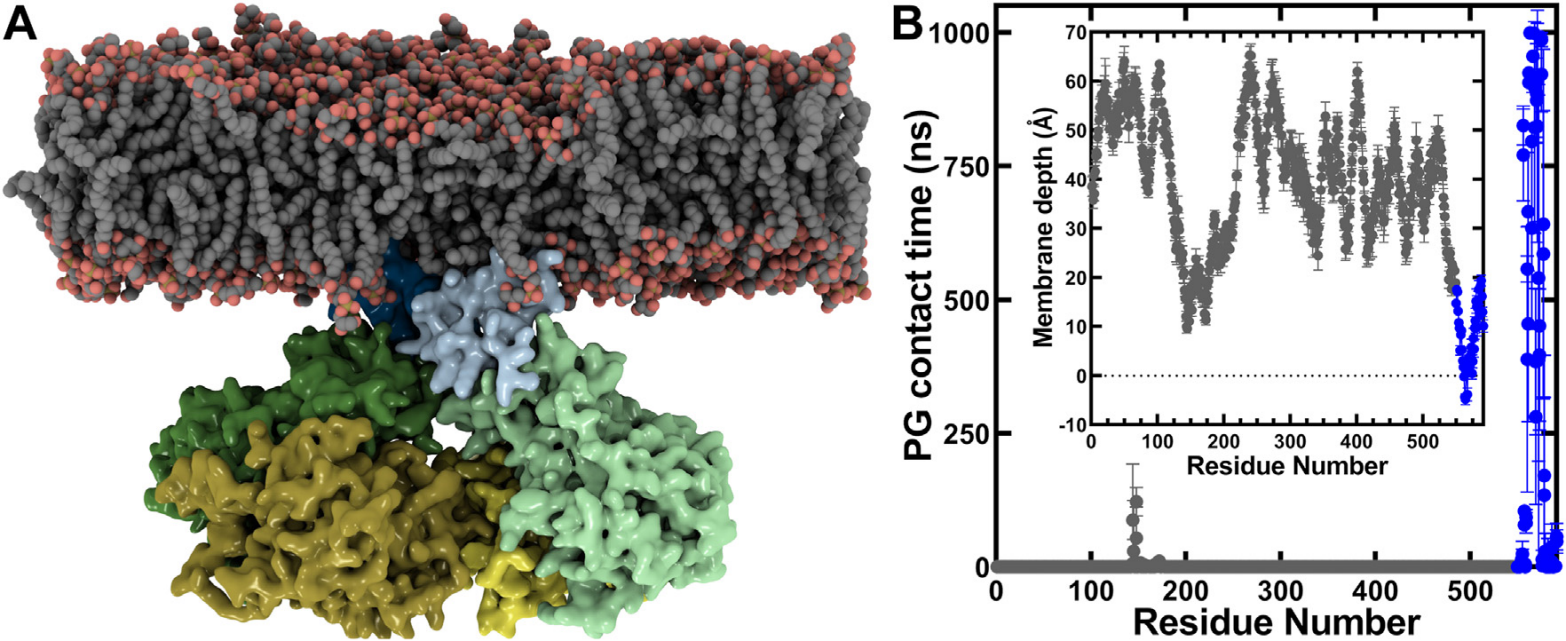
**MD simulations of OhyA dimer bound to a DOPG membrane bilayer.***A*, snapshot from the simulation of OhyA dimer bound to a DOPG full membrane. OhyA is colored by functional domain as shown in Fig. 1*B*. van der Waals representation of the DOPG membrane shows carbon in *grey*, oxygen in *red*, and phosphorus in *tan*. *B*, ensemble-averaged contact analysis between each amino acid residue and the full membrane from the 1 μs membrane-bound OhyA dimer simulations (n = 2). *Inset*, ensemble-averaged closest distance of each residue (heavy atoms only) to the membrane phosphate layer along the membrane normal (z) axis calculated from the 1 μs full membrane-bound OhyA dimer simulations. Mean ± S.D. The black dotted line at *y* = 0 represents the average position of the phosphorous atoms in the membrane leaflet that are within 6 Å of the membrane-bound OhyA dimer. Residues from each protomer were calculated independently, and the simulations were done twice. The residues in the carboxy terminus are *blue*, and all other residues are *dark grey*.

**Figure 12:**
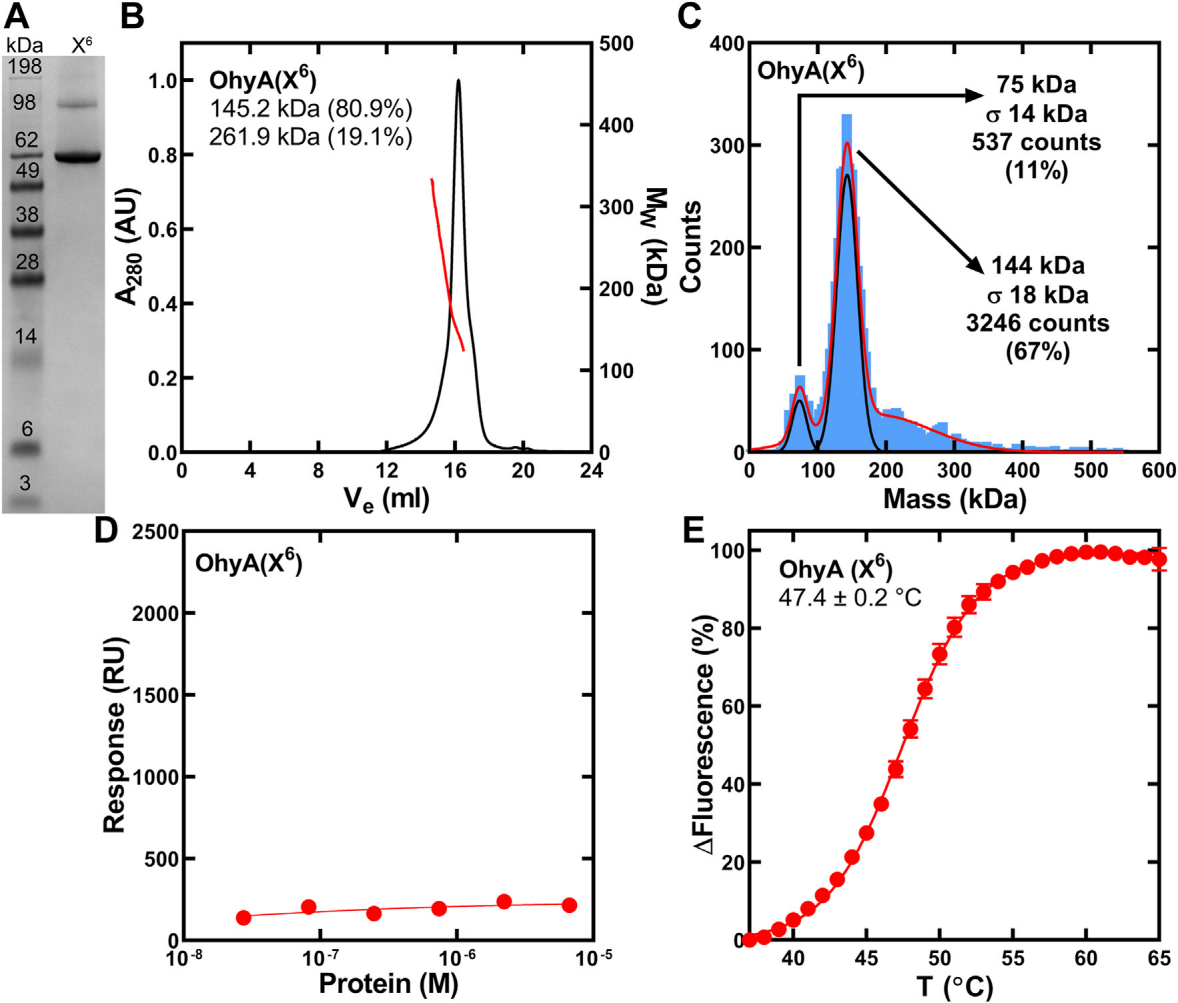
**Preparation and characterization of OhyA(X**^**6**^**).***A*, OhyA(X^6^) was a 65-kDa monomer and was >95% pure based on denaturing gel electrophoresis. *B*, His-tagged OhyA(X^6^) was purified by Ni^2+^-affinity chromatography followed by gel filtration chromatography on Superose 6 Increase 10/300 Gl and multi-angle light scattering (SEC-MALS). The molar mass distribution (*red line*) is superimposed on a chromatogram of A_280_*versus* elution volume (*black line*). SEC-MALS measured OhyA predominantly migrates as a 145-kDa dimer. *C*, mass photometry measured OhyA remains a 144-kDa dimer in dilute concentrations. *D*, surface plasmon resonance determination (n = 3 technical replicates) of DOPG/DOPC liposome binding affinity of OhyA(X^6^). The data from three independent titrations were fit to the Hill equation. Mean ± S.D. *E*, protein thermal denaturation analysis was used to determine the structural integrity of OhyA(X^6^). Assays (n = 5 technical replicates) contained 1 mg/ml OhyA(X^6^). The data are fit to the Boltzmann equation. Mean ± S.D.

Initial efforts to superimpose full-length OhyA onto the membrane-bound conformation of OhyA CTD peptide(550–591), as captured in the HMMM simulations, were unsuccessful due to the incompatibility of the crystallographic conformation with binding the membrane surface (Fig. S14). The crystallographic conformation of the carboxy terminus causes one protomer to clash with the membrane while the other protomer binds the membrane (Fig. S14). NMR analysis of the carboxy terminus peptide and cryo-EM analysis of the carboxy terminus fused to the whole protein are in agreement that the carboxy terminus is a flexible domain. However, the flexibility observed in NMR arises from the additional degree of freedom originating from the absence of the amino-terminal domains, and the cryo-EM showed a multitude of microstates of the carboxy terminus but did not yield the structures of any alternative conformations. Therefore, we performed a 1.1 μs whole-protein water box simulation to examine if we could capture a conformation of the carboxy terminus in the context of the whole protein that would enable both protomers to bind the PG surface. The carboxy termini of both protomers reached a root-mean-square fluctuation value of ∼6 Å, and the amphipathic helices sampled various orientations, with few resulting in helix α19 adopting a more horizontal orientation similar to its conformation in the membrane-bound OhyA CTD peptide(550–591) model (Fig. S15). This arrangement enabled the successful superimposition of full-length OhyA onto the membrane-bound OhyA CTD peptide(550–591) (Figs. S15 and S16) and the generation of the OhyA•FM model (Fig. 11*A*). The OhyA CTDs remain associated with the membrane throughout the entire simulation (Fig. 11*B*), which validates the stability of the OhyA membrane-bound model. We observed minor contact between membrane lipids and the fatty acid lobe when OhyA tilts in the simulation because of membrane interactions with residues that are adjacent to the CTDs. The MD simulations showed the same residues (K563, M564, K566, L567, K574, and K575) drive OhyA•PG interactions as OhyA CTD peptide(550–591)•PG binding interactions (Figs. 11*B*, S8, and S17). A difference in the simulations using the peptide (Fig. S8) *versus* the whole protein is that, in the context of the whole protein, helix α-20 was shielded by the dimer thus blocking its interaction with the membrane (Fig. S17). Even so, the same residues were implicated in membrane binding. We introduced six point mutations (K563E, M564A, K566E, L567A, K574E, and K575E) to make OhyA(X^6^) and eliminate the key basic/hydrophobic residues that are predicted by MD simulation to engage PG (Fig. S17). OhyA(X^6^) purifies as a dimeric protein (Fig. 12, *A–C*). We performed surface plasmon resonance with OhyA(X^6^) and 50:50 DOPG:DOPC small unilamellar phospholipid vesicles, and measured negligible membrane lipid binding (Fig. 12*D*). The thermal stability of OhyA(X^6^) (Fig. 12*E*) is comparable to OhyA (Fig. 2*G*) indicating the mutagenesis did not compromise OhyA(X^6^) folding. Therefore, these data validate the significance of the hotspots predicted by our MD simulations in the context of OhyA membrane binding.

### OhyA requires the carboxy terminus for function *in vivo*

**Figure 13:**
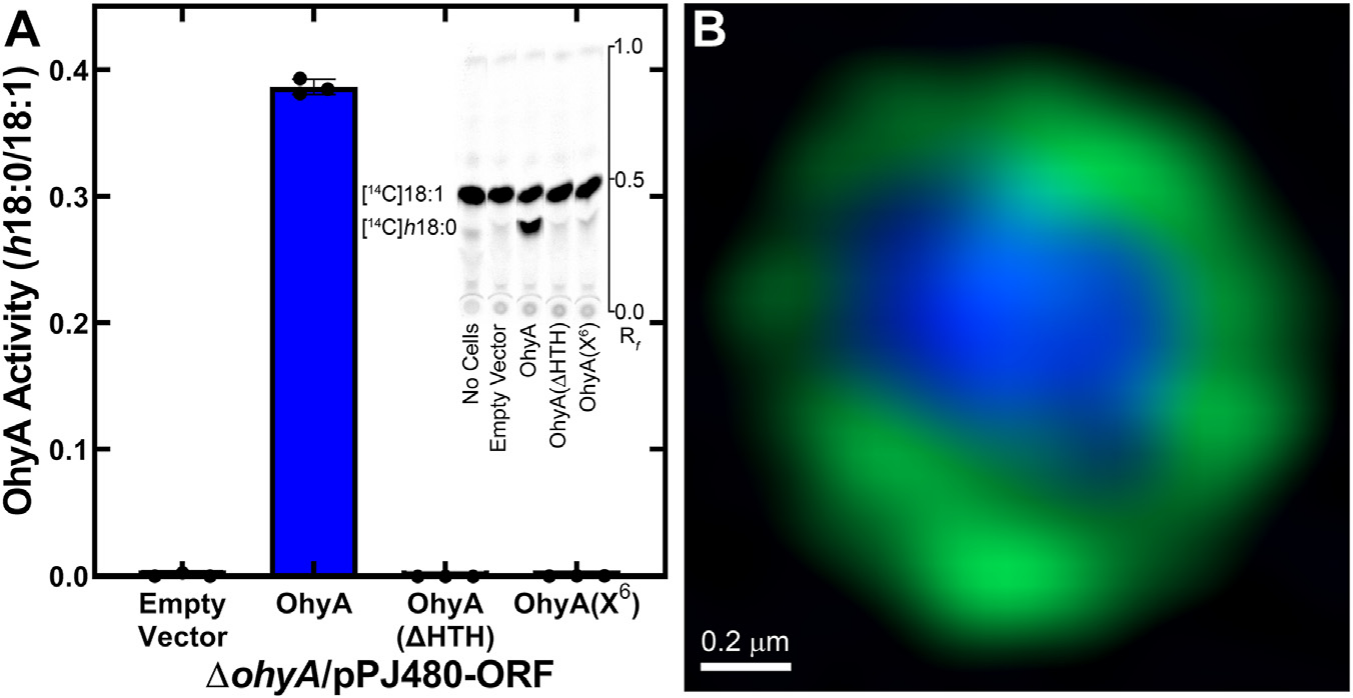
**Cellular characterization of the OhyA carboxy terminus.***A*, ratio of [1-^14^C]*h*18:0 to [1-^14^C]18:1 in culture supernatants from *Staphylococcus aureus* USA300 Δ*ohyA* cells harboring pPJ480 expression plasmids. Plasmid-bearing Δ*ohyA* cells expressed nothing (empty vector), OhyA, OhyA(ΔHTH), or OhyA(X^6^) and incubated with [1-^14^C]18:1 for 4 h. The culture supernatant was separated by thin layer chromatography, and quantified by phosphor imaging. N = 3 biological replicates, mean ± S.D. *Inset*, Representative thin layer plate of culture supernatants. *B*, super resolution AiryScan image of *S. aureus* RN4220 strain CDR002 expressing GFP-CTD, counterstained with DAPI (two merged channels). GFP-CTD is localized along the cellular boundary. The scale bar corresponds to 0.2 μm. The Fig. S19 contains the individual channels of this cell (Fig. S19*B*) and other representative CDR002 cells (Fig. S19, *A* and *C*).

We transformed plasmids containing nothing, OhyA, OhyA(ΔHTH), or OhyA(X^6^) into an *S. aureus* Δ*ohyA* knockout strain (6, 7) to compare their biochemical activities in a cell-based assay. The Δ*ohyA* knockout strain biochemical phenotype is the inability to convert unsaturated fatty acid to *h*FA, and Δ*ohyA* cells transformed with the empty plasmid do not produce *h*FA (Fig. 13*A*). The Δ*ohyA* cells harboring pOhyA convert oleic acid (18:1) to 10-hydroxystearic acid (*h*18:0), but Δ*ohyA* cells harboring pOhyA(ΔHTH) or pOhyA(X^6^) do not convert 18:1 to *h*18:0. We generated an anti-OhyA polyclonal antibody and probed the cellular lysates for protein production (Fig. S18). All strains produced equivalent amounts of protein indicating that failure of OhyA(ΔHTH) or OhyA(X^6^) to complement the Δ*ohyA* phenotype was due to inactive protein and not inequivalent gene expression. These data show that the carboxy terminus is essential for OhyA function *in vivo*, and the key residues predicted by MD to drive membrane association are required.

### OhyA carboxy terminus is sufficient to recruit heterologous protein to the cell membrane

We engineered a *S. aureus* strain that expresses chimeric green fluorescent protein fused to the OhyA CTD peptide(550–591) sequence (GFP-CTD) by allelic replacement of *gfp-ctd* for *ohyA* in the genome for cellular localization studies (Fig. S1). We used super-resolution microscopy to directly measure the location of GFP-CTD and observed green rings (Figs. 13*B* and S19). These data indicate the OhyA CTD peptide(550–591) sequence is sufficient for membrane localization.

## Discussion

Peripheral membrane proteins reversibly bind the surface of membrane bilayers to carry out functions such as cell signaling, enzymatic activity, and membrane remodeling. Thus, they exist in both a soluble and a membrane-bound state (37). Most OhyA characterization has been done on the soluble state of the protein with no consideration of how OhyA accomplishes its task of acquiring substrate unsaturated fatty acid. OhyA is a multidomain flavoenzyme where each domain is dedicated to a step in the unsaturated fatty acid hydration mechanism. A fatty acid lobe contains a hydrophobic tunnel through which unsaturated fatty acid travels to the active site, an FAD lobe contains the FAD binding site and a lid that closes behind FAD and condenses the active site, an active site located at the interface between the two lobes, and an ancillary carboxy-terminal domain of unknown function that does not engage the lobes or stabilize OhyA dimerization. Therefore, additional functional characterization was needed to fundamentally understand the enzymatic mechanism.

**Figure 14:**
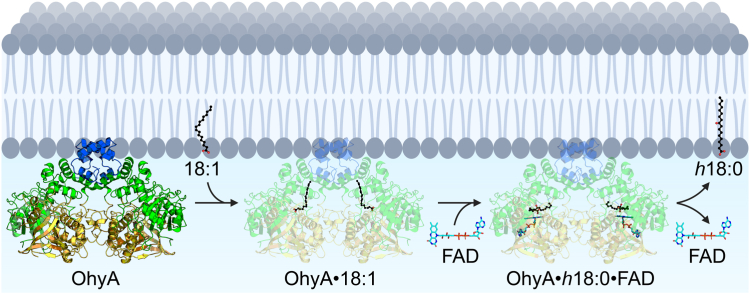
**Model for interfacial assembly of OhyA on the membrane bilayer.** OhyA is a peripheral membrane protein that is anchored to the membrane bilayer through its carboxy terminus (*blue*). OhyA extracts oleate (18:1) from the bilayer, then binds flavin adenine dinucleotide (FAD) for catalysis. OhyA catalyzes water addition to 18:1 to produce 10-hydroxystearic acid (*h*18:0), and then releases FAD into the cytosol and *h*18:0 into the membrane.

In this study, we found the carboxy terminus is a membrane anchor that enables OhyA to solve the topological problem of accessing its insoluble substrate in the membrane bilayer. The OhyA carboxy terminus amino acid sequence intrinsically folds into amphipathic α-helices and binds membranes through electrostatic interactions and hydrogen bonds with the negatively charged phosphate groups of the phospholipids, as well as hydrophobic interactions. CD analysis of a synthetic peptide of the OhyA carboxy-terminal domain shows that the structure in solution is stabilized by phosphate or phospholipids. NMR analysis of the peptide shows the turn connecting the amphipathic helices in the HTH of the carboxy terminus is a flexible hinge. When the peptide is exposed to phosphate or phospholipids in solution, the conformation it adopts is identical to the conformation of the HTH motif in the OhyA crystal structure (1). This suggests that the OhyA carboxy terminus is structured but the HTH motif is flexible in solution and X-ray crystallography has only captured the membrane-bound conformation. Phosphate or membrane binding does not appear to change the secondary structure of the carboxy terminus. Predominant membrane localization of GFP-OhyA in *S. aureus* cells by super-resolution microscopy suggests membrane binding may lock the conformation of the carboxy terminus to retain OhyA on the membrane bilayer. Deletion of the amphipathic helices disrupts OhyA membrane binding and catalysis *in vitro* and *in vivo*. We observed the catalytic defect to persist in assays where unsaturated fatty acid substrate was delivered in liposomes. These data show that OhyA is a peripheral membrane protein and the carboxy terminus is important for membrane binding and unsaturated fatty acid acquisition (Fig. 14).

The discovery that the carboxy terminus amino acid sequence is necessary and sufficient for membrane association is useful for biotechnological applications that require membrane recruitment. Comparison of available OhyA structures shows variability in the length and conformation of the carboxy terminus, and analysis of the amino acids in this region shows notable sequence diversity (Fig. S20). The carboxy terminus sequence may be bioinformatically useful to discriminate between families of OhyA that may bind membrane lipids other than PG or assign predicted functions to proteins that are not known to interact with the membrane but encode a similar sequence. The carboxy terminus is required for its function; however, this key domain is absent in some OhyAs. How these truncated OhyAs perform their function without a carboxy terminus domain is unclear. *Rhodococcus erythropolis* encodes a truncated monomeric OhyA that lacks a carboxy terminus (Fig. S20) but is still functionally active (38). Thus, there are at least two possibilities for how these enzymes accomplish their catalytic task. Truncated OhyAs may acquire substrate from fatty acid binding proteins (*e.g.*, FadL (39) or FakB (12)) through direct protein-protein interaction, or truncated OhyAs may have a unique lipid interface binding site.

### Hydroxy fatty acid transit

Hydroxy fatty acids (*h*FAs) made by OhyA are found in the extracellular environment rather than the cell membrane (6), and extracellular *h*FAs are internalized by mammalian cells to blunt the immune response to infection (7). There are still at least two unknowns to connect what we know about OhyA to what we know about *h*FA. First, how is *h*FA released from OhyA? Unsaturated fatty acid enters OhyA through a hydrophobic tunnel that connects the buried OhyA active site to the protein surface (1); however, the polar C-10 hydroxyl group may exclude *h*FA from diffusing through the greasy tunnel. The FAD binding pocket is continuous with the active site, and the release of FAD after the OhyA reaction could create a path for *h*FA to bulk solvent through this cavity. A putative side opening of the active site lined with water molecules and hydrophilic amino acids may connect to bulk solvent and represent another possible *h*FA escape route (4). We superposed the OhyA•18:1 (PDB ID: 7KAY) and OhyA•PEG400•FAD (PDB ID: 7KAW) crystal structures with the cryo-EM reconstructions from the variability analysis to analyze changes in the fatty acid and FAD lobes. We observed that when OhyA reconstructions have a disordered carboxy terminus they also have solvent-exposed binding sites for both substrates (Fig. S21). These differences are consistent with OhyA undergoing a conformational change to release substrate. Thus, the conformation that allows the uptake of unsaturated fatty acid and FAD may be different than the confirmation that releases *h*FA and FAD.

Second, OhyA is expected to reside on the inner leaflet of the cell membrane but it is undetermined whether OhyA returns *h*FA to the inner leaflet and there is no known fatty acid export system in *S. aureus*. Therefore, the open question is how do *h*FAs get out of the cell? Un-ionized fatty acids can rapidly diffuse by the flip-flop mechanism between the two leaflets of the membrane and can subsequently desorb from the outer leaflet (40). *h*FAs have increased water solubility compared to unsaturated fatty acids and may enter or exit a cell by simple diffusion as monomeric *h*FA without a transport protein (40), although fatty acid binding proteins (*i.e.*, BSA, FABP) would enhance aqueous solubility, bulk transport, and protect cell membranes from damage caused by *h*FAs (41).

### Similarities to mammalian monoamine oxidase (MAO)

MAOs are mitochondrial outer membrane-bound flavoproteins that catalyze the oxidative deamination of neurotransmitters and dietary amines (42), and MAO inhibitors are used to treat neurological disorders (43). MAOs are dimers and X-ray crystal structures show the prototypical MAO structure consists of an FAD-binding lobe, substrate-binding lobe, and carboxy-terminal transmembrane helix mitochondrial membrane-binding domain (44, 45). Structural comparison of human MAO A and B to OhyA shows conservation of the fold across the three enzymes despite low MAO sequence identity to OhyA (∼20%). Informatic analysis of the carboxy termini of OhyA and MAO A and B indicates that all three termini have an overall positive charge (+3 for OhyA and +5 for MAO A and B). The carboxy termini grand average of hydropathicity (46) for OhyA is slightly hydrophilic (−0.631), whereas MAO-A (0.543) and MAO B (0.811) are slightly hydrophobic. The aliphatic index that reflects the relative volume occupied by aliphatic side chains (47) in the OhyA carboxy terminus (106.90) is less than that of MAO-A (128.00) or MAO B (147.57). These values emphasize differences in the MAO transmembrane helices that insert in the membrane and OhyA amphipathic helices that associate with the membrane phosphate layer.

A hydrophobic “entrance” cavity is positioned between the hydrophobic “substrate” active site cavity and the protein surface, and rotation of an isoleucine residue gates the separation or fusion of the cavities (48, 49). Similarly, the rotation of an arginine residue gates the separation or fusion of a hydrophobic tunnel with the active site cavity and makes a continuous channel to the protein surface in OhyA (1). Sequential truncation of the MAO carboxy-terminal transmembrane helix domain progressively decreases the specific activity and destabilizes the protein as the protein no longer associates with the membrane fraction, indicating the membrane environment is required for MAO stability and function (50). These features present intriguing architectural and functional parallels between OhyA and MAO who both utilize a helical carboxy terminus to interact with the membrane bilayer.

## Experimental procedures

### Materials

All chemicals and reagents were obtained from Sigma-Aldrich or Fisher Scientific unless otherwise indicated.

### Molecular biology

#### GFP-OhyA and GFP-CTD knock-in construction in RN4220

GFP-OhyA (CDR001) and GFP-CTD (CDR002) knock-in strains were generated in *S. aureus* strain RN4220 by allelic replacement (Fig. S1). Briefly, 907 bp upstream (Up Arm) and 902 bp downstream (Down Arm) of *ohyA* were amplified by PCR from the RN4220 chromosome using primers Up Arm-F (5′-GAGGCCCTTTCGTCTTCAAGCAATAATTCTCTTGCCGTTC-3′), Up Arm-R (5′-AGTCCTTTCGTATTAGAATACACTCAGACTATACCCCTTTG-3′), Down Arm-F (5′-ATTAATAGATTTTTATTTGGTGATTTCAAATCATGAGACTG-3′), and Down Arm-R (5′-GTCGACTCTAGAGGATCCCCCACTTGAACTTTGAACTAGTG-3′). The *ohyA-gfp* and *ohyA-ctd* genes were constructed to contain an amino terminal His_6_ tag followed by the *gfp* gene that was linked by a flexible linker (amino acid sequence GGGGSGGSS) to *ohyA* or the OhyA carboxy terminus sequence (amino acids 550–591). The initiating methionine residues of GFP and OhyA were mutated to alanines. Gene strings of *ohyA-gfp* and *ohyA-ctd* genes were synthesized by GeneArt Gene Synthesis Technology (Life Technologies). Plasmid vector pJB38 (51, 52) was digested with EcoRI and SmaI and gel-purified. The PCR products and gene strings were moved into pJB38 by Gibson Assembly (New England Biolabs) to generate GFP-OhyA and GFP-CTD knock-in plasmids. The knock-in plasmids were transformed into RN4220 by electroporation, the knock-in strains were generated as previously described (51, 53) and confirmed by PCR (Fig. S1).

#### Plasmid construction for OhyA expression in *E. coli* and *S. aureus* strain USA300

The *S. aureus ohyA* DNA sequence was synthesized and cloned into pET28a with an amino terminal His tag to form pOhyA for overexpression and purification from *E. coli* as previously described (1). The OhyA(ΔHTH) mutation was introduced by modifying pOhyA with a premature stop codon using the QuikChange Lightning site-directed mutagenesis kit (Agilent Technologies) and primers OhyA(ΔHTH)-F (5′-ATTACTAAAGATTCGTAAATGCAAAAACTCGCA-3′) and OhyA(ΔHTH)-R (5′-TGCGAGTTTTTGCATTTACGAATCTTTAGTAAT-3′) to form pOhyA(ΔHTH). The OhyA(X^6^) amino acid sequence contains six point mutations: K563E, M564A, K566E, L567A, K574E, and K575E. The DNA sequence encoding the OhyA(X^6^) construct was optimized for gene expression in *E. coli* using GeneArt Gene Synthesis Technology (Life Technologies) and synthesized for cloning into the pET28a 5′-NdeI site by the Gibson Assembly method and introduce an amino-terminal His_6_-tag. The OhyA, OhyA(ΔHTH), and OhyA(X^6^) inserts were moved from pET28a to pPJ480 (6) for expression in *S. aureus* strain USA300 using 5′-NdeI and 3′-HindIII sites.

### Antibody generation and testing

Polyclonal antibodies against OhyA were generated in rabbits immunized with purified His-tagged OhyA protein by Rockland Immunochemicals, Inc. The specificity of the OhyA antibodies was analyzed using extracts from *S. aureus* strains AH1263 (wildtype USA300), PDJ68/pPJ480 (USA300 Δ*ohyA* with pPJ480 empty plasmid) (6), PDJ68/pPJ480-OhyA (USA300 Δ*ohyA* with pPJ480-OhyA), PDJ68/pPJ480-OhyA(ΔHTH) (USA300 Δ*ohyA* with pPJ480-OhyA(ΔHTH)), PDJ68/pPJ480-OhyA(X^6^) (USA300 Δ*ohyA* with pPJ480-OhyA(X^6^)). Lysates were resolved using a 10% bis-tris acrylamide SDS gel run in 2-(N-morpholino)ethanesulfonic acid (MES) buffer and then transferred to a polyvinylidene difluoride membrane. The blots were blocked for 1 h in 5% milk/TBS-T and then exposed to primary OhyA antibody overnight in 1:10,000 dilution in 5% milk/TBS-T followed by secondary antibody (anti-rabbit Alexa Fluor 488, Sigma) in 5% milk/TBS-T for 1 h at 1:10,000 dilution. The blot was washed extensively and the bands were visualized on a Typhoon FLA9500 imager (GE Healthcare).

### Super resolution microscopy

CDR001 and CDR002 cells were grown in Luria broth to OD_600_ = 0.6 and harvested by centrifugation. Cell pellets were resuspended in 1 ml 1× PBS (Gibco) and incubated with 0.125× CellBrite Fix 640 membrane dye (Biotium) for 15 min at 37 °C. Cells were washed three times with 1 ml PBS and then incubated in 1 ml 4% paraformaldehyde (LifeTechnologies) for 20 min. Cells were washed twice with 1 ml PBS, resuspended in 150 μl PBS, and then plated on a 0.01% poly-L-lysine coverslip (Sigma) for 30 min. The coverslips were washed twice with PBS and then placed on top of slides containing Prolong diamond antifade mountant (LifeTechnologies). AiryScan super-resolution microscopy was conducted on a Zeiss LSM980 microscope using an α Plan-Apochromat 63x/1.4 NA objective. Z stacks were acquired at a digital zoom of 1.7 using 405, 488, and 633 laser lines. AiryScan processing was subsequently performed in Zen Blue 3.7 (Carl Zeiss Microscopy). Processed AiryScan images were then visualized using Imaris (Bitplane).

### Preparation of OhyA, OhyA(ΔHTH), and OhyA(X^6^)

The pOhyA, pOhyA(ΔHTH), and pOhyA(X^6^) plasmids were transformed into *E. coli* BL21(DE3) cells, and isolates were obtained on Luria broth agar imbedded with 50 μg/μl kanamycin (Gold Biotechnology). Transformants were amplified in Luria broth containing 50 μg/μl kanamycin and shaken at 37 °C, 200 rpm. Cells were grown to an OD_600_ of 0.6 and then cooled to 16 °C before overnight induction with 1 mM isopropyl-β-D-thiogalactoside (Gold Biotechnology). Cells were harvested and then lysed in a buffer containing 20 mM Tris, pH 8.0, 10 mM imidazole, 200 mM NaCl, and a dissolved tablet of Pierce protease inhibitor. The OhyA, OhyA(ΔHTH), and OhyA(X^6^) proteins were separated from cell lysates by nickel agarose beads (Gold Biotechnology) and eluted in buffer containing 20 mM Tris, pH 8.0, 250 mM imidazole, and 200 mM NaCl. The eluant was gel filtered into a buffer containing 20 mM Tris, pH 8.0, and 200 mM NaCl using the HiLoad Superdex 200 column (Cytiva Life Sciences) with dimensions 16 mm × 60 cm. The molecular weights of the OhyA, OhyA(ΔHTH), and OhyA(X^6^) proteins were estimated by SEC-MALS. Protein resistance to thermal denaturation was determined by Sypro Orange-based fluorescence assay (54). Solutions (30 μl) of 1 mg/ml OhyA, OhyA(ΔHTH), or OhyA(X^6^) in 50 mM K_2_HPO_4_, 150 mM NaCl, pH 6, and 2.5× Sypro Orange dye were added to wells of ThermoGrid optically clear PCR plates (Denville Scientific). The plates were centrifuged at 1000*g* for 5 min and then analyzed by the ABI 7300 real-time PCR system. The temperature was ramped from 25 °C to 95 °C at 1 °C/min with the fluorescence read six times at each temperature ramp. The resulting data were fit to a Boltzmann sigmoidal equation to determine the melting point of each enzyme. Each enzyme was repeated five times and the thermal melting temperature of each replicate was determined independently. The melting points from each replicate were averaged to determine the reported thermal melting point.

### Size-exclusion chromatography multi-angle light scattering (SEC-MALS)

SEC-MALS experiments were carried out using a Superose 6 Increase 10/300 Gl (M_W_ range 5000–1,250,000 Da) size-exclusion column (Cytiva Life Sciences) with three detectors connected in series: an Agilent 1200 UV detector (Agilent Technologies), a Wyatt DAN HELEOS II multi-angle light-scattering and a Wyatt Optilab T-rEX differential refractive index detector (Wyatt Technologies) (55, 56). The Superose 6 Increase 10/300 Gl column was equilibrated in SEC-MALS buffer (20 mM Tris, 200 mM NaCl, pH 7.6), and all data were collected at 25 °C. OhyA, OhyA(ΔHTH), and OhyA(X^6^) were prepared at 2 mg/ml in SEC-MALS buffer, and 100 μl aliquots were injected into the column using an auto-sample injection method and a flow rate of 0.5 ml/min. Protein in the eluent was detected by light scattering, UV absorbance at 280 nm, and refractive index detectors. The data were recorded and analyzed with the Wyatt Astra software (version 8). The data were plotted as a molar mass distribution superimposed on a chromatogram of absorbance at 280 nm *versus* elution volume.

### Mass photometry

Mass photometry measurements were performed on a OneMP mass photometer (Refeyn Ltd). To prepare the measurements, borosilicate microscope coverslips were cleaned sequentially with acetone, isopropanol, and Milli-Q-water followed by drying under a stream of clean nitrogen. Silicone gaskets were placed onto the cleaned coverslips to create wells for sample application. For mass measurements, gaskets were filled with 20 μl measurement buffer to allow focusing the microscope onto the coverslip surface. Subsequently, 3 μl of the protein sample were added into the gasket to achieve a final concentration of 100 nM protein. Sample binding to the coverslip surface was monitored using the software AcquireMP (Refeyn Ltd). Data analysis was performed using DiscoverMP (Refeyn Ltd). To convert the measured optical reflection-interference contrast into a molecular mass, a known protein size marker (NativeMarkTM Unstained Protein Standard, Invitrogen) was referenced.

### Preparation of unilamellar vesicles

DOPG (1,2-dioleoyl-sn-glycero-3-phosphoglycerol; Avanti Polar Lipids) and/or DOPC (1,2-dioleoyl-sn-glycero-3-phosphocholine; Avanti Polar Lipids) lipids dissolved in chloroform were mixed so that the total lipid concentration would be 1 mM in 1 ml, and then blown down under N_2_ gas. Dried lipids were resuspended in 1 ml of 50 mM potassium phosphate, 150 mM sodium chloride, pH 6.0, and hydrated for 1 h at 37 °C. Lipids were sonicated twice prior to extrusion to prepare liposomes of 100 nm diameter.

### Surface plasmon resonance

Surface plasmon resonance experiments were conducted at 25 °C on a Biacore S200 optical biosensor (Cytiva Life Sciences) using the methods of Del Vecchio and Stahelin (57). DOPG:DOPC (50:50) unilamellar vesicles were used for the variable component vesicles, and DOPC unilamellar vesicles were used for the control vesicles to allow for measurement of net binding to DOPG. Unilamellar vesicles were captured on an equilibrated, pre-conditioned L1 chip (Cytiva Life Sciences) until ∼5000 to 6000 resonance units of lipids were captured, and the chip was blocked with 3 to 4 injections of 0.1 mg/ml BSA. Small unilamellar phospholipid vesicles are captured on the L1 chip through hydrophobic interactions with the hydrogel layer on the chip. DOPC was used to stabilize DOPG:DOPC (50:50) lipid immobilization on the L1 chip because higher levels of DOPG yielded unstable surfaces, possibly caused by the net negative charge on the carboxymethyl dextran-based hydrogel that may cause electrostatic-repulsion-of-lipids with negatively charged headgroups like DOPG.

OhyA, OhyA(ΔHTH), and OhyA(X^6^) were prepared in the binding buffer as a three-fold dilution series with a maximum concentration of 20 μM, and OhyA CTD peptide was prepared in binding buffer as a two-fold dilution series with a maximum concentration of 24 μM. OhyA, OhyA(ΔHTH), OhyA(X^6^), or OhyA CTD peptide(550–591) were injected for 600 s at a flow rate 10 μl/min (association phase) and then allowed to dissociate for 600 s for each cycle. A series of buffer-only (blank) injections was included throughout the experiment to account for instrumental noise. The lipid surfaces were regenerated between cycles with 50 mM NaOH injected for 12 s at a flow rate 50 μl/min. The data were processed, referenced, and analyzed using the Biacore S200 Evaluation Software. Equilibrium binding levels were determined and exported to GraphPad Prism for fitting to the Hill equation.

### OhyA assay

OhyA activity was measured in 20 μl reactions containing 50 mm potassium phosphate, pH 6.0, 150 mm sodium chloride, 10 mm dithiothreitol, 50 μm FAD, 20 μm [1-^14^C]18:1(9*Z*) (specific activity 54.3 mCi/mmol) (PerkinElmer), and 100 μM DOPC:DOPG (50:50) unilamellar vesicles to deliver [1-^14^C]18:1(9*Z*). OhyA and OhyA(X^6^) were assayed at 0.05 mg/ml, whereas OhyA(ΔHTH) was assayed at 0.5 mg/ml. Enzyme concentrations were experimentally determined to yield detectable hydroxy fatty acid product formation. Reactions were incubated at 30 °C for 20 min and were spotted onto silica gel H TLC plates developed with choloroform:methanol (90/10, v/v). The pPJ480, pPJ480-OhyA, pPJ480-OhyA(ΔHTH), and pPJ480-OhyA(X^6^) expression plasmids were electroporated into the *S. aureus* USA300 Δ*ohyA* strain (6) to generate four plasmid-bearing Δ*ohyA* strains. The plasmid-bearing strains were inoculated into 100 μl lysogeny broth containing 10 μg/ml chloramphenicol and 1 mg/ml fatty acid-free bovine serum albumin. At OD_600_ = 0.5, [1-^14^C]18:1(9*Z*) (specific activity 54.3 mCi/mmol) (PerkinElmer) was added to 50 μM and the cultures incubated without shaking at 37 °C for 4 h. After incubation, the cells were pelleted by centrifugation and 10 μl of the culture supernatant was spotted onto silica gel H TLC plates developed with choloroform:methanol (90/10, v/v). The distributions of radioactivity on the dried plates and the extent of product formation were quantified using a Typhoon PhosphorImager.

### Synthesis of OhyA CTD peptide(550–591)

OhyA CTD peptide(550–591) was synthesized on a LibertyBlue Microwave Peptide synthesizer using standard Fmoc-based chemistry. The peptide were synthesized with an acetylated amino terminus and peptide couplings were performed using diisopropylcarbodiimide (DIC) at 75 °C. After the synthesis, the resin was treated with a 10% solution of acetic anhydride in N-methyl pyrrolidone (NMP), to introduce the N-terminal acetyl group. The peptide was then cleaved from the resin using trifluoroacetic acid:water:thioanisole:triisopropylsilane:phenol:1,2-ethanedithiol (82.5:5:5:2.5:2.5:2.5) and precipitated using ice-cold diethyl ether. The precipitated peptide was centrifugalized and then resuspended in acetonitrile:water (50:50), diluted further with water, and lyophilized. The crude peptide was HPLC purified using a Waters Binary Prep HPLC system equipped with a fraction collector. Fractions were analyzed using a Waters Analytical HPLC system equipped with a UV detector and a Bruker MicroFlex MALDI-TOF mass spectrometer. Pure fractions were pooled together and lyophilized to obtain the purified peptide.

### Circular dichroism (CD) spectroscopy

CD spectra were collected between 180 and 260 nm with a 2 nm data pitch and 1 nm bandwidth at 23 °C using a JASCO J-1500 CD spectrophotometer and a quartz cuvette with a 1 mm pathlength. Cuvettes contained 300 μl samples of 100 μM OhyA CTD peptide(550–591) dissolved in water, 150 mM NaCl, 50 mM potassium phosphate, or 2 mM DOPG small unilamellar phospholipid vesicles. CD Temperature scans of OhyA CTD peptide(550–591) and OhyA CTD peptide(550–591) with DOPG were collected between 198 and 250 nm with a 2 nm data pitch and 1 nm bandwidth from 25 °C to 85 °C in 5 °C increments. Measurements were recorded as five accumulations, and the average was plotted.

### Nuclear magnetic resonance (NMR) spectroscopy

#### Sample preparation

OhyA CTD peptide(550–591) was synthesized and purified as described earlier. For NMR experiments, the peptide was dissolved in 20 mM Tris buffer with either 150 mM NaCl or 50 mM K_2_HPO_4_ at pH 6 containing 10% D_2_O, to a final peptide concentration of one or 2 mM. Glycerol and liposomes were titrated into the NMR tube in 150 mM NaCl at different concentrations as mentioned in the text. For K_D_ determination, the intensity of the bound species is determined as, B = 1-(I_B_/I_F_), where I_B_ and I_F_ are the bound and the free peak intensities as given in (34).

#### Data collection and processing

All the NMR experiments were performed at 30 °C using a 600 or 700 MHz Bruker Avance NMR spectrometer equipped with a triple-resonance cryogenic probe. Resonance assignments were done using a standard approach using 2D [^1^H, ^1^H] COSY, TOCSY, NOESY spectra recorded with 70 and 150 ms mixing time for the TOCSY and NOESY spectra respectively. Natural abundance 2D [^13^C, ^1^H] HSQC and 2D [^15^N,^1^H] sofast-HMQC spectra were recorded with 160 and 512 scans respectively. All the data were processed using NMRPipe (58) and analyzed using NMRFAM-SPARKY (59).

#### Structure calculation

The structure calculations were carried out in NaCl and K_2_HPO_4_ solvents with the CYANA 3.98.15 program (60) using simulated annealing in combination with molecular dynamics in torsion angle space. Dihedral angle restraints were derived from the carbon chemical shifts using Talos (61). Hydrogen bond restraints were added after the initial structure calculation using only the NOE data, for those amides that did not show an exchange cross-peak with water in the 2D NOESY spectra. The assignment of the NOESY peaks was done using automatic NOE assignment in CYANA using the seven default cycles. A total of 100 conformers were calculated using 10,000 annealing steps for each conformer after the complete assignment of resonances. The lowest 20 energy conformers were selected to represent the bundle of NMR structures and they were energy-minimized using CNS. The lowest energy conformer is used as a representative structure for all analyses.

### Molecular dynamics (MD) simulations

#### OhyA carboxy terminus fragment and membrane bilayer model preparation

The carboxy-terminal domain (CTD, residues 550–591) of OhyA was extracted from the crystal structure (PDB ID: 7KAV) as the initial structure for the simulations (1). The protonation states of protein residues were determined by the PROPKA3 (62), and missing hydrogens were added using the PSFGEN plugin of Visual Molecular Dynamics (VMD) (63). The CTD fragment was capped with an acetylated amino terminus (ACE) and N-Methylamide carboxy terminus (CT3) groups using the PSFGEN plugin. In order to capture the CTD fragment binding the membrane, we employed the Highly Mobile Membrane Mimetic (HMMM) model (32, 64, 65, 66) to accelerate processes limited by lateral lipid diffusion (such as spontaneous protein insertion). We used the Bilayer Builder module of CHARMM-GUI (67, 68) to construct an 82 Å × 82 Å × 46 Å full membrane (FM) with 100 DOPG molecules in each leaflet and obtain a phosphatidylglycerol (PG) HMMM membrane. We truncated the DOPG molecules at carbon atoms C26 and C36, which correspond to the sixth carbon atom in each acyl chain, and filled the gap between the membrane leaflets with the organic solvent, Simple Carbon Solvent Ethane (SCSE) (33).

#### Membrane binding simulations with the OhyA carboxy terminus fragment

The center of mass (COM) of the CTD fragment was initially placed 55 Å away from the midplane of the membrane along the membrane normal (z-axis). The initial orientation of the CTD fragment was randomized by rotating in 15° increments around the x-axis to produce 24 starting orientations covering a full 360° rotation. Each CTD fragment•HMMM system was solvated within a water box using the SOLVATE plugin of VMD and subsequently neutralized with 0.15 M NaCl by the AUTOIONIZE plugin of VMD. The resulting systems were minimized for 10,000 steps and equilibrated for 10 ns, during which the protein and the positions of the C2, C26, and C36 atoms of lipid tails were harmonically restrained to their initial coordinates by gradually reducing force constants (*k*) (starting at 1 kcal mol^−1^ Å^−2^ and later reduced to 0.5 kcal mol^−1^ Å^−2^). The production simulations were then performed for 200 ns for each orientation. During the production simulations, the z coordinates of C2, C26, and C36 atoms of phospholipids were harmonically restrained with a force constant of 0.05 kcal mol^−1^ Å^−2^ to prevent short-tailed lipids from occasionally diffusing into the solution. A grid-based potential (69) was employed to confine SCSE solvent molecules to the membrane core.

The initial frame of a membrane-bound configuration of the CTD fragment was defined when a protein atom inserted 3 Å beneath the *cis* phosphorous plane of the bilayer and sustained contact with the membrane for a minimum of 30 ns (Fig. S7). Successive membrane-bound segments were joined together if the gap between them was less than 3 ns. Simulation replica x105 which captured the longest lasting membrane-bound configurations (Fig. S7) following the criteria established above, was converted into a full-membrane (FM) simulation to assess the stability of the membrane-bound state captured by the HMMM simulations in an FM environment. SCSE solvent molecules were removed from the last frame of the selected replica and the short-tailed lipids were extended to form DOPG lipids using the PSFGEN plugin to convert the CTD fragment•HMMM system to a CTD fragment•FM one. The CTD fragment•FM system was minimized for 10,000 steps followed by a three-step equilibration process with successively relaxed restrains. First, all components except for the lipid tails were restrained, and the system was simulated in an NVT ensemble for 10 ns to promote the melting of the newly introduced lipid tails. Next, the CTD fragment and head groups of the membrane leaflet interacting with the fragment were restrained, and the system was simulated with an NPT ensemble for 10 ns to prevent dissociation of the CTD fragment from the membrane. Lastly, only the backbone-heavy atoms of the CTD fragment were restrained, and the system was simulated as an NPT ensemble for 10 ns. All restraints in the equilibration simulations used a force constant of 1 kcal mol^−1^ Å^−2^. The equilibrated CTD fragment•FM system was then further simulated for 50 ns without any restraints.

#### OhyA dimer model preparation

The equilibrated CTD fragment•FM system was used to construct a membrane-bound model for the full OhyA dimer while preserving the local interactions between the protein and the lipids. Direct overlay of one protomer from the crystallographic dimer (PDB ID: 7KAV) onto the membrane-bound monomeric CTD fragment resulted in steric clashes between the membrane and the other OhyA protomer (Fig. S14). Helix α19 in the fragment changes orientation from diagonal (starting conformation, Fig. 1*B*) to horizonal with respect to the membrane surface upon membrane binding (Fig. S10). The steric clash between the membrane and OhyA dimer was caused by the crystallographic diagonal conformation of α19 (Figs. 1*B* and S14) that is incompatible with membrane binding.

To sample alternative conformations and obtain a more compatible CTD for membrane binding, the OhyA dimer was prepared for a simulation in water using the default settings of the “Structure Preparation” and “Protonate 3D” tools of Molecular Operating Environment (MOE, Chemical Computing Group ULC, version 2019.01) and solvated in a water box with 0.15 M NaCl using the “Solution Builder” module of CHARMM-GUI (67). The obtained OhyA dimer•water box system was simulated for 1.1 μs following the equilibrium steps recommended by CHARMM-GUI. First, the principal axis of the dimer snapshots obtained from the trajectories were aligned to the membrane normal (z axis) (Fig. S15, *C* and *D*). We then measured the angle θ_1_ between the third principal axis of α19 and the z axis for the OhyA dimer for congruency with the analogous angle θ_2_ for the last frame of the CTD fragment-FM simulation (Fig. S15*D*). The OhyA dimer conformation with a minimum difference between θ_1_ and θ_2_ was selected as the starting protein conformation for the membrane-bound full OhyA dimer simulations (Fig. S15*D*). The optimal alignment region used for the superimposition of the selected OhyA dimer conformation onto the FM-bound CTD fragment was determined according to two criteria: the tilt angle of the dimer with respect to the membrane normal (β), and the RMSD between α19 in the FM-bound CTD fragment and α19 in the OhyA dimer (Fig. S16). OhyA has a twofold symmetry; therefore, the protein is not expected to significantly tilt once it binds to the membrane surface with both protomers’ carboxy termini. 2D sequence matrices containing tilt angles or RMSD values where entries in the i-th row and j-th column (with i < j) denotes the chosen metric value (β or RMSD) calculated after alignment from residue i to j (Fig. S16). The normalized dimer tilt angle and RMSD matrices were summed to identify optimal sequence alignment regions that minimized tilting and RMSD. Residues 558 to 585 were chosen from the top ten alignment regions with the lowest summations, and were used to superimpose the relaxed OhyA dimer from the water box simulation onto the FM-bound CTD fragment to model the FM-bound OhyA dimer (Fig. 11*A*). This method produced an OhyA dimer with a tilt angle of 7.86° and an RMSD of 1.54 Å *versus* the CTD fragment.

#### Membrane-bound simulations of OhyA dimer

The OhyA dimer•FM system was inserted into a larger membrane to prevent self-interaction of protein subunits across the periodic boundaries. The FM-bound OhyA dimer and DOPG molecules within 30 Å of the protein were embedded into a 150 Å × 150 Å DOPG membrane obtained from CHARMM-GUI (67, 68) and the overlapping phospholipids in the membrane were removed. This method preserved the local interactions between the dimer CTD and membrane lipids that were originally captured by CTD fragment•HMMM and CTD fragment•FM simulations. The OhyA dimer•FM system was solvated with TIP3P water (70, 71) and neutralized with 0.15 M NaCl. The system was minimized for 10,000 steps followed by a three-step equilibration process with successively relaxed restrains. Initially, only lipid tails were allowed to move freely for 10 ns, and then only the protein was restrained while the rest of the system moved freely for 20 ns. Finally, only the protein backbone was restrained for 20 ns. All restraints during the equilibrium simulations used a force constant of 1 kcal mol^−1^ Å^−2^. The equilibrated OhyA dimer•FM system was used for two 1.0 μs production simulations.

#### Simulation protocols

The simulations were performed using NAMD (72, 73). All simulations used CHARMM36m and CHARMM36 force fields for protein and lipids, respectively. The TIP3P water model was used (71, 74, 75). Non-bonded interactions were calculated with a 12 Å cutoff and a switching distance of 10 Å. The particle mesh Ewald (PME) method was used to estimate long-range electrostatic interactions (76), the Langevin thermostat with a damping coefficient of 1.0 ps^−1^ was used to maintain the temperature at 310 K, and the Nosé–Hoover Langevin piston method was used to maintain pressure at 1 bar (77). A flexible cell was enabled to allow the system size to adjust in three dimensions independently, while the *x*/*y* ratio of the membrane was held constant. Bonds with hydrogens were kept rigid by SHAKE (78) and SETTLE algorithms (79), and a time step of 2 fs was used for all the simulations.

#### Data analysis

The analysis involving "membrane-bound frames" was conducted using the frames that followed the initial bound frame. The initial bound frame of each CTD fragment•HMMM replica can be found in Fig. S7. The initial bound frame for OhyA dimer•FM replicas was calculated as 0 ns using the criteria detailed above.

### Cryo-electron microscopy (Cryo-EM)

#### OhyA-bound liposome preparation

A 1:1 mixture of 1-palmitoyl-2-oleoyl-sn-glycero-3-phospho-L-glycerol (POPG):1-palmitoyl-2-oleoyl-glycero-3-phosphocholine (POPC) dissolved in chloroform were dried under N_2_. The lipids were redissolved in pentane and dried again. The lipids were incubated under vacuum for 4 h, resuspended in 20 mM HEPES, pH 7.3, 150 mM KCl to 20 mM lipids, and then sonicated to clarity. Sodium cholate was added to 40 mM and the mixture was sonicated briefly to prepare the liposome sample. The sample was incubated at 4 °C for 1 h. Detergent was removed using four exchanges of 300 mg/ml Bio-Beads for 3 h, 3 h, 16 h, and 3 h at 4 °C. OhyA was added to the liposomes at a protein-to-lipid ratio of 1:20 (wt/wt) with the final lipid concentration of 17.5 mM. OhyA-bound liposomes were used immediately to prepare grids.

#### Grid preparation

Grids were initially prepared of OhyA in the absence of detergent because the protein is soluble. Most of the resulting particles were surprisingly dimers of dimers. These particles displayed a severe orientation bias presumably due to the interaction of their carboxy-terminal membrane association domains with the hydrophobic air-water interface. To alleviate this phenomenon, detergent was added.

For the dimer reconstruction, OhyA was first diluted 20-fold in buffer supplemented with 50 μM LMNG prior to re-concentrating to 3 mg/ml for grid preparation. The sample was applied to freshly glow-discharged UltrAufoil R 1.2/1.3300 mesh grids before freezing in liquid ethane using a Vitrobot Mark IV (FEI). 3 μl of the sample was applied to the grids and blotted at a force of −4 for 3 s while exposed to 100% humidity and 10 °C.

For the OhyA bound liposome projections, the sample was applied to freshly glow-discharged UltrAufoil R 0.6/1.0300 mesh grids before freezing. 3.5 μl of the sample was applied to the grids prior to a 20 s wait time and blotted at a force of 1 for 3 s while exposed to 100% humidity and 10 °C.

#### Data collection

Cryo-EM data were collected using a Talos Arctica electron microscope (FEI) operated at 200 kV with a K3 BioQuantum direct electron detector (Gatan) operated in super-resolution mode (0.522 Å/pixel). Data acquisition was automated through EPU (Thermo Fisher Scientific). The electron dose rate was 1.02 e−/Å^2^/frame, and the total dose was 50 e−/Å^2^ divided into 50 subframes.

#### Data processing

Images were binned by two for a pixel size of 1.044 Å/pixel before patch motion correction in cryoSPARC (80). Patch contrast transfer functions (CTF) were estimated for each micrograph. The micrographs were manually curated for ice quality and thickness, CTF fit, and other parameters. Templates were prepared from a generated model map of the previous dimeric crystal structure of OhyA.

For the dimer reconstruction, 3,065,496 particles were initially picked from 3246 micrographs. After two rounds of 2D classification, the selected classes accounted for 1,887,514 particles. Four *ab initio* classes were generated from the selected 2D classification particles. Two rounds of hetero refinement yielded four classes comprised of a good dimer class, a dimer of dimers class with a severely biased particle orientation distribution, a poor dimer class, and a fourth junk class. Particles from the good dimer class were aligned using non-uniform refinement with no symmetry applied. 3D classification without alignment yielded two classes that showed contamination from a dimer of dimers particles. Particles from the remaining eight classes were aligned using CTF refinement and non-uniform refinement with C2 symmetry applied.

3D variability analysis was performed using three orthogonal principal modes (81). The output volume series and starting particles were used to display the 3D variability in a cluster mode in which 20 clusters were fit to the reaction coordinate with volumes and particle output for each cluster. Two new reconstructions were generated from these clusters. In the first, particles from nine of these clusters corresponding to the best-ordered reconstructions for the membrane association domains and FAD pocket were combined. For the second, particles from two of the clusters corresponding to the least ordered reconstructions for the membrane association domains and FAD pocket were combined. For both, a mask around the reconstruction padded by 6 Å was used in subsequent non-uniform refinement yielding resolutions of 2.67 Å and 3.03 Å.

For the OhyA-bound liposome 2D classification, 279,980 particles were picked from 192 micrographs. A representative particle is shown in Figure 4*A*. OhyA has a strict preference for binding small liposomes with a diameter of less than 45 nm, making it difficult to pick and align top views of OhyA bound to liposomes in orthogonal views to the membrane bilayer. This may be caused by an OhyA propensity to form curved oligomeric assemblies (dimer of dimers and coil structures). 2D classification produced several good OhyA-bound liposome classes representing 128,540 particles. One such class, representing 5374 particles, is shown in Figure 4*B*.

The resolution of the final OhyA dimer map was determined from the Fourier shell correlation (FSC) of two reconstructions generated from half of the data and a cutoff criterion of 0.143. The final map was locally masked and sharpened using DeepEMhancer (82). Estimation of local resolution was performed using Blocres (83).

#### Model building, refinement, and validation

The coordinates for OhyA (PDB ID: 7KAV) were docked into the dimer reconstruction. One of the half maps was used in PHENIX (84) for real space refinements. The final model was validated using MolProbity (85). Figures were created in ChimeraX (86) and PyMOL (87).

### Statistical analysis

All statistical analyses and mathematical modeling (*i.e.*, Hill and Boltzmann equations) were performed using GraphPad Prism software version 9.1.1.

## Data availability

The NMR chemical shifts and structures of the OhyA CTD peptide(550–591) in NaCl and K_2_HPO_4_ are deposited in the BMRB with accessions 31,112 and 31,113 respectively, and in the Protein Data Bank with accessions 8UM1 and 8UM2 respectively. The Cryo-EM maps and reconstructions of the OhyA dimer with an ordered CTD and disordered CTD are deposited in the EMDB with accessions EMD-42480 and EMD-42484 respectively, and in the Protein Data Bank with accessions 8UR3 and 8UR6 respectively.

## Supporting information

This article contains supporting information.

## Conflict of interest

The authors declare that they have no conflicts of interest with the contents of this article.

## References

[1] Radka C.D., Batte J.L., Frank M.W., Young B.M., Rock C.O. (2021). Structure and mechanism of *Staphylococcus aureus* oleate hydratase (OhyA). J. Biol. Chem..

[2] Yang B., Gao H., Stanton C., Ross R.P., Zhang H., Chen Y.Q. (2017). Bacterial conjugated linoleic acid production and their applications. Prog. Lipid Res..

[3] Hagedoorn P.L., Hollmann F., Hanefeld U. (2021). Novel oleate hydratases and potential biotechnological applications. Appl. Microbiol. Biotechnol..

[4] Prem S., Helmer C.P.O., Dimos N., Himpich S., Bruck T., Garbe D. (2022). Towards an understanding of oleate hydratases and their application in industrial processes. Microb. Cell Fact..

[5] Chalmers S.J., Wylam M.E. (2020). Methicillin-resistant *Staphylococcus aureus* infection and treatment options. Methods Mol. Biol..

[6] Subramanian C., Frank M.W., Batte J.L., Whaley S.G., Rock C.O. (2019). Oleate hydratase from *Staphylococcus aureus* protects against palmitoleic acid, the major antimicrobial fatty acid produced by mammalian skin. J. Biol. Chem..

[7] Radka C.D., Batte J.L., Frank M.W., Rosch J.W., Rock C.O. (2021). Oleate hydratase (OhyA) is a virulence determinant in *Staphylococcus aureus*. Microbiol. Spectr..

[8] Malachowa N., Kohler P.L., Schlievert P.M., Chuang O.N., Dunny G.M., Kobayashi S.D. (2011). Characterization of a *Staphylococcus aureus* surface virulence factor that promotes resistance to oxidative killing and infectious endocarditis. Infect. Immun..

[9] Mukerjee P. (1965). Dimerization of anions of long-chain fatty acids in aqueous solutions and the hydrophobic properties of the acids. J. Phys. Chem..

[10] Douliez J.P., Navailles L., Nallet F. (2006). Self-assembly of fatty acid-alkylboladiamine salts. Langmuir.

[11] Smith A., Lough A.K. (1976). Micellar solubilization of fatty acids in aqueous media containing bile salts and phospholipids. Br. J. Nutr..

[12] Radka C.D. (2023). Interfacial enzymes enable Gram-positive microbes to eat fatty acids. Membranes (Basel).

[13] Cornell R.B., Kalmar G.B., Kay R.J., Johnson M.A., Sanghera J.S., Pelech S.L. (1995). Functions of the C-terminal domain of CTP: phosphocholine cytidylyltransferase. Effects of C-terminal deletions on enzyme activity, intracellular localization and phosphorylation potential. Biochem. J..

[14] Kalmar G.B., Kay R.J., Lachance A., Aebersold R., Cornell R.B. (1990). Cloning and expression of rat liver CTP: phosphocholine cytidylyltransferase: an amphipathic protein that controls phosphatidylcholine synthesis. Proc. Natl. Acad. Sci. U. S. A..

[15] Wang Y., Kent C. (1995). Identification of an inhibitory domain of CTP:phosphocholine cytidylyltransferase. J. Biol. Chem..

[16] Takei K., Slepnev V.I., Haucke V., De Camilli P. (1999). Functional partnership between amphiphysin and dynamin in clathrin-mediated endocytosis. Nat. Cell Biol..

[17] Peter B.J., Kent H.M., Mills I.G., Vallis Y., Butler P.J., Evans P.R. (2004). BAR domains as sensors of membrane curvature: the amphiphysin BAR structure. Science.

[18] Richnau N., Fransson A., Farsad K., Aspenstrom P. (2004). RICH-1 has a BIN/Amphiphysin/Rvsp domain responsible for binding to membrane lipids and tubulation of liposomes. Biochem. Biophys. Res. Commun..

[19] Khan F.I., Lan D., Durrani R., Huan W., Zhao Z., Wang Y. (2017). The lid domain in lipases: structural and functional determinant of enzymatic properties. Front. Bioeng. Biotechnol..

[20] Wootan M.G., Storch J. (1994). Regulation of fluorescent fatty acid transfer from adipocyte and heart fatty acid binding proteins by acceptor membrane lipid composition and structure. J. Biol. Chem..

[21] Herr F.M., Matarese V., Bernlohr D.A., Storch J. (1995). Surface lysine residues modulate the collisional transfer of fatty acid from adipocyte fatty acid binding protein to membranes. Biochemistry.

[22] Corsico B., Cistola D.P., Frieden C., Storch J. (1998). The helical domain of intestinal fatty acid binding protein is critical for collisional transfer of fatty acids to phospholipid membranes. Proc. Natl. Acad. Sci. U. S. A..

[23] Corsico B., Liou H.L., Storch J. (2004). The alpha-helical domain of liver fatty acid binding protein is responsible for the diffusion-mediated transfer of fatty acids to phospholipid membranes. Biochemistry.

[24] Volkov A., Khoshnevis S., Neumann P., Herrfurth C., Wohlwend D., Ficner R. (2013). Crystal structure analysis of a fatty acid double-bond hydratase from *Lactobacillus acidophilus*. Acta Crystallogr. D Biol. Crystallogr..

[25] Engleder M., Pavkov-Keller T., Emmerstorfer A., Hromic A., Schrempf S., Steinkellner G. (2015). Structure-based mechanism of oleate hydratase from *Elizabethkingia meningoseptica*. Chembiochem.

[26] Park A.K., Lee G.H., Kim D.W., Jang E.H., Kwon H.T., Chi Y.M. (2018). Crystal structure of oleate hydratase from *Stenotrophomonas* sp. KCTC 12332 reveals conformational plasticity surrounding the FAD binding site. Biochem. Biophys. Res. Commun..

[27] Wu D., Piszczek G. (2021). Standard protocol for mass photometry experiments. Eur. Biophys. J..

[28] Fineberg A., Surrey T., Kukura P. (2020). Quantifying the monomer-dimer equilibrium of tubulin with mass photometry. J. Mol. Biol..

[29] Zhao J., Benlekbir S., Rubinstein J.L. (2015). Electron cryomicroscopy observation of rotational states in a eukaryotic V-ATPase. Nature.

[30] Wimley W.C., White S.H. (1996). Experimentally determined hydrophobicity scale for proteins at membrane interfaces. Nat. Struct. Biol..

[31] Miles A.J., Wallace B.A. (2016). Circular dichroism spectroscopy of membrane proteins. Chem. Soc. Rev..

[32] Ohkubo Y.Z., Pogorelov T.V., Arcario M.J., Christensen G.A., Tajkhorshid E. (2012). Accelerating membrane insertion of peripheral proteins with a novel membrane mimetic model. Biophys. J..

[33] Vermaas J.V., Pogorelov T.V., Tajkhorshid E. (2017). Extension of the highly mobile membrane mimetic to transmembrane systems through customized in silico solvents. J. Phys. Chem. B.

[34] Shortridge M.D., Hage D.S., Harbison G.S., Powers R. (2008). Estimating protein-ligand binding affinity using high-throughput screening by NMR. J. Comb. Chem..

[35] Nanga R.P., Brender J.R., Vivekanandan S., Ramamoorthy A. (2011). Structure and membrane orientation of IAPP in its natively amidated form at physiological pH in a membrane environment. Biochim. Biophys. Acta.

[36] Buffy J.J., Hong T., Yamaguchi S., Waring A.J., Lehrer R.I., Hong M. (2003). Solid-state NMR investigation of the depth of insertion of protegrin-1 in lipid bilayers using paramagnetic Mn^2+^. Biophys. J..

[37] Larsen A.H., John L.H., Sansom M.S.P., Corey R.A. (2022). Specific interactions of peripheral membrane proteins with lipids: what can molecular simulations show us?. Biosci. Rep..

[38] Lorenzen J., Driller R., Waldow A., Qoura F., Loll B., Bruck T. (2018). *Rhodococcus erythropolis* oleate hydratase: a new member in the oleate hydratase family tree-biochemical and structural studies. Chemcatchem.

[39] Black P.N. (1990). Characterization of FadL-specific fatty acid binding in *Escherichia coli*. Biochim. Biophys. Acta.

[40] Ek-Von Mentzer B.A., Zhang F., Hamilton J.A. (2001). Binding of 13-HODE and 15-HETE to phospholipid bilayers, albumin, and intracellular fatty acid binding proteins. Implications for transmembrane and intracellular transport and for protection from lipid peroxidation. J. Biol. Chem..

[41] Glatz J.F., Borchers T., Spener F., van der Vusse G.J. (1995). Fatty acids in cell signalling: modulation by lipid binding proteins. Prostaglandins Leukot. Essent. Fatty Acids.

[42] Edmondson D.E., Mattevi A., Binda C., Li M., Hubalek F. (2004). Structure and mechanism of monoamine oxidase. Curr. Med. Chem..

[43] Youdim M.B., Edmondson D., Tipton K.F. (2006). The therapeutic potential of monoamine oxidase inhibitors. Nat. Rev. Neurosci..

[44] Binda C., Newton-Vinson P., Hubalek F., Edmondson D.E., Mattevi A. (2002). Structure of human monoamine oxidase B, a drug target for the treatment of neurological disorders. Nat. Struct. Biol..

[45] Ma J., Yoshimura M., Yamashita E., Nakagawa A., Ito A., Tsukihara T. (2004). Structure of rat monoamine oxidase A and its specific recognitions for substrates and inhibitors. J. Mol. Biol..

[46] Kyte J., Doolittle R.F. (1982). A simple method for displaying the hydropathic character of a protein. J. Mol. Biol..

[47] Ikai A. (1980). Thermostability and aliphatic index of globular proteins. J. Biochem..

[48] Binda C., Li M., Hubalek F., Restelli N., Edmondson D.E., Mattevi A. (2003). Insights into the mode of inhibition of human mitochondrial monoamine oxidase B from high-resolution crystal structures. Proc. Natl. Acad. Sci. U. S. A..

[49] Hubalek F., Binda C., Khalil A., Li M., Mattevi A., Castagnoli N. (2005). Demonstration of isoleucine 199 as a structural determinant for the selective inhibition of human monoamine oxidase B by specific reversible inhibitors. J. Biol. Chem..

[50] Rebrin I., Geha R.M., Chen K., Shih J.C. (2001). Effects of carboxyl-terminal truncations on the activity and solubility of human monoamine oxidase B. J. Biol. Chem..

[51] Bose J.L., Fey P.D., Bayles K.W. (2013). Genetic tools to enhance the study of gene function and regulation in *Staphylococcus aureus*. Appl. Environ. Microbiol..

[52] Fey P.D., Endres J.L., Yajjala V.K., Widhelm T.J., Boissy R.J., Bose J.L. (2013). A genetic resource for rapid and comprehensive phenotype screening of nonessential *Staphylococcus aureus* genes. mBio.

[53] Bae T., Schneewind O. (2006). Allelic replacement in *Staphylococcus aureus* with inducible counter-selection. Plasmid.

[54] Huynh K., Partch C.L. (2015). Analysis of protein stability and ligand interactions by thermal shift assay. Curr. Protoc. Protein Sci..

[55] Kendrick B.S., Kerwin B.A., Chang B.S., Philo J.S. (2001). Online size-exclusion high-performance liquid chromatography light scattering and differential refractometry methods to determine degree of polymer conjugation to proteins and protein-protein or protein-ligand association states. Anal. Biochem..

[56] Tarazona M.P., Saiz E. (2003). Combination of SEC/MALS experimental procedures and theoretical analysis for studying the solution properties of macromolecules. J. Biochem. Biophys. Methods.

[57] Del Vecchio K., Stahelin R.V. (2016). Using surface plasmon resonance to quantitatively assess lipid-protein interactions. Methods Mol. Biol..

[58] Delaglio F., Grzesiek S., Vuister G.W., Zhu G., Pfeifer J., Bax A. (1995). NMRPipe: a multidimensional spectral processing system based on UNIX pipes. J. Biomol. NMR.

[59] Lee W., Tonelli M., Markley J.L. (2015). NMRFAM-SPARKY: enhanced software for biomolecular NMR spectroscopy. Bioinformatics.

[60] Guntert P., Mumenthaler C., Wuthrich K. (1997). Torsion angle dynamics for NMR structure calculation with the new program DYANA. J. Mol. Biol..

[61] Shen Y., Bax A. (2015). Protein structural information derived from NMR chemical shift with the neural network program TALOS-N. Methods Mol. Biol..

[62] Olsson M.H., Sondergaard C.R., Rostkowski M., Jensen J.H. (2011). PROPKA3: consistent treatment of internal and surface residues in empirical pKa predictions. J. Chem. Theor. Comput..

[63] Humphrey W., Dalke A., Schulten K. (1996). VMD: visual molecular dynamics. J. Mol. Graph..

[64] Vermaas J.V., Baylon J.L., Arcario M.J., Muller M.P., Wu Z., Pogorelov T.V. (2015). Efficient exploration of membrane-associated phenomena at atomic resolution. J. Membr. Biol..

[65] Baylon J.L., Vermaas J.V., Muller M.P., Arcario M.J., Pogorelov T.V., Tajkhorshid E. (2016). Atomic-level description of protein-lipid interactions using an accelerated membrane model. Biochim. Biophys. Acta.

[66] Gorgun D., Lihan M., Kapoor K., Tajkhorshid E. (2021). Binding mode of SARS-CoV-2 fusion peptide to human cellular membrane. Biophys. J..

[67] Jo S., Kim T., Iyer V.G., Im W. (2008). CHARMM-GUI: a web-based graphical user interface for CHARMM. J. Comput. Chem..

[68] Qi Y., Cheng X., Lee J., Vermaas J.V., Pogorelov T.V., Tajkhorshid E. (2015). CHARMM-GUI HMMM builder for membrane simulations with the highly mobile membrane-mimetic model. Biophys. J..

[69] Wells D.B., Abramkina V., Aksimentiev A. (2007). Exploring transmembrane transport through α-hemolysin with grid-steered molecular dynamics. J. Chem. Phys..

[70] van der Spoel D., van Maaren P.J. (2006). The origin of layer structure artifacts in simulations of liquid water. J. Chem. Theor. Comput..

[71] Jorgensen W.L., Chandrasekhar J., Madura J.D., Impey R.W., Klein M.L. (1983). Comparison of simple potential functions for simulating liquid water. J. Chem. Phys..

[72] Phillips J.C., Braun R., Wang W., Gumbart J., Tajkhorshid E., Villa E. (2005). Scalable molecular dynamics with NAMD. J. Comput. Chem..

[73] Phillips J.C., Hardy D.J., Maia J.D.C., Stone J.E., Ribeiro J.V., Bernardi R.C. (2020). Scalable molecular dynamics on CPU and GPU architectures with NAMD. J. Chem. Phys..

[74] Best R.B., Zhu X., Shim J., Lopes P.E., Mittal J., Feig M. (2012). Optimization of the additive CHARMM all-atom protein force field targeting improved sampling of the backbone Φ, Ψ and side-chain χ_1_ and χ_2_ dihedral angles. J. Chem. Theor. Comput..

[75] Klauda J.B., Venable R.M., Freites J.A., O'Connor J.W., Tobias D.J., Mondragon-Ramirez C. (2010). Update of the CHARMM all-atom additive force field for lipids: validation on six lipid types. J. Phys. Chem. B.

[76] Essmann U., Perera L., Berkowitz M.L., Darden T., Lee H., Pedersen L.G. (1995). A smooth particle mesh Ewald method. J. Chem. Phys..

[77] Martyna G.J., Tobias D.J., Klein M.L. (1994). Constant pressure molecular dynamics algorithms. J. Chem. Phys..

[78] Ryckaert J.P.C.G., Berendsen H.J.C. (1977). Numerical integration of the cartesian equations of motion of a system with constraints: molecular dynamics of *n*-alkanes. J. Comput. Phys..

[79] Miyamoto S., Kollman P.A. (1992). Settle: an analytical version of the SHAKE and RATTLE algorithm for rigid water models. J. Comput. Chem..

[80] Punjani A., Rubinstein J.L., Fleet D.J., Brubaker M.A. (2017). cryoSPARC: algorithms for rapid unsupervised cryo-EM structure determination. Nat. Methods.

[81] Punjani A., Fleet D.J. (2021). 3D variability analysis: resolving continuous flexibility and discrete heterogeneity from single particle cryo-EM. J. Struct. Biol..

[82] Sanchez-Garcia R., Gomez-Blanco J., Cuervo A., Carazo J.M., Sorzano C.O.S., Vargas J. (2021). DeepEMhancer: a deep learning solution for cryo-EM volume post-processing. Commun. Biol..

[83] Heymann J.B. (2018). Guidelines for using Bsoft for high resolution reconstruction and validation of biomolecular structures from electron micrographs. Protein Sci..

[84] Afonine P.V., Klaholz B.P., Moriarty N.W., Poon B.K., Sobolev O.V., Terwilliger T.C. (2018). New tools for the analysis and validation of cryo-EM maps and atomic models. Acta Crystallogr. D Struct. Biol..

[85] Williams C.J., Headd J.J., Moriarty N.W., Prisant M.G., Videau L.L., Deis L.N. (2018). MolProbity: more and better reference data for improved all-atom structure validation. Protein Sci..

[86] Meng E.C., Goddard T.D., Pettersen E.F., Couch G.S., Pearson Z.J., Morris J.H. (2023). UCSF ChimeraX: tools for structure building and analysis. Protein Sci..

[87] DeLano W.L. (2002).

