# Supplementary material for: The carboxy terminus causes interfacial assembly of oleate hydratase on a membrane bilayer
Source: J Biol Chem. 2024 Jan 10;300(2):105627. doi: 10.1016/j.jbc.2024.105627 (PMC10847778; doi:10.1016/j.jbc.2024.105627)
Supplement: Supporting Figures S1–S21 [file mmc1.docx]

**Supplemental Figures**

**The carboxy terminus causes interfacial assembly of oleate hydratase on a membrane bilayer**

Christopher D. Radka, Christy R. Grace, Hale S. Hasdemir, Yupeng Li, Carlos C. Rodriguez, Patrick Rodrigues, Michael L. Oldham, M. Zuhaib Qayyum, Aaron Pitre, William J. MacCain, Ravi C. Kalathur, Emad Tajkhorshid, and Charles O. Rock


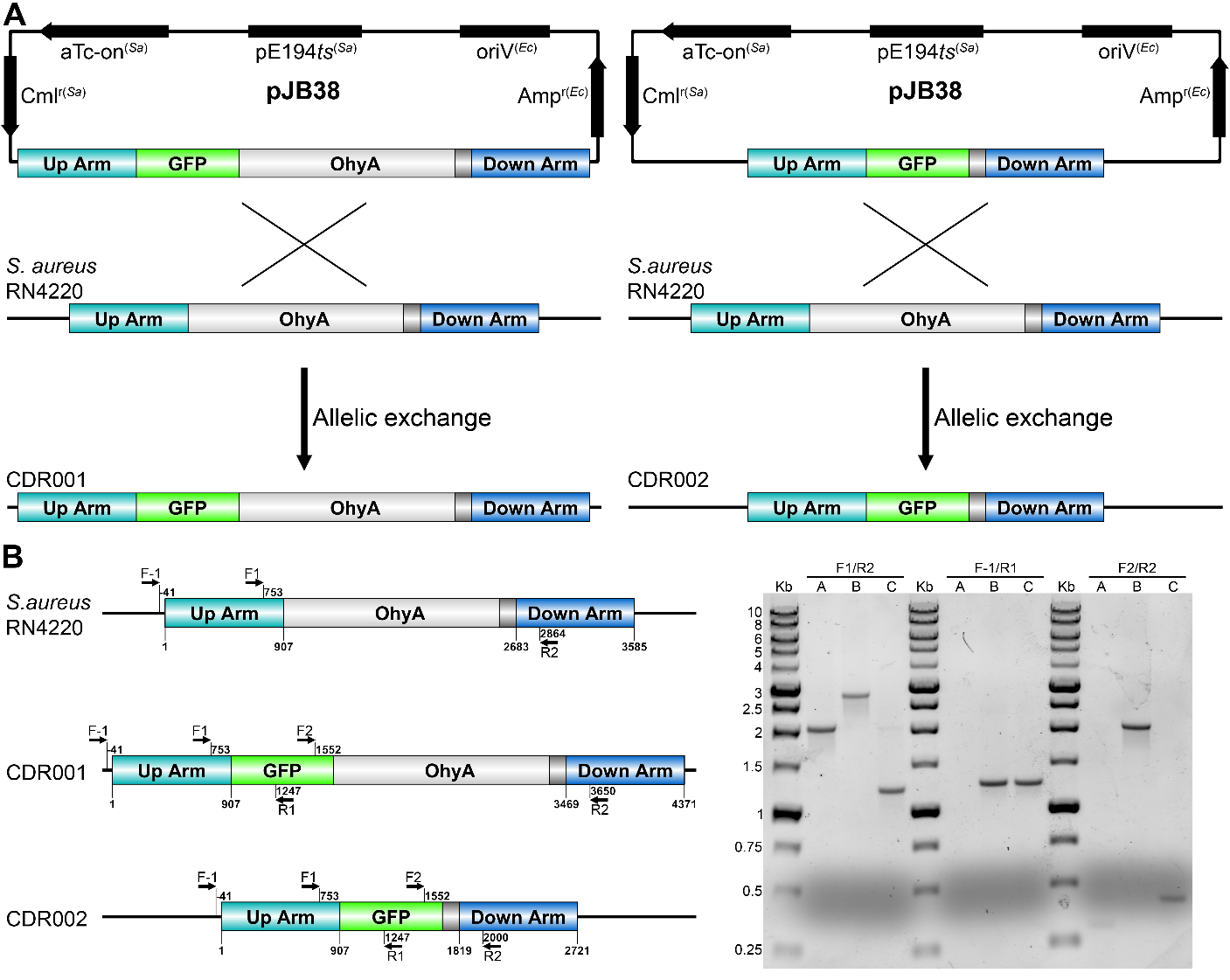


**Supplemental Figure 1. Construction of *Staphylococcus aureus* RN4220 strains CDR001 and CDR002.** Genetic knock-in strategy using the pJB38 shuttle plasmid. *A*, The pJB38 plasmid was designed to contain the genomic region surrounding the *ohyA* locus (Up Arm, *teal*; Down Arm, *blue*) and an open reading frame for integration. The open reading frame contained green fluorescent protein gene (GFP, *green*) fused to the *ohyA* gene (OhyA, light grey; OhyA carboxy terminus, dark grey) or GFP fused to the OhyA carboxy terminus. The integration step occurs by allelic exchange from the two homologous regions. The pJB38 plasmid contains an *Escherichia coli* origin of replication (oriV^(^*^Ec^*^)^) and selection marker (Amp^r(^*^Ec^*^)^), and an *S. aureus* selection marker (Cml^r(^*^Sa^*^)^) for cloning. An *S. aureus* temperature-sensitive replicon (pE194*ts*^(^*^Sa^*^)^) and counterselection marker (aTc-on^(^*^Sa^*^)^) are for genetic manipulation, screening, and curing the plasmid. *B*, Verification of gene knock-ins. *Left panel*, genomic structure at the *ohyA* locus in RN4220, CDR001, or CDR002. Primers were synthesized at the positions denoted by arrows for screening genomic DNA, and the nucleotide position of each primer is indicated. Primers R1 and F2 are in the GFP region and noncomplementary to RN4220 genomic DNA. *Right panel*, PCR confirmation of strains using genomic DNA from RN4220 (A), CDR001 (B), and CDR002 (C), and primer pairs F1/R2, F-1/R1, and F2/R2.


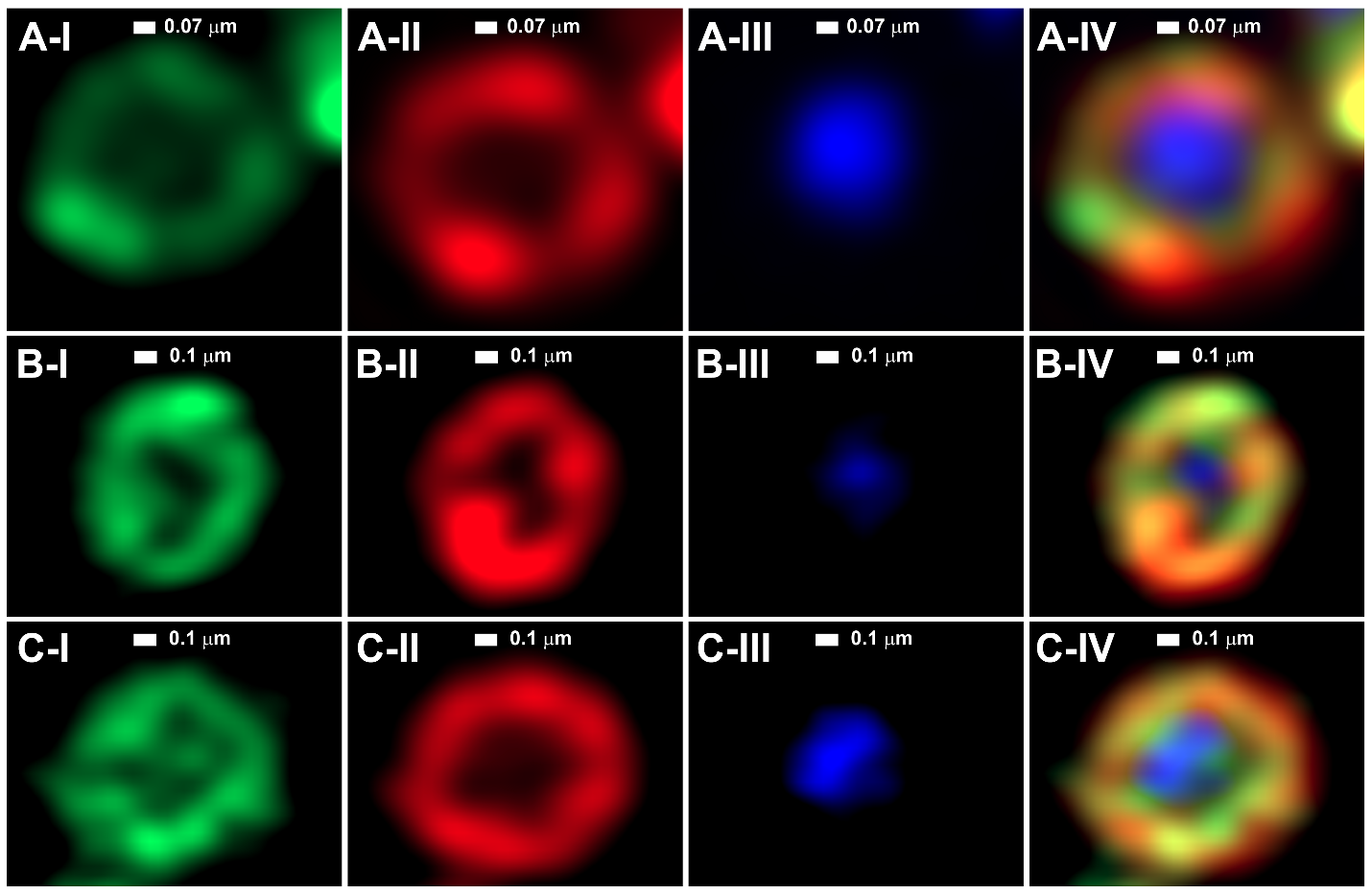


**Supplemental Figure 2. Super resolution microscopy of *S. aureus* RN4220 strain CDR001 cells** **engineered to express GFP-OhyA.** CDR001 cells were labeled with DAPI, to stain the nucleoid and visualize the intracellular compartment, and CellBrite® Fix 640, to stain and visualize the membrane compartment. *A–C*, Snapshots of representative CDR001 cells using a 488 laser (I) to detect GFP-OhyA, 639 laser (II) to detect CellBrite® Fix 640, 405 laser (III) to detect DAPI, or a merge (IV) of the three images. Scale bars are provided for each image that correspond to 0.07–0.1 μm.


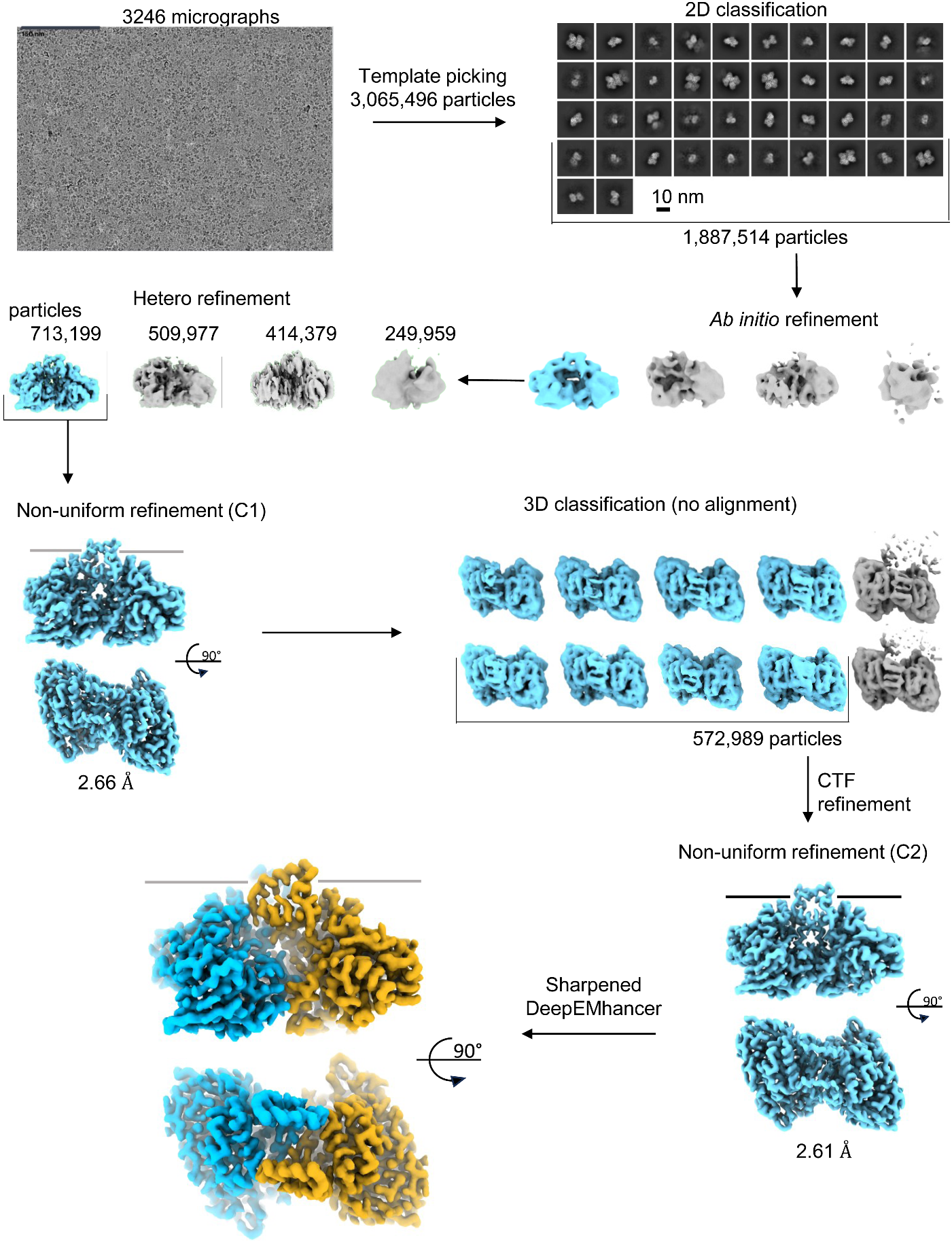


**Supplemental Figure 3. Cryo-EM data processing for the OhyA dimer.** Initial satisfactory dimer reconstructions are colored in cyan. The representative scale bar for the 2D class average images indicates 10 nm. The final sharpened reconstruction is colored for each OhyA subunit.


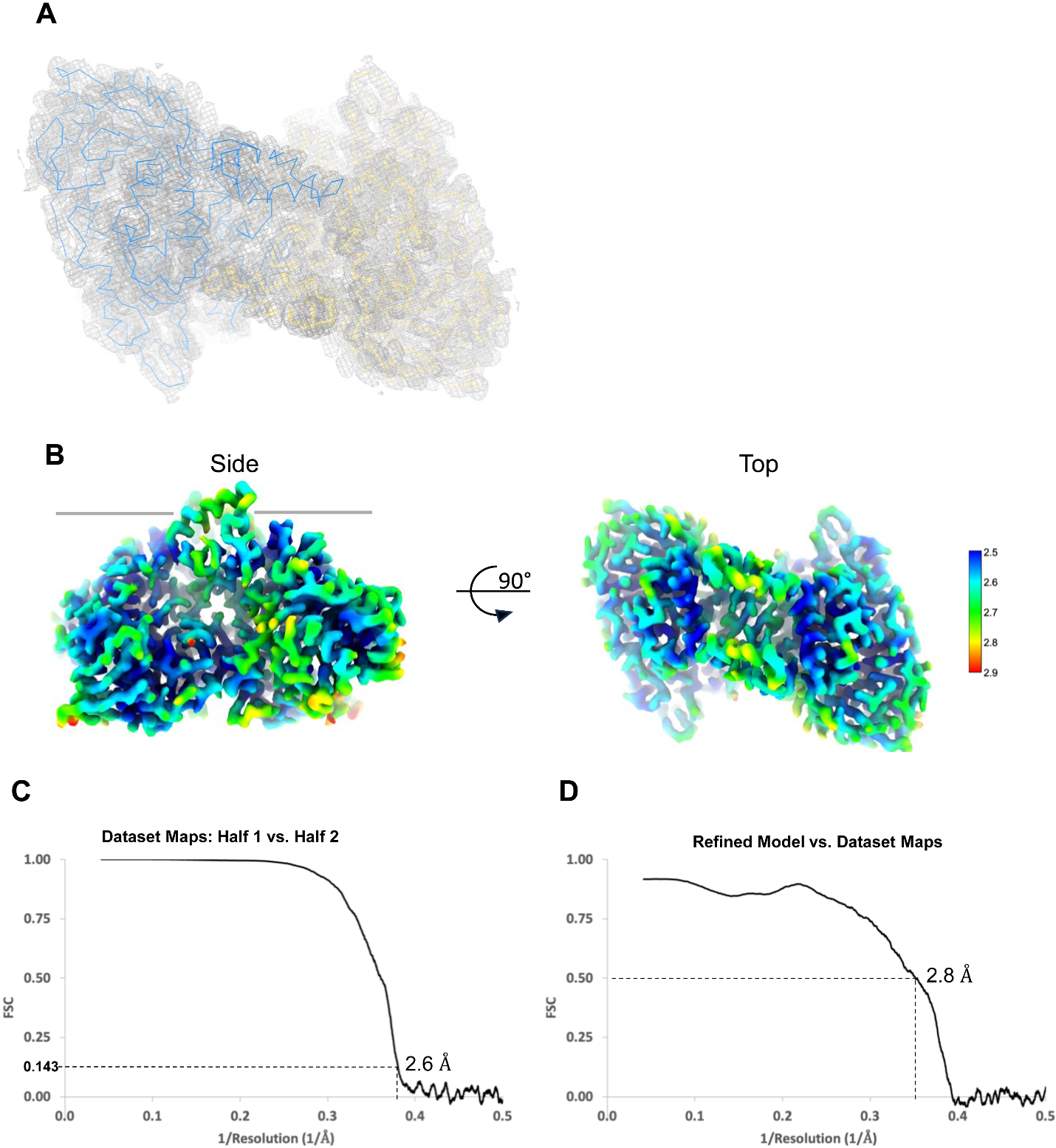


**Supplemental Figure 4. Cryo-EM map properties for the OhyA dimer.** *A*, Backbone of the refined dimer structure placed into the sharpened map. *B*, Sharpened map colored by estimated local resolution (Blocres). *C*, Fourier shell correlation (FSC) between the two half maps for the 2.61 Å reconstruction. *D*, FSC between the refined structure and the full map.


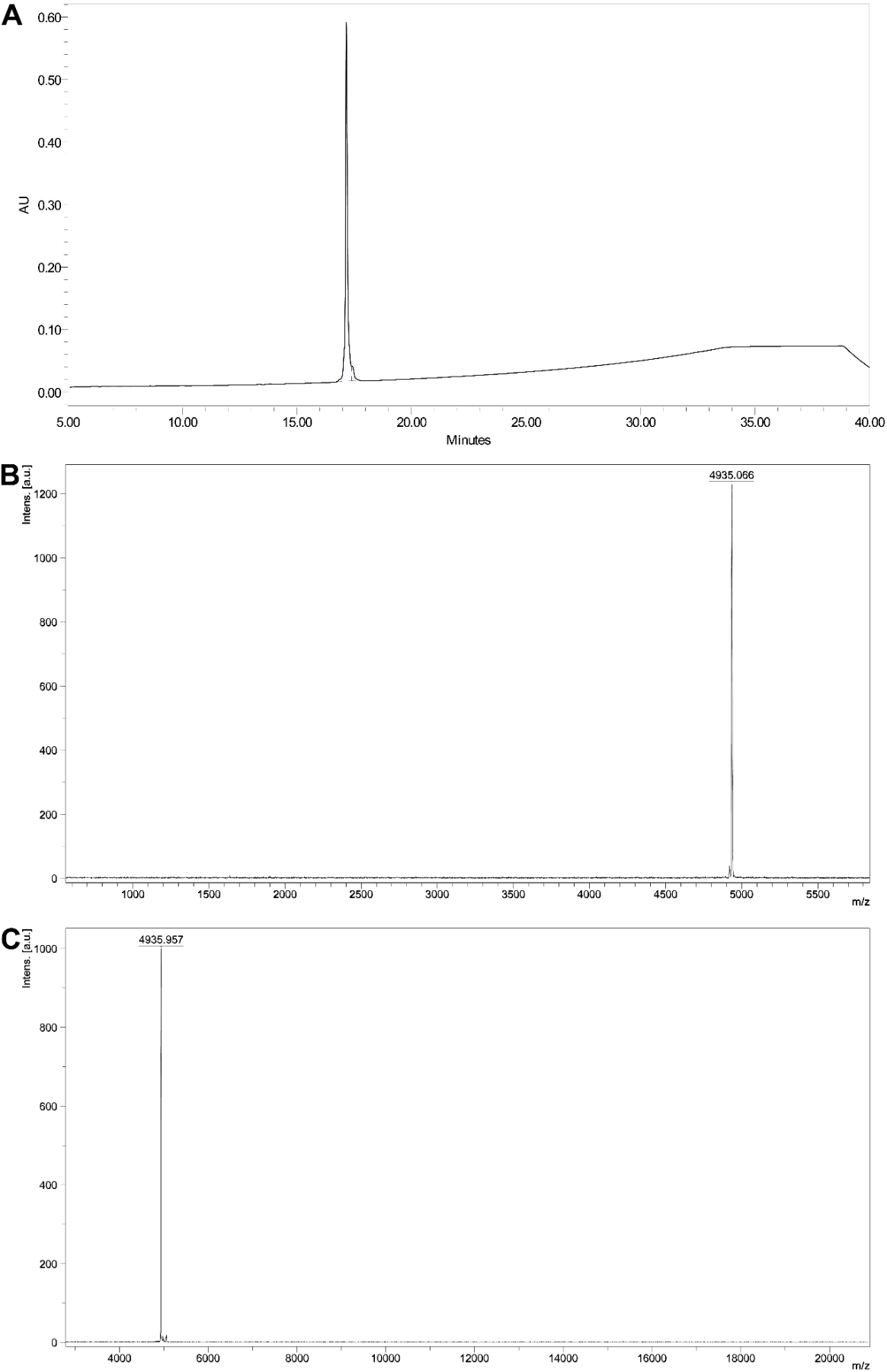


**Supplemental Figure 5. OhyA CTD peptide(550-591) preparation.** *A*, HPLC chromatogram of the OhyA CTD peptide(550-591) purification after chemical synthesis. The OhyA CTD peptide(550-591) eluted at 17.16 min. *B–C*, Mass spectrometry scans of the purified peptide show a single species at the expected mass for OhyA CTD peptide(550-591).


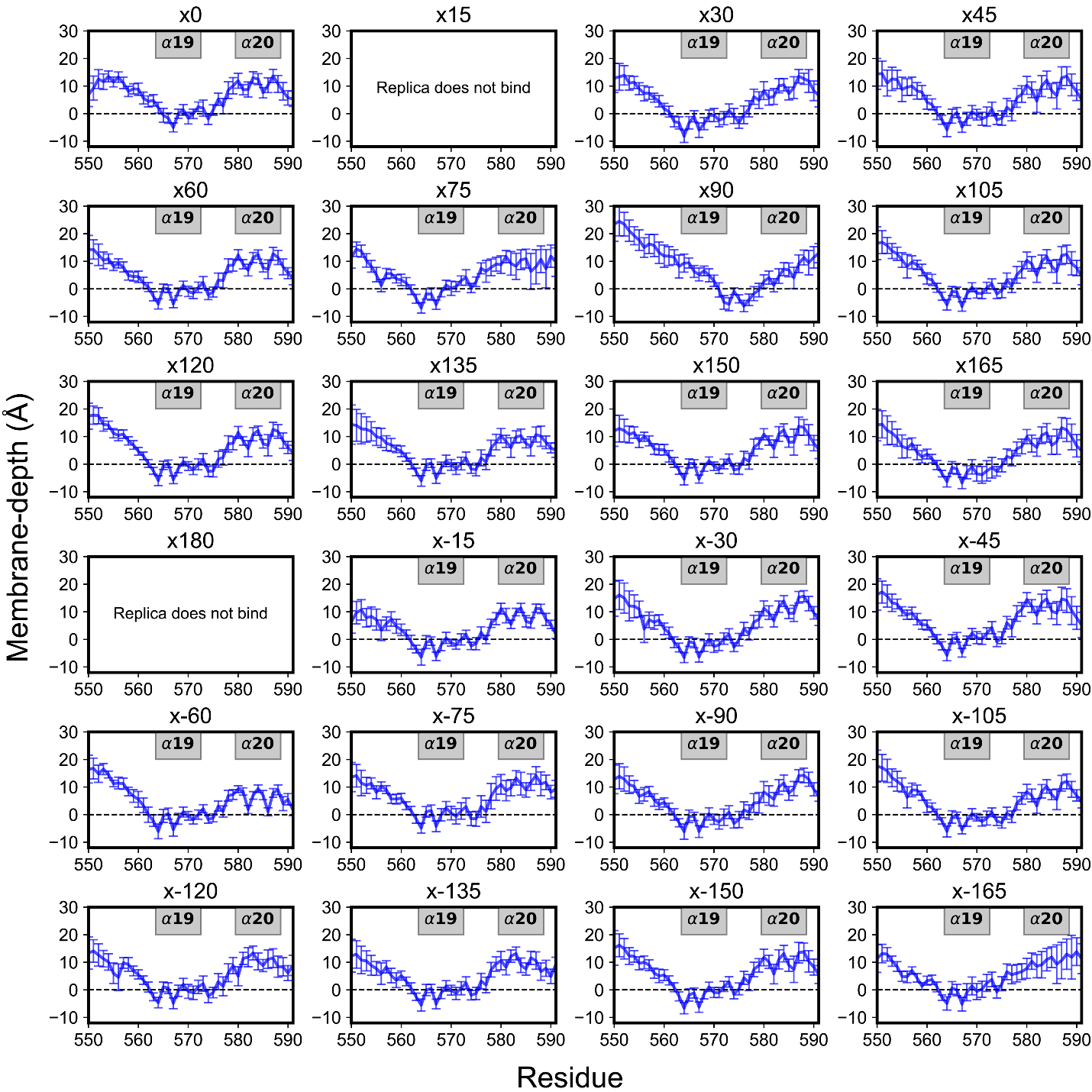


**Supplemental Figure 6. Membrane-bound conformation of CTD fragment in each OhyA CTD peptide(550-591)•HMMM replica.** The OhyA CTD peptide(550-591) was rotated in 15° increments to sample various starting conformations in binding simulations. Ensemble-averaged closest distance of each amino acid residue (heavy atoms only) to the membrane phosphate layer along the membrane normal (z) axis were calculated over the membrane-bound frames for each replica (see *Experimental Procedures*). Annotations indicate residue positions that correspond to helices α19 and α20.

**
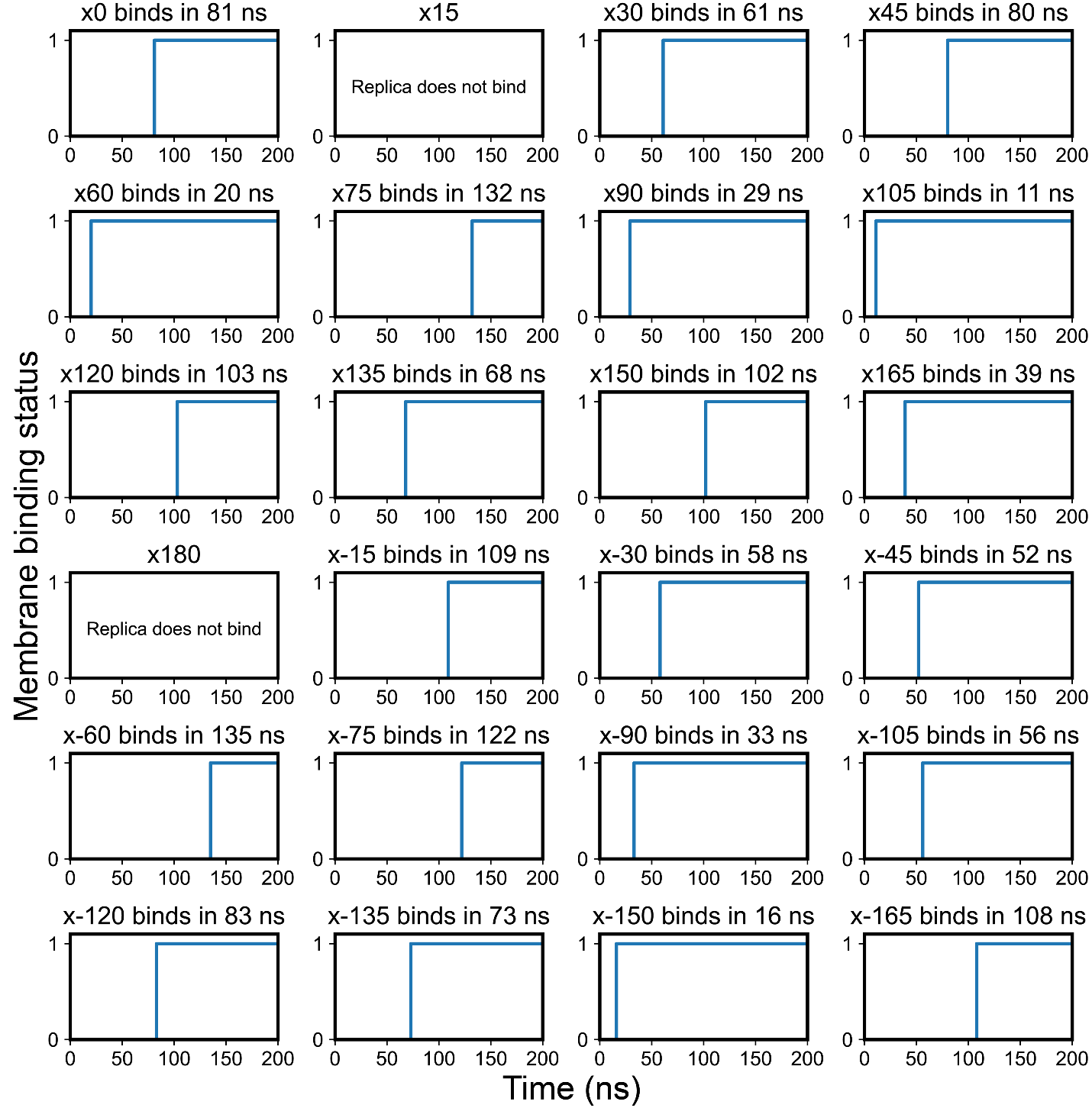
**

**Supplemental Figure 7. Analysis of OhyA CTD peptide(550-591) membrane binding in HMMM replicas.** Each frame of the HMMM simulation is evaluated for its binding status. A frame is deemed ‘membrane-bound’ when a heavy-atom inserts at least 3 Å beneath the membrane phosphate plane for a minimum of 30 ns. Within this span, a deviation of up to 3 frames that don't meet the insertion criteria is permissible. The figure displays the membrane-bound status for each frame of all HMMM replicas, highlighting the time taken for the OhyA CTD peptide to stably bind to the membrane. Replicas x15 and x180 do not exhibit any membrane-bound frames as per the defined criteria.

**
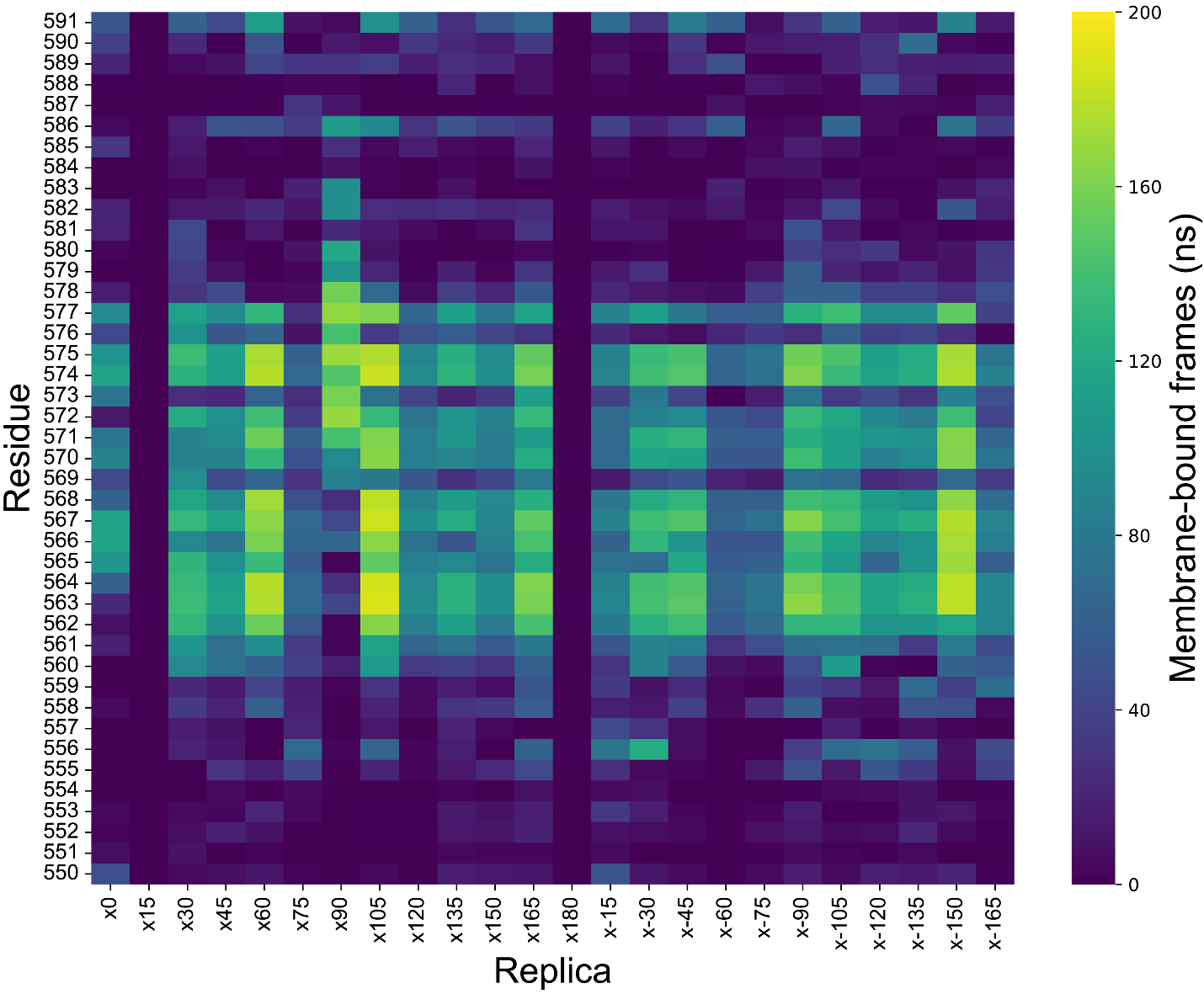
**

**Supplemental Figure 8. Helix α19 residues in OhyA CTD peptide(550-591) exhibit frequent interactions with the membrane.**  The heatmap highlights the contact frequency between each residue and PG lipids across replicas using membrane-bound frames from the HMMM simulations. Contact between a residue and PG is defined when a residue heavy atom is within 3.5 Å of a PG heavy atom.

**
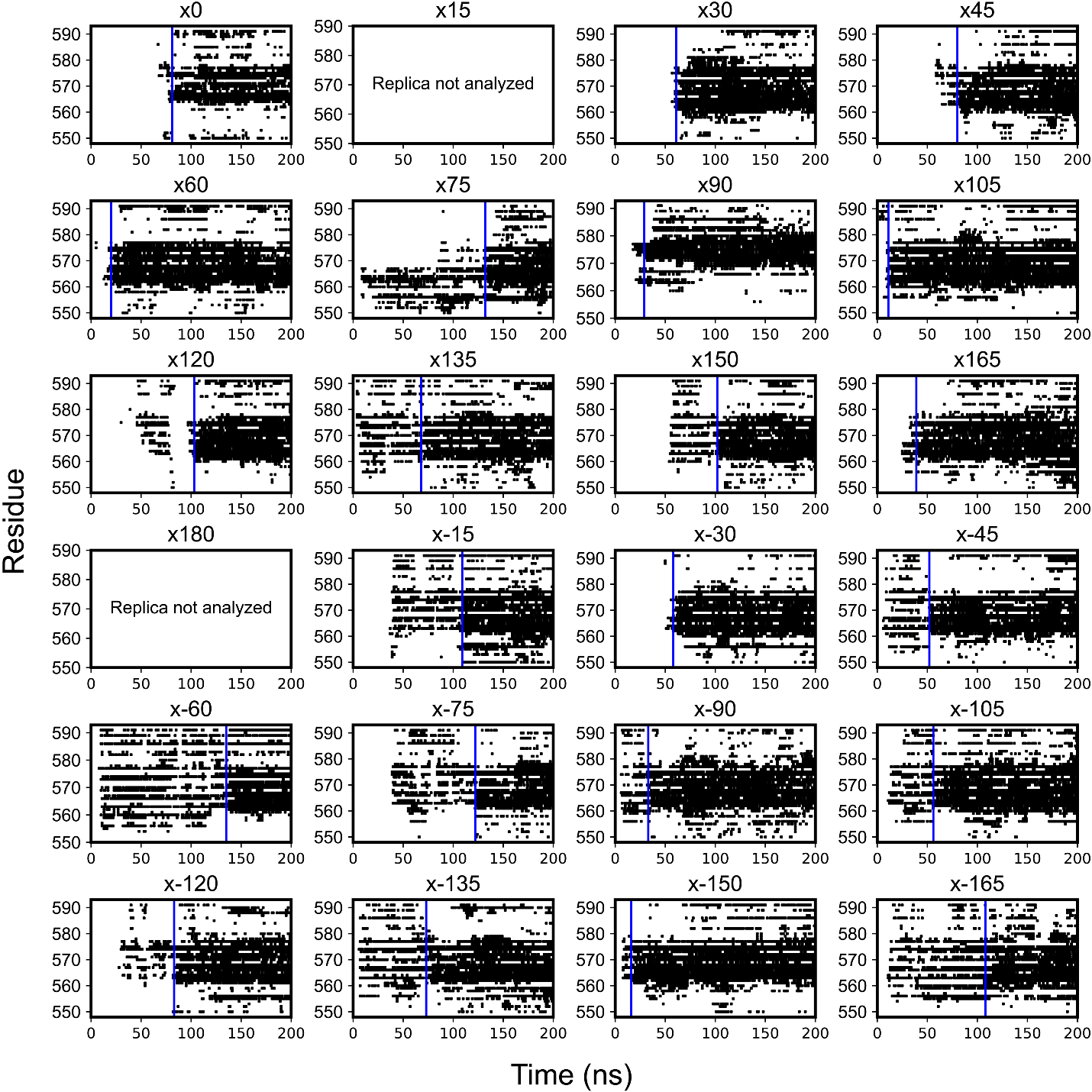
**

**Supplemental Figure 9. Temporal protein-lipid interaction analysis of OhyA CTD peptide(550-591)**•**HMMM simulations.** Frames from membrane-binding simulations are shown where residues are in contact with phosphatidylglycerol (PG) from each HMMM replica using a 3.5 Å heavy atom distance contact cut-off between the OhyA peptide and PG lipids. A vertical line (*blue*) shows when the system is initially considered membrane-bound (see *Experimental* *Procedures*).

**
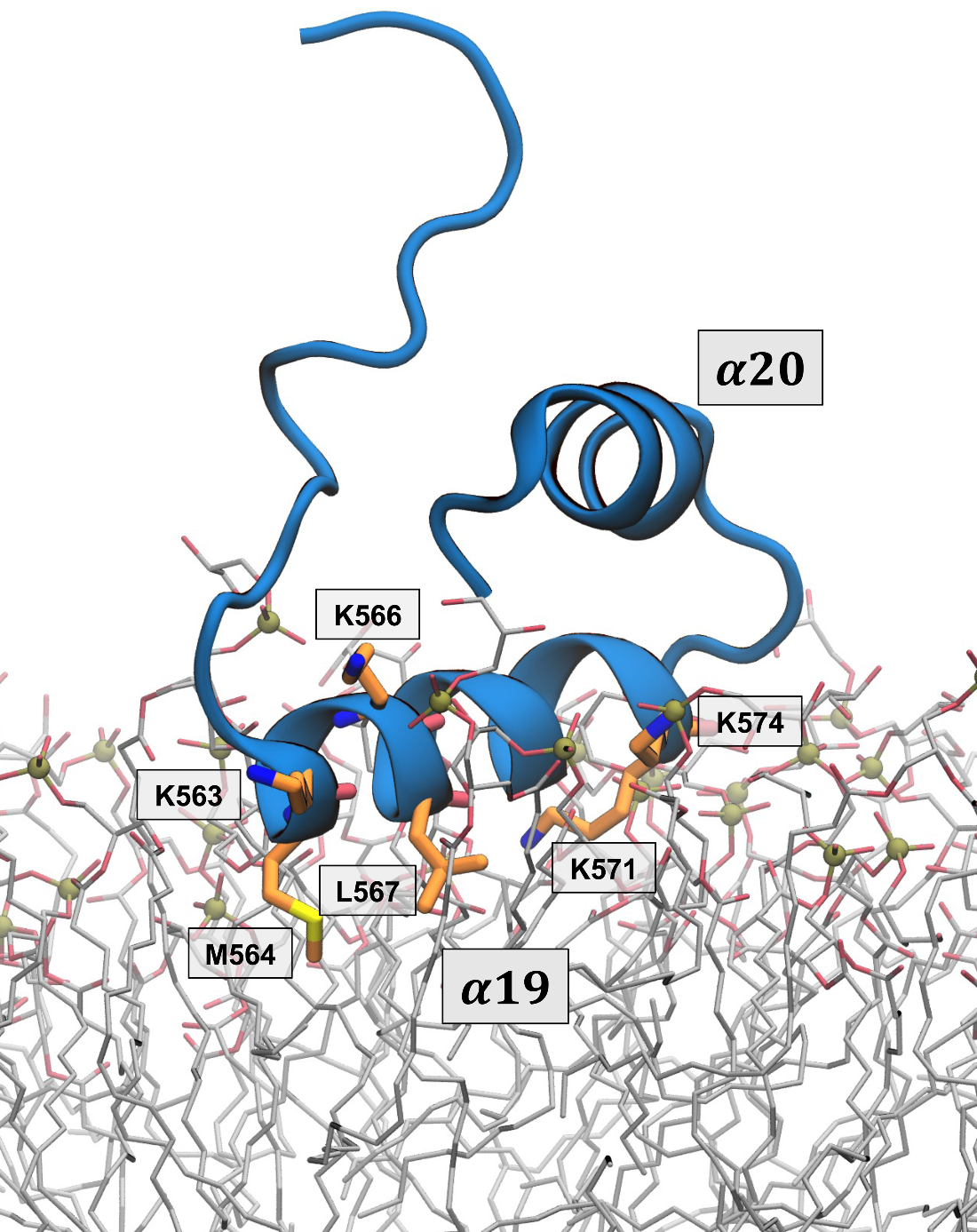
**

**Supplemental Figure 10. A representative snapshot of the OhyA CTD peptide(550-591)•FM system.** Key residues from helix α19 that are important for membrane binding are represented as sticks with carbon atoms in orange, oxygen atoms in red, nitrogen atoms in blue, and sulfur atoms in yellow. DOPG lipids are represented in licorice style, with carbon atoms in gray, oxygen in red, and phosphorus in tan spheres.


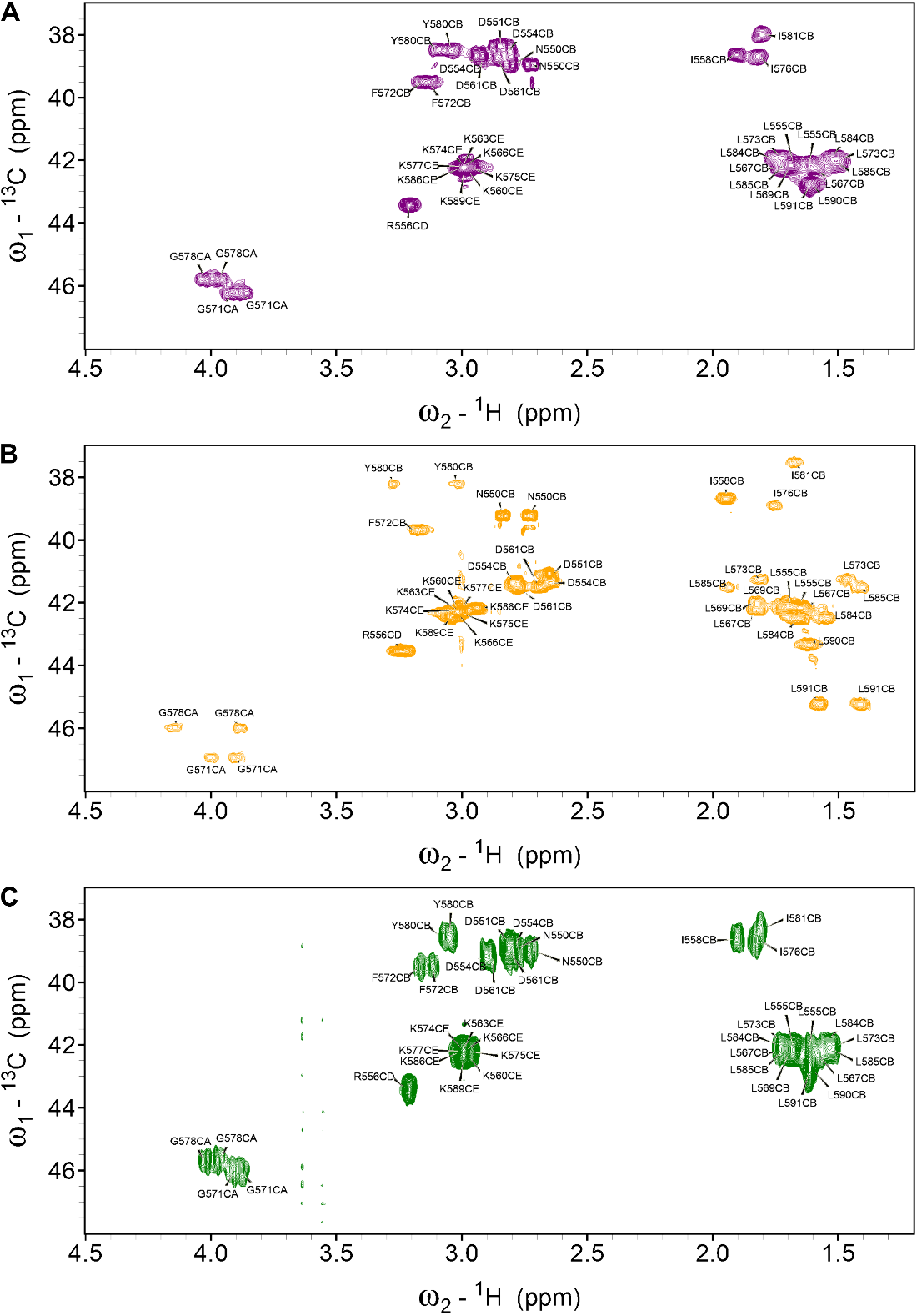


**Supplemental Figure 11. 2D[^13^C,^1^H] HSQC spectra of OhyA CTD peptide(550-591).** Region of 2D[^13^C,^1^H] HSQC spectra of OhyA CTD peptide(550-591) in (*A*) 150 mM NaCl, (*B*) 50 mM K_2_HPO_4_, or (b) 1% Glycerol.


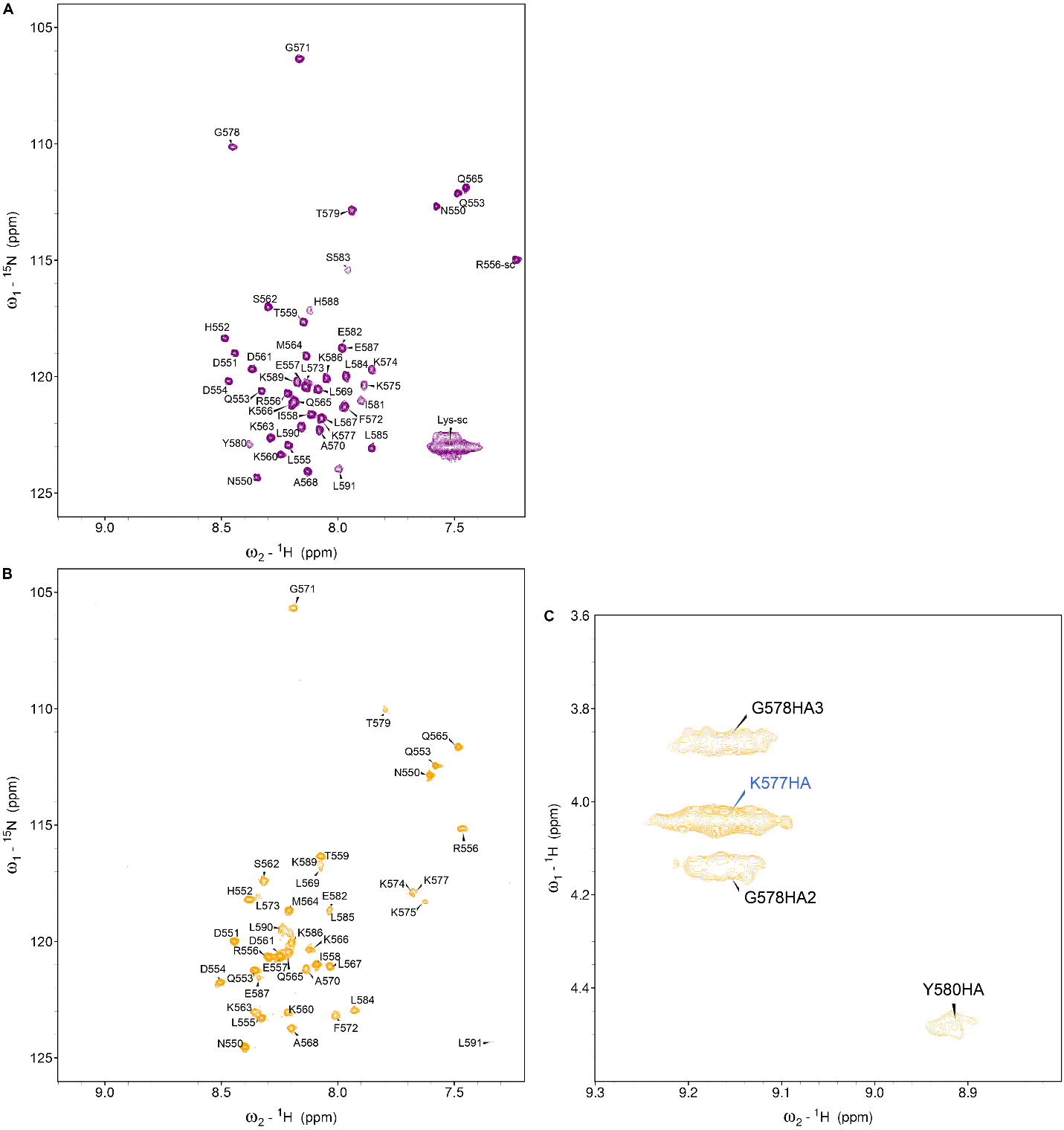
**Supplemental Figure 12. 2D[^15^N,^1^H] HSQC spectra of OhyA CTD peptide(550-591).** Region of 2D[^15^N,^1^H] HSQC spectra of OhyA CTD peptide(550-591) in (*A*) 150 mM NaCl or (*B*) 50 mM K_2_HPO_4_. Lysine side chain Nz-Hz peaks appear broad and Ne-He of R556 are marked. *C*, Region of the 2D NOESY spectrum in 50 mM K_2_HPO_4_ showing the broad upfield shifted G578 and Y580 resonances. The amide N-H resonance is missing for these two residues in (*B*).

**
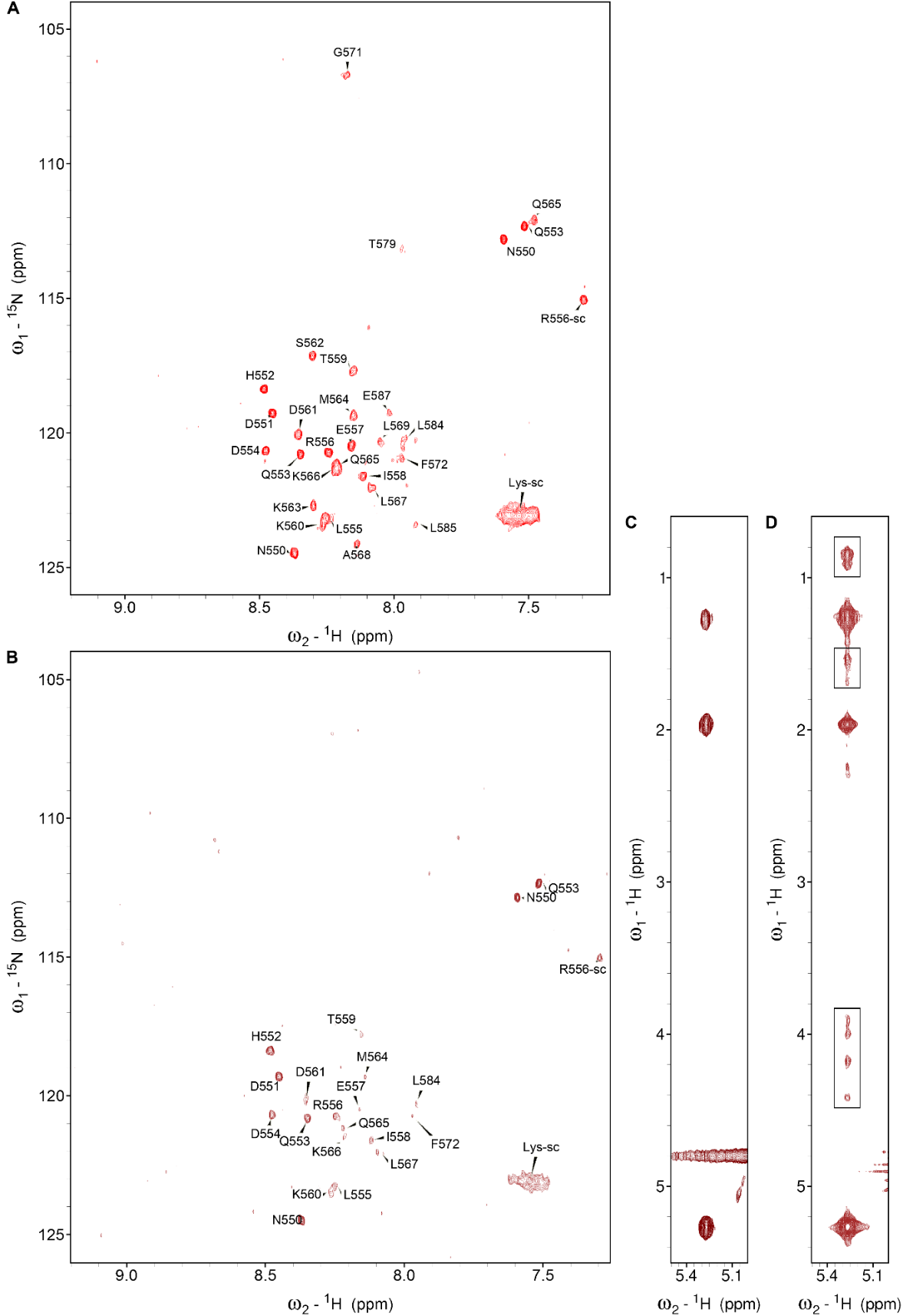
**

**Supplemental Figure 13. 2D[^15^N,^1^H] HSQC spectra of OhyA CTD peptide(550-591) with DOPG liposome.** Region of 2D [^15^N, ^1^H] HSQC spectra of OhyA CTD peptide(550-591), with (*A*) 0.2 mM or (*B*) 0.5 mM DOPG liposome. At 0.5 mM DOPG liposome, most of the peaks have disappeared indicative of the peptide association with the liposome. Intermolecular NOE’s (shown in boxes) observed from DOPG liposome (0.5 mM) to the peptide. 2D-TOCSY (*C*) and 2D-NOESY (*D*) spectra of peptide with 0.5 mM DOPG liposome sample. Black boxes frame intermolecular NOE’s observed in the 2D-NOESY spectra from DOPG liposome to the peptide.

**
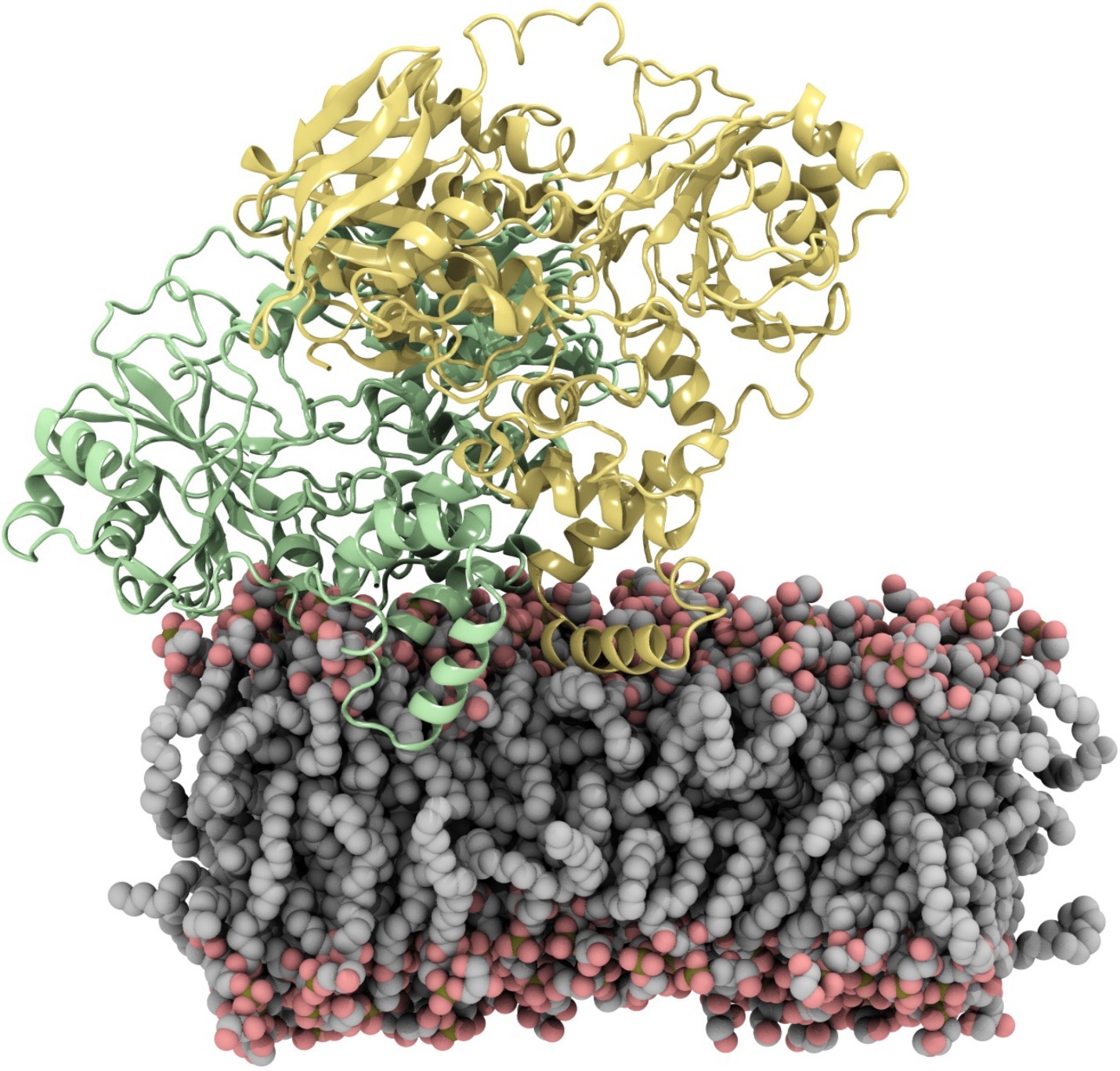
** **Supplemental** **Figure 14. Steric clashes resulting after the direct superimposition of dimer CTD onto the OhyA CTD peptide.** OhyA protomers are colored yellow and green. DOPG membrane is shown by van der Waals representation with carbon in grey, oxygen in red, and phosphorus in tan.

**
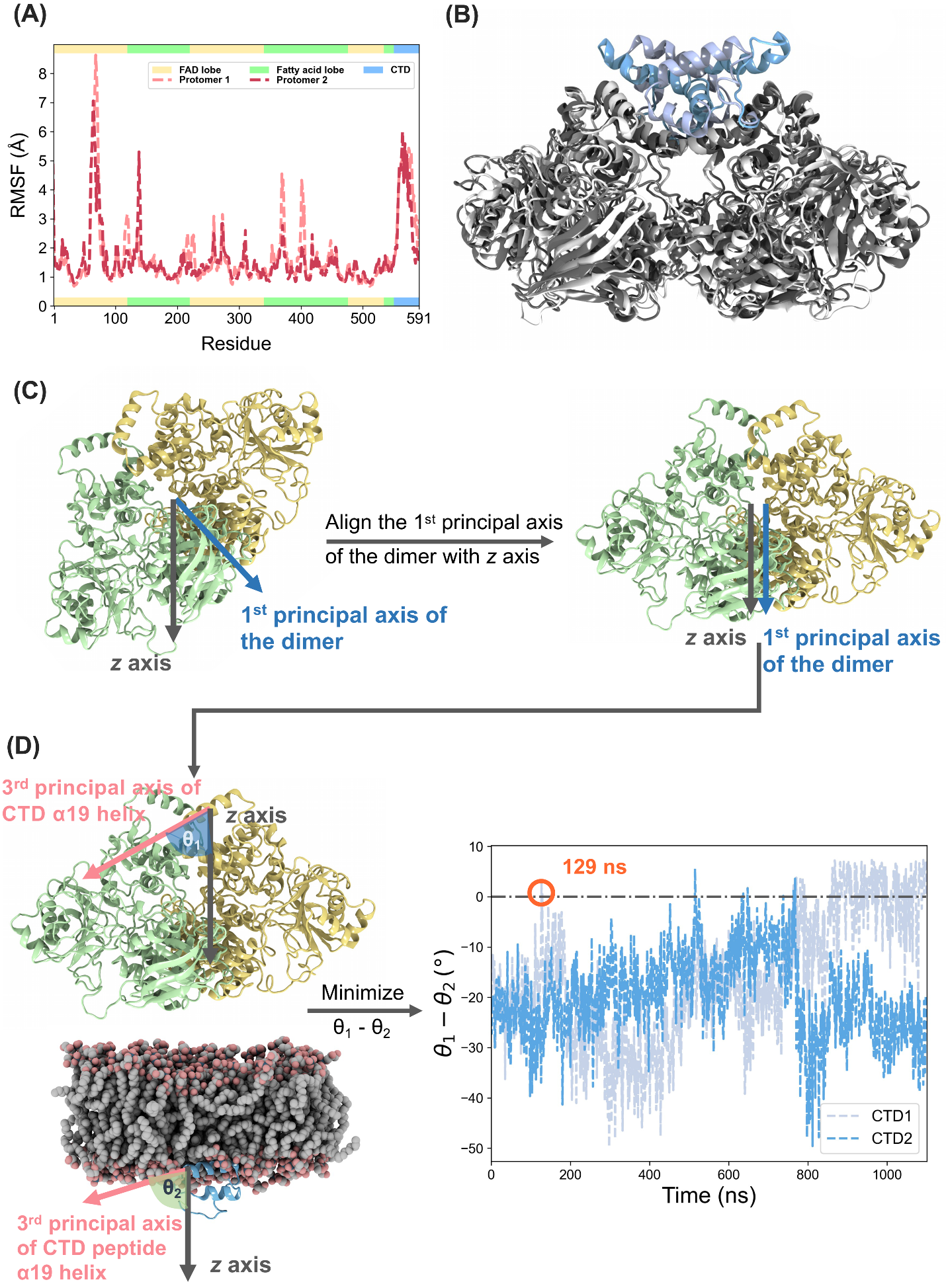
** **Supplemental** **Figure 15. Workflow for selecting dimer conformation for modeling OhyA in the membrane.** *A*, RMSF of OhyA dimer in the water box simulation shows flexibility of the CTD region. *B*, The conformational comparison of the CTD region between the start (CTD in light blue and the rest of the protein in grey) and end (CTD in dark blue and the rest of the protein in white) of the water box simulation. The CTD helix α19 converges to a more horizontal conformation by the end of the water box simulation. *C*–*D*, Steps to select a relaxed OhyA dimer conformation for modeling the membrane-bound OhyA dimer. *C*, Alignment of the first principal axis of the dimer to z axis at each frame of the OhyA water box simulation. OhyA protomers are colored yellow and green. *D*, Calculating the conformational difference between α19 of the dimer CTD and CTD peptide. OhyA protomers are colored yellow and green, while CTD peptide is blue. DOPG membrane is shown by van der Waals representation with carbon in grey, oxygen in red, and phosphorus in tan. The angle θ_1_ between the third principal axis of α19 of the dimer and the z axis was measured throughout the water box simulation. The angle θ_2_ between the third principal axis of the α19 of the peptide and z axis was measured at the last frame of OhyA CTD peptide(550-591)•FM simulation. The first frame (129 ns) from the water box simulation which minimizes the θ_1_-θ_2_ difference was selected to model the membrane-bound OhyA dimer.

**
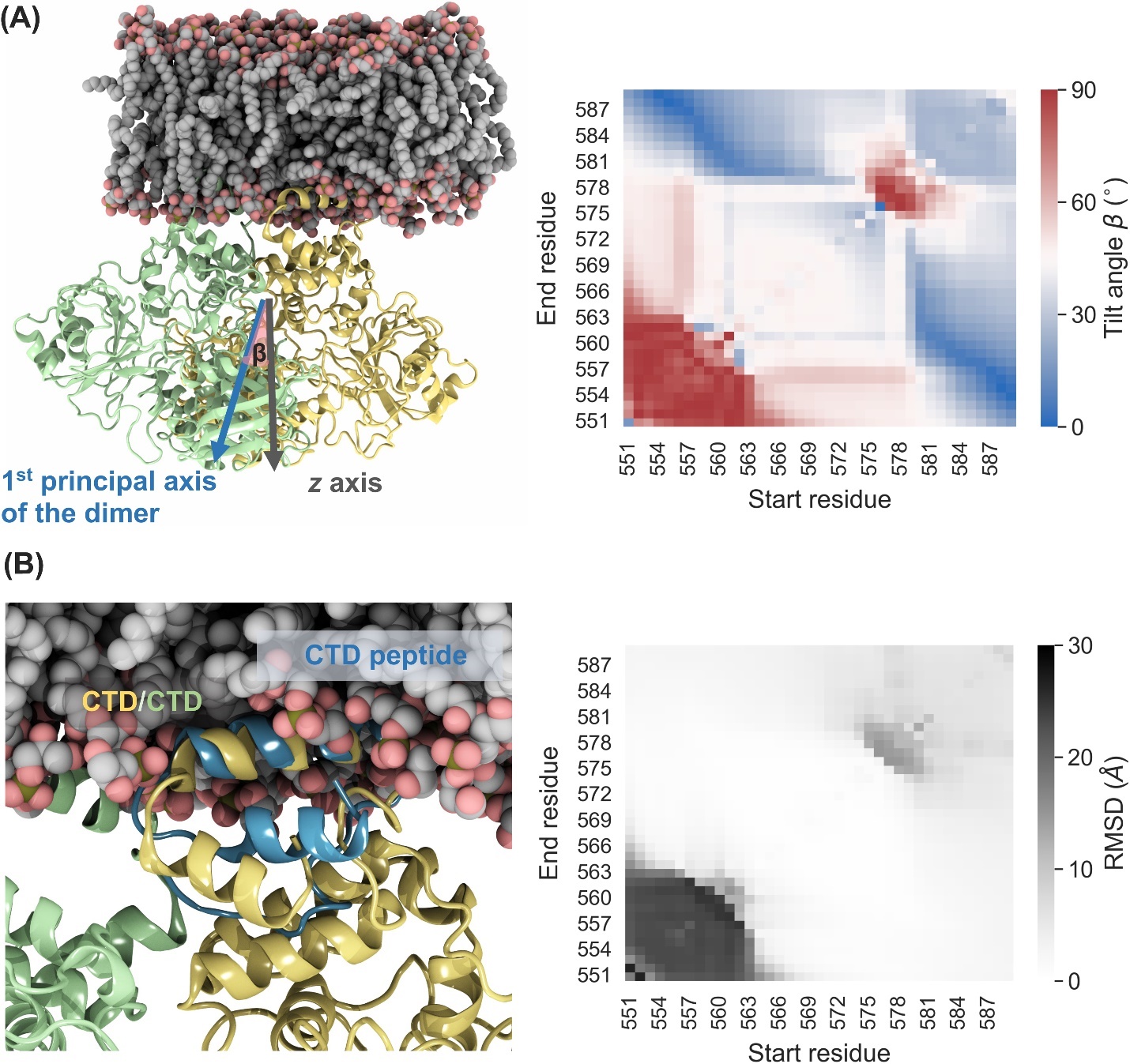
** **Supplemental** **Figure 16. Optimization of alignment region to superimpose OhyA dimer onto membrane-bound CTD peptide.** OhyA protomers are colored yellow and green, while CTD peptide is blue. DOPG membrane is shown by van der Waals representation with carbon in grey, oxygen in red, and phosphorus in tan. The heatmap entries in the i-th row and j-th column (with i < j) denote the chosen metric value (β or RMSD) obtained after aligning the OhyA protomer CTD to the FM-bound OhyA CTD peptide using the backbone atoms from residue number i to j. *A*, Calculation of the tilt angle of the OhyA dimer after superimposition onto the FM-bound CTD peptide. The tilt angle, β, is defined as the angle between the first principal axis of the dimer and the membrane normal. The heatmap shows the tilting degree of OhyA dimer after the superimposition using the selected region. *B*, Calculation of the RMSD between α19 of the aligned OhyA dimer and CTD peptide. An alignment region with a small β and RMSD was selected to model the membrane-bound OhyA dimer (see *Experimental Procedures*).

**
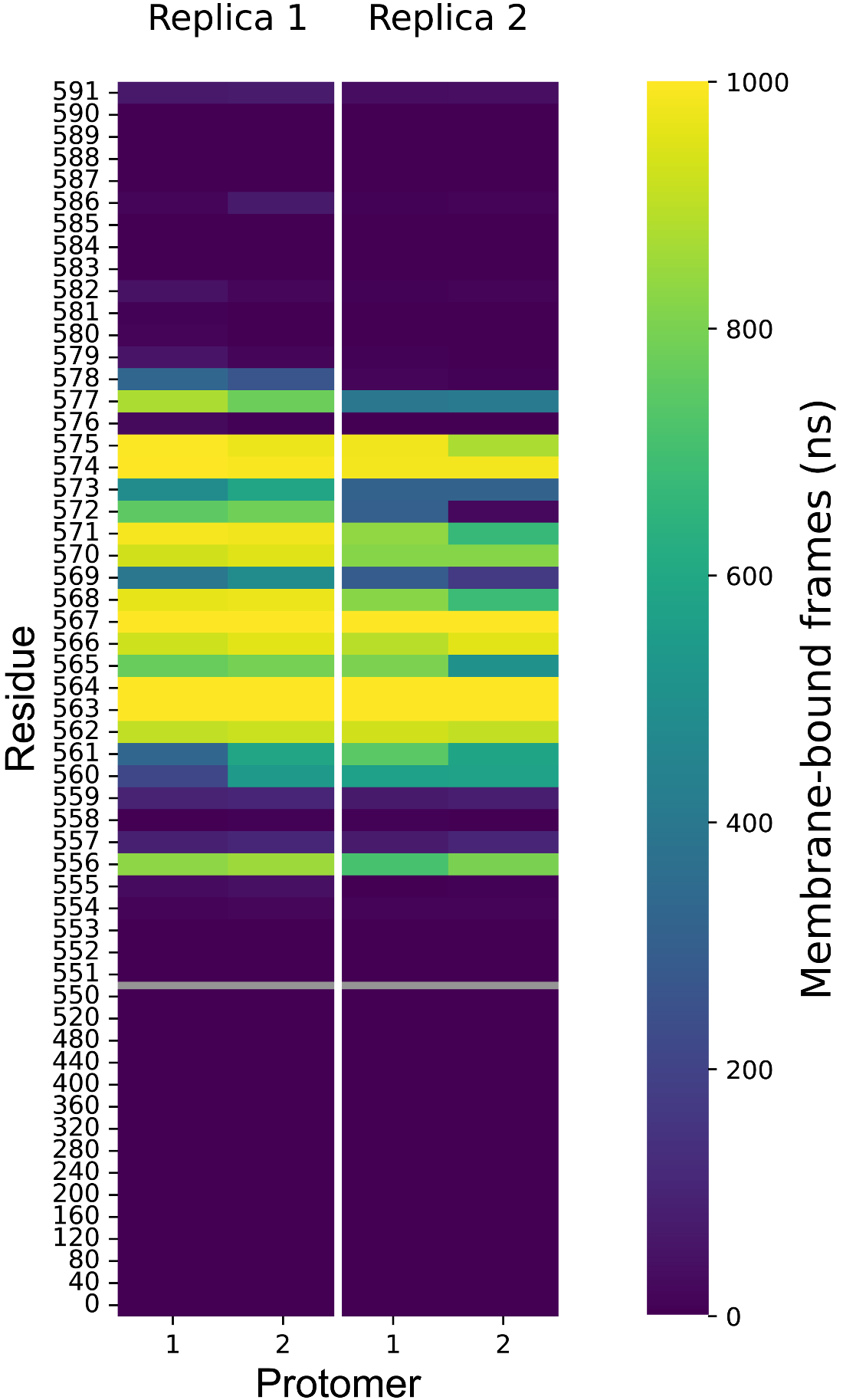
**

**Supplemental Figure 17. Residue-phosphatidylglycerol (PG) contact heatmap for the membrane-bound OhyA dimer simulations.** Heatmap of PG contact data for each residue in the OhyA dimer using a 3.5 Å contact cut-off to PG, showing two simulation replicas.


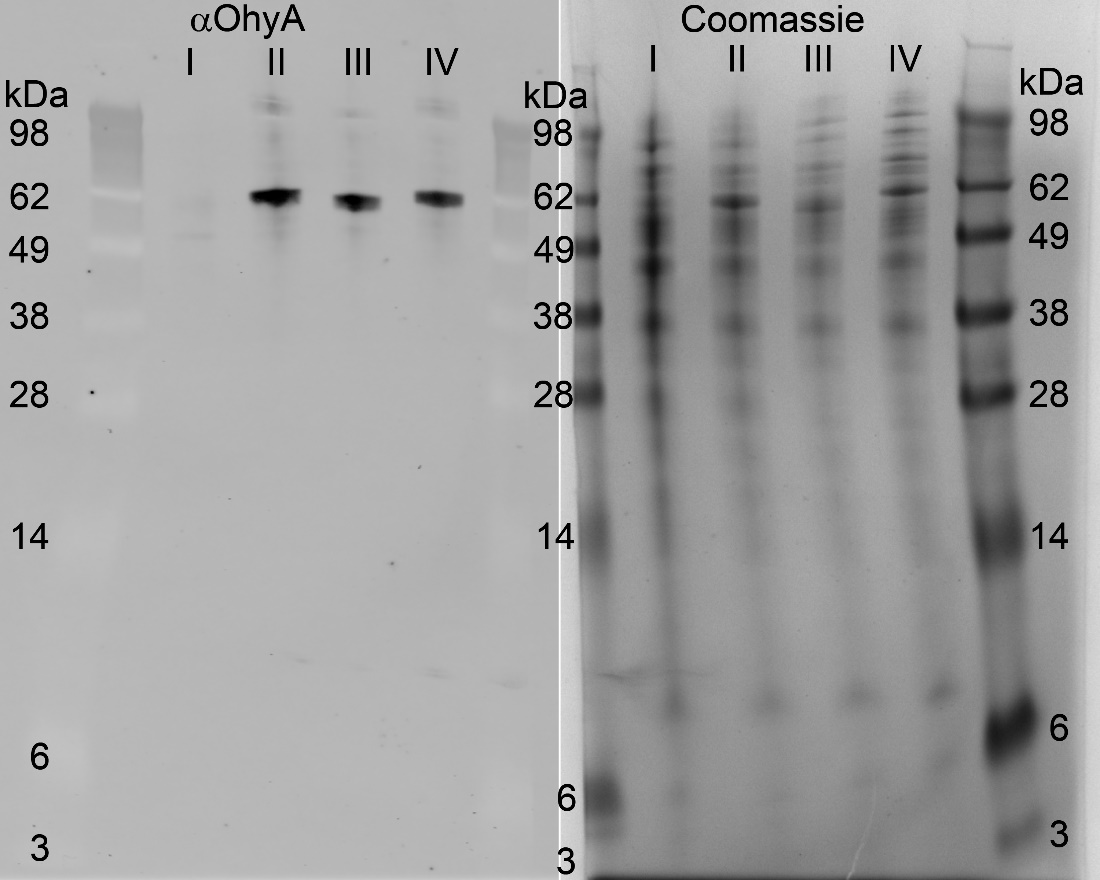


**Supplemental Figure 18. Confirmation of OhyA abundance in *S. aureus* cells.** Analysis of cell lysates from plasmid-bearing Δ*ohyA* cells expressed nothing (I), OhyA (II), OhyA(ΔHTH) (III), or OhyA(MD) (IV). *Left panel*, Immunoblot using polyclonal OhyA antibodies. *Right panel*, SDS-PAGE and Coomassie staining showing equal sample loading.


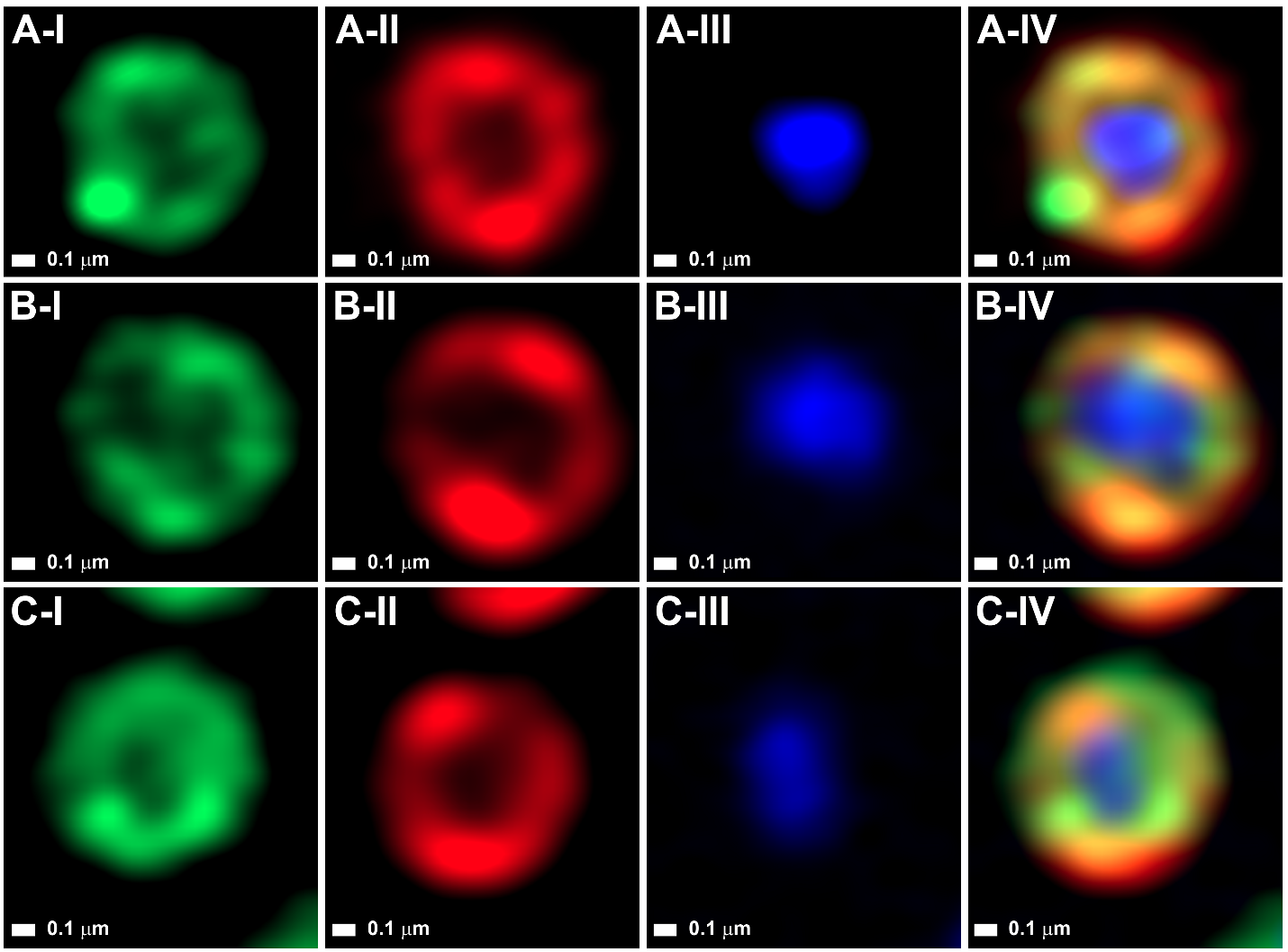


**Supplemental Figure 19. Super resolution microscopy of *S. aureus* RN4220 strain CDR002 cells engineered to express GFP-CTD.** CDR002 cells were labeled with DAPI, to stain the nucleoid and visualize the intracellular compartment, and CellBrite® Fix 640, to stain and visualize the membrane compartment. *A–C*, Snapshots of representative CDR002 cells using a 488 laser (I) to detect GFP-CTD, 639 laser (II) to detect CellBrite® Fix 640, 405 laser (III) to detect DAPI, or a merge (IV) of the three images. Scale bars are provided for each image that correspond to 0.1 μm.


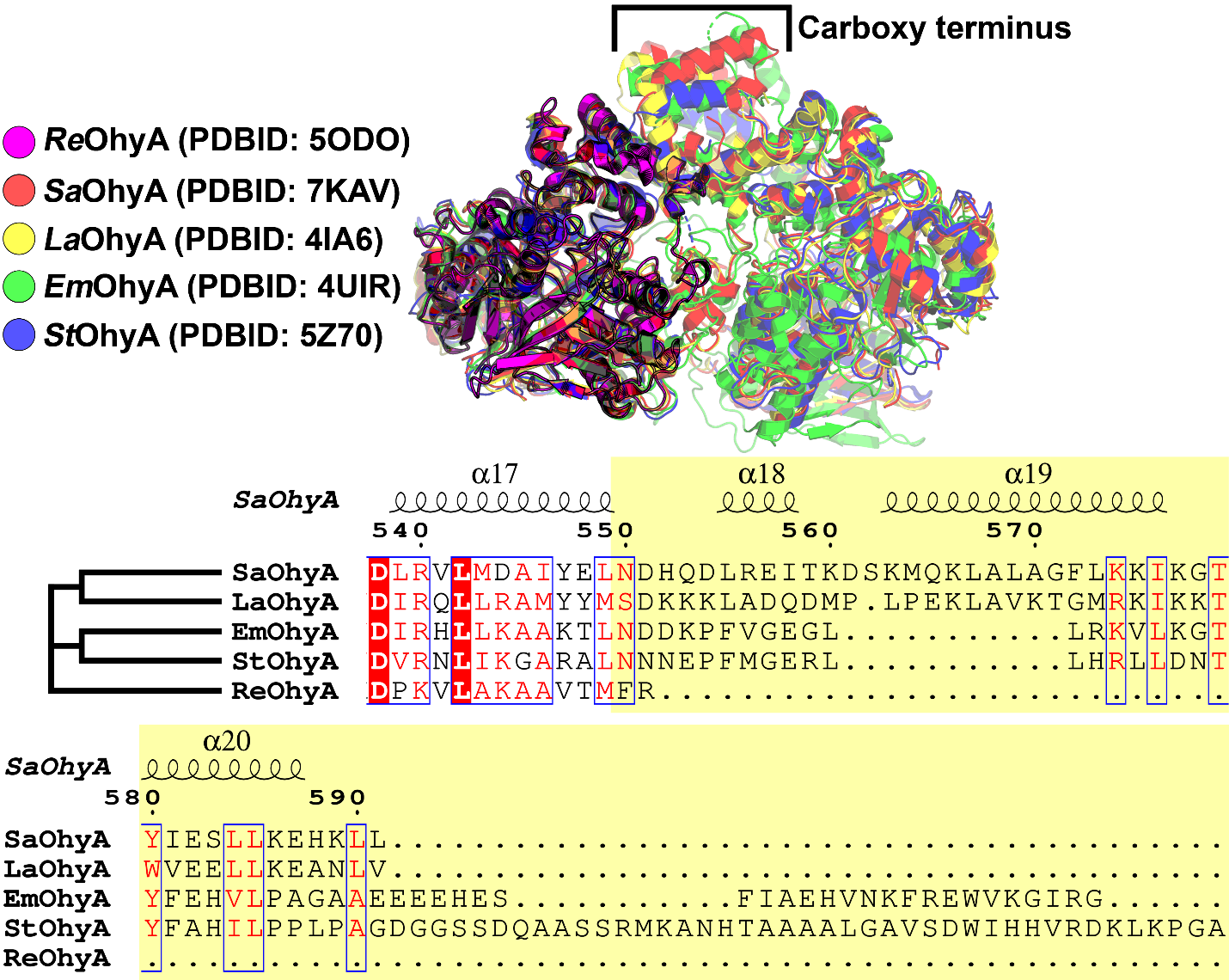


**Supplemental Figure 20. OhyA carboxy terminus diversity.** Crystal structure alignment (*top panel*) of *Rhodococcus erythropolis* OhyA (*Re*OhyA), *Staphylococcus aureus* OhyA (*Sa*OhyA), *Lactobacillus acidophilus* OhyA (*La*OhyA), *Elizabethkingia meningoseptica* OhyA (*Em*OhyA), and *Stenotrophomonas* OhyA (*St*OhyA). All structures aligned against *Sa*OhyA with an RMSD of 0.4 – 1.1 Å. *Sa*OhyA secondary structure mapped onto the alignment of the amino acid sequence from the crystal structures (*bottom* *panel*). The amino acid sequence corresponding to the carboxy terminus is indicated by yellow highlight. Clustal Omega (<https://www.ebi.ac.uk/Tools/msa/clustalo/>) was used for primary sequence alignment and phylogenetic analysis. The *ESPript* program (<https://endscript.ibcp.fr/ESPript/ESPript/index.php>) was used to visualize the sequence conservation and map *Sa*OhyA secondary structure elements onto the alignment. Sequence numbering is assigned to *Sa*OhyA.


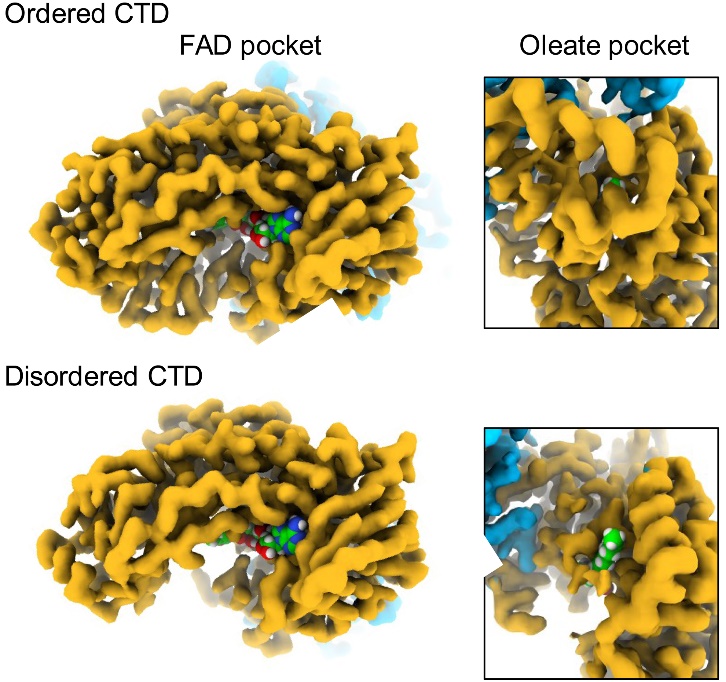


**Supplemental Figure 21. 3D variability analysis of the cryo-EM OhyA dimer shows variability in the substrate binding pockets.** Comparison of the FAD and oleate binding pockets for the ordered and disordered CTD reconstructions. FAD and oleate ligands are placed into the reconstructions based on the superpositions of their parent structures (PDB ID: 7KAW and 7KAY) with the cryo-EM dimer reconstructions from this study.
